# Genome-Wide Analysis and Cloning of the Apple Stress-Associated Protein Gene Family Reveals *MdSAP15*, Which Confers Tolerance to Drought and Osmotic Stresses in Transgenic *Arabidopsis*

**DOI:** 10.3390/ijms19092478

**Published:** 2018-08-21

**Authors:** Qinglong Dong, Dingyue Duan, Shuang Zhao, Bingyao Xu, Jiawei Luo, Qian Wang, Dong Huang, Changhai Liu, Chao Li, Xiaoqing Gong, Ke Mao, Fengwang Ma

**Affiliations:** State Key Laboratory of Crop Stress Biology for Arid Areas/Shaanxi Key Laboratory of Apple, College of Horticulture, Northwest A & F University, Yangling 712100, China; dong19850412@163.com (Q.D.); duandingyue207@foxmail.com (D.D.); zhsh812972738@126.com (S.Z.); 18392610250@163.com (B.X.); m18391488428@163.com (J.L.); wangqian123@nwafu.edu.cn (Q.W.); Mrhaodee@126.com (D.H.); chliu@nwafu.edu.cn (C.L.); lc453@163.com (C.L.); gongxq0103@nwsuaf.edu.cn (X.G.)

**Keywords:** apple, SAP gene family, expression analysis, function analysis, drought stress, osmotic stress

## Abstract

Stress-associated proteins (SAPs) are novel A20/AN1 zinc finger domain-containing proteins that are now favorable targets to improve abiotic stress tolerance in plants. However, the SAP gene family and their biological functions have not been identified in the important fruit crop apple (*Malus* × *domestica* Borkh.). We conducted a genome-wide analysis and cloning of this gene family in apple and determined that the overexpression of *MdSAP15* enhances drought tolerance in *Arabidopsis* plants. We identified 30 SAP genes in the apple genome. Phylogenetic analysis revealed two major groups within that family. Results from sequence alignments and analyses of 3D structures, phylogenetics, genomics structure, and conserved domains indicated that apple SAPs are highly and structurally conserved. Comprehensive qRT-PCR analysis found various expression patterns for *MdSAPs* in different tissues and in response to a water deficit. A transgenic analysis showed that the overexpression of *MdSAP15* in transgenic *Arabidopsis* plants markedly enhanced their tolerance to osmotic and drought stresses. Our results demonstrate that the SAP genes are highly conserved in plant species, and that *MdSAP15* can be used as a target gene in genetic engineering approaches to improve drought tolerance.

## 1. Introduction

The growth, development, and survival of plants is constantly challenged by a variety of biotic and abiotic environmental factors. Plants utilize complex molecular mechanisms that regulate patterns of gene expression to protect themselves against these stresses [1,2]. Some key modulators of stress responses have been characterized and have emerged as appropriate targets to enhance abiotic stress tolerance in many plants. They include NAC domain-containing transcription factors, DRE/CRT-binding transcription factors (DREBs/CBFs), mitogen-activated protein kinases (MAPKs), stress-associated proteins (SAPs), and heat shock factor/proteins (HSPs/HSF) [1,3,4,5,6]. Among these, the SAPs are a newly identified class of zinc finger proteins (ZFPs) that play crucial roles in various abiotic stress responses by numerous plants [1,2,7].

The SAP gene family members have two special ZF domains: the highly conserved A20 domain, which was first isolated in human umbilical vein endothelial cells with the characterization of a tumor necrosis factor (TNF)-α-inducible protein; and/or the AN1 domain, which is also highly conserved and first identified from *Xenopus laevis* animal hemisphere 1 (AN1) maternal RNA with the delineation of the ubiquitin-like protein [8,9]. The SAP proteins expressed in *Arabidopsis thaliana* (hereafter *Arabidopsis*), rice (*Oryza sativa*), tomato (*Solanum lycopersicum*), and cotton (*Gossypium hirsutum*) have been classified into five groups (I through V) based on results from their phylogenetic analyses [10,11]. One significant feature of plant SAPs is the very frequent occurrence of intronless genes [2]. For example, 11 rice SAP genes, 15 from desert poplar (*Populus euphratica*), and 30 from cotton lack introns and show a remarkably higher percentage of intronless genes [2,6,11].

The roles of SAP genes are increasingly being reported in plants. Transcriptional levels are induced by multiple stresses and provide a positive reinforcement of tolerance to abiotic stress. Rice A20/AN1 protein (OSISAP1/OsSAP1), the first identified plant SAP gene, is induced after different types of stress treatments are applied [1]. The overexpression of *OSISAP1* confers tolerance to dehydration, cold, and salt in transgenic seedlings of tobacco (*Nicotiana tabacum*) [1]. Furthermore, *ZFP177* (*OsSAP9*) and *AtSAP5* are induced by numerous challenges and have significant roles in improving abiotic stress tolerance [12,13]. Similar results have been described for SAPs from maize (*Zea mays*) [14], medicago (*Medicago truncatula*) [15], banana (*Musa* sp.) [16], the halophyte grass *Aeluropus littoralis* [17], and poplar (*Populus alba* × *P. glandulosa*) [18]. These genes also function in biotic stress responses. For example, Tyagi et al. [19] have analyzed the expression patterns of rice SAP gene family members in response to pathogen elicitors, and discovered that *OsSAP1*, *OsSAP8*, and *OsSAP11* are up-regulated. Transgenic tobacco overexpressing *OsiSAP1* shows significantly enhanced basal resistance against infection by the bacterial pathogen *Pseudomonas syringae* pv. *Tabaci* [19].

The SAP genes also help regulate signal transduction and phytohormone synthesis. In rice, the overexpression of *OsDOG* (*OsiSAP11*) [20] and *OsZFP185* (*OsiSAP4*) [21] results in dwarf phenotypes, a decrease in gibberellic acid (GA) contents, and deficient cell elongation. Furthermore, *OsZFP185* negatively regulates the expression of several genes related to abscisic acid (ABA) biosynthesis, and interferes with ABA-mediated tolerance to salt, drought, and cold [21]. Various SAPs can function as E3 ubiquitin ligases, redox sensors, and/or regulators of gene expression under stress [7,13,22,23]. Other novel biological functions for SAP genes will continue to be reported.

Based on their highly conserved A20/AN1 domains, members of the SAP gene family have been identified and characterized in *Arabidopsis* [24], rice [24], maize [14], tomato [10], cotton [11], desert poplar [6], and medicago [25]. Although extensive genomic analyses have provided considerable details about this family in several species, members in apple (*Malus* × *domestica* Borkh.) have not been as thoroughly investigated. Nevertheless, recent completion of the draft genome sequence for apple has enabled genome-wide analyses of its SAP genes [26,27,28]. Here, we identified SAP members in apple and examined their A20/AN1 domain, protein and gene structures, conserved domains, phylogenetic relationships, chromosomal locations, *cis*-acting elements, and expression patterns for *MdSAPs* cloned in response to water deficits. We also overexpressed *MdSAP10* in *Arabidopsis* and investigated its function. Our results will serve as a basis for exploring the molecular roles of SAPs. By facilitating further studies into their functions in abiotic stress responses, we can continue our efforts to introduce improved apple cultivars.

## 2. Results

### 2.1. Identification and Annotation of Apple SAP Genes

To identify the genes in the apple genome that encode SAP proteins, we conducted a BlastP of the apple genome database and identified 32 putative family members. We then used the Pfam and NCBI Conserved Domain Database (NCBI-CDD) databases for verification, searching for the A20/AN1 domain in the amino acid sequences encoded by all 32 genes. From this, we confirmed the identity of 30 typical apple SAP genes in the original dataset (Table 1).

We next cloned all of the full-length apple SAP genes based on predicted nucleotide sequences in the apple genome and in the NCBI nucleotide database. As shown in Table 1, this revealed that the full-length cDNAs of *MdSAP7*, -*8*, -*10*, -*12*, -*14*, -*15*, -*16*, -*19*, -*21*, -*23*, -*25*, -*28*, and -*29* had been isolated and confirmed by RT-PCR (Supplementary Sequence A1). Their corresponding 5′- and 3′-UTRs were then amplified.

### 2.2. Structures and Conserved Domains of Apple SAP Genes

**Figure 1:**
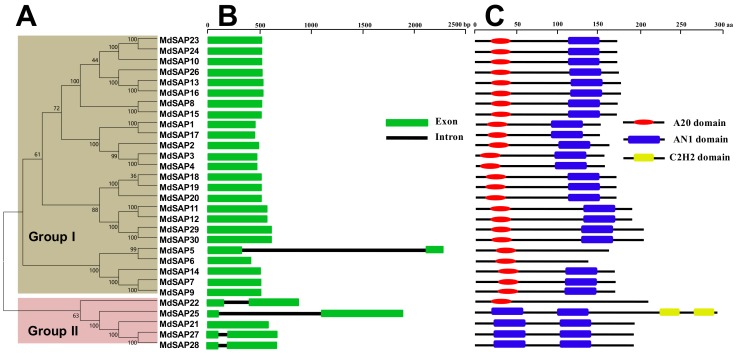
(**A**) Phylogenetic relationships; (**B**) Structures for 30 genes; and (**C**) Analysis of conserved domains for stress-associated protein (SAP) genes in apple. A phylogenetic tree for full-length amino acid sequences was constructed with MEGA software and the NJ method.

**Figure 2:**
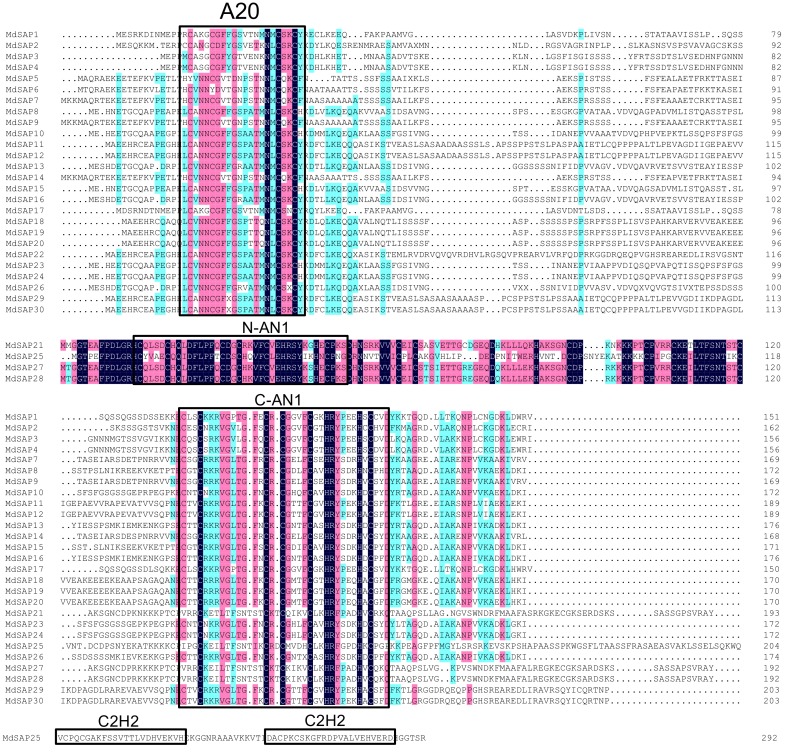
Multiple alignments of A20/AN1 domain and C2H2 amino acids in apple SAPs, using the DNAMAN program. Conserved domains are boxed, and identical amino acids are shown against a dark blue background (Similarity: dark blue = 100%; pink > 75%; cyan > 50%).

To gain insights into the structural diversity of SAP genes in apple, we analyzed the phylogenetic tree, exon–intron organization, and conserved domains in the coding sequences. The *MdSAP* proteins were classified as groups I and II based on their phylogenetic relationships (Figure 1A). Gene structure analysis indicated that *MdSAP1* through *MdSAP4*, *MdSAP6* through *MdSAP21*, *MdSAP23*, *MdSAP24*, *MdSAP26*, *MdSAP29*, and *MdSAP30* contained no introns, whereas *MdSAP5*, *MdSAP22*, *MdSAP25*, *MdSAP27*, and *MdSAP28* had one each (Figure 1B). Conserved domain analysis revealed that all of the *MdSAP* proteins included A20 and/or AN1 domain(s). *MdSAP1* through *MdSAP4*, *MdSAP7* through *MdSAP20*, *MdSAP23*, *MdSAP24*, *MdSAP26*, *MdSAP29*, and *MdSAP30* contained an A20 domain and an AN1 domain; *MdSAP5*, *MdSAP6*, and *MdSAP22* had single AN1 domains; and *MdSAP21*, *MdSAP25*, *MdSAP27*, and *MdSAP28* each had two AN1 domains. In addition, *MdSAP25* contained a C2H2 domain at the C terminal (Figure 1C and Figure 2).

### 2.3. Multiple Sequence Alignments and Three-Dimensional Structure of Apple A20/AN1 Domains

**Figure 3:**
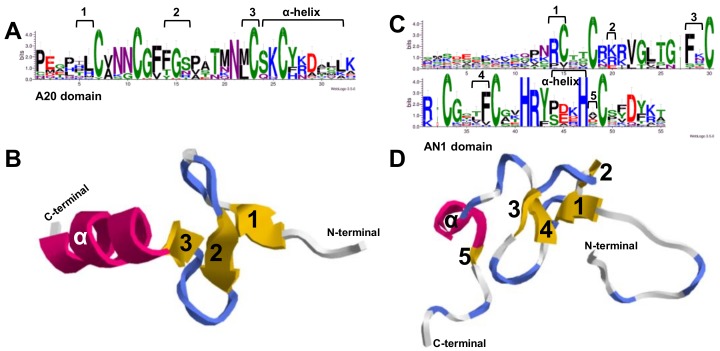
(**A**) Sequence logos for A20 domain in 26 *MdSAP* proteins, generated via WebLogo; (**B**) Three-dimensional tertiary structural model of A20 domain (PDB ID: 2KZY.1.A); (**C**) Sequence logos of AN1 domain in 28 *MdSAP* proteins; and (**D**) Three-dimensional tertiary structural model of AN1 domain (PDB ID: 1WFP.1.A). Within each stack, symbol height indicates the relative frequency of each amino acid at that position. Logos for the A20 domain and AN1 domain were obtained through multiple alignments of 26 and 28 *MdSAP* protein sequences, respectively. At the top of the corresponding amino acid sequences, arabic numbers (1–5) indicate β-sheets in A20 and AN1 domains. In (**B**,**D**), α-helices are red, β-sheets (arabic numbers 1–5) are yellow, and strands are blue/gray. Three-dimensional representations were generated with RasTop software.

Multiple alignments demonstrated that the A20/AN1 domains are conserved among the *MdSAP* proteins (Figure 2). We then produced sequence logos that further showed that these domains were highly conserved at each residue position (Figure 3A,C; Supplementary Sequences A2 and A3). Afterward, the SWISS-MODEL web server was used for modeling and analysis of homology among protein structures. For this, we built the A20 domain and AN1 domain homology models and evaluated them using the homologous templates 2KZY.pdb and 1WFP, respectively (Figure 3B,D). The 3D models indicated that, respectively, the A20 domain and the AN1 domain in the *MdSAP7* structure most closely matched the A20 domain of ubiquitin receptor ZNF216 and the zf-AN1 domain of the *Arabidopsis* F5O11.17 protein (PDB ID: 2KZY.1.A, 38% sequence identity for residues 18–50; 1WFP.1.A, 47% sequence identity for residues 96–148).

### 2.4. Phylogenetic Analysis of SAP Proteins

**Figure 4:**
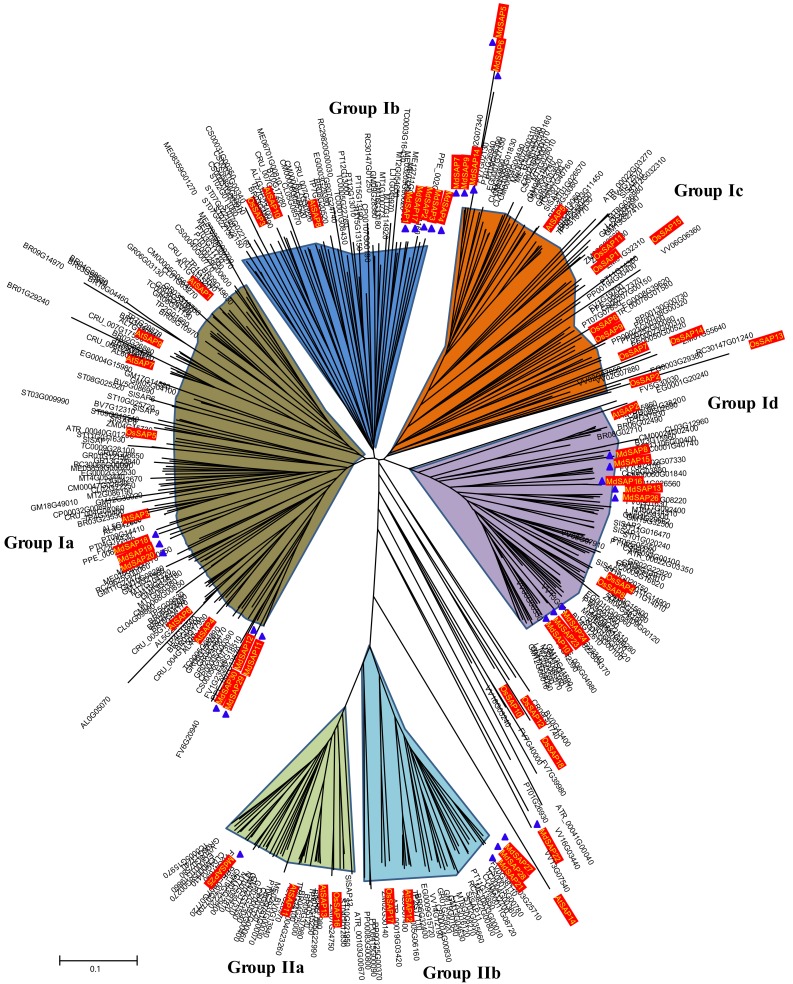
Phylogenetic analysis of 453 SAP proteins in 32 species. Unrooted NJ tree was constructed with MEGA 6 software, using full-length amino acid sequences. Tree comprises six subgroups (Ia–Id; IIa and IIb).

To examine the evolutionary relationships among plant SAP proteins, we used MEGA 6 and constructed unrooted phylogenetic trees from full-length protein sequences encoded by 453 SAP genes in 32 species (Supplementary File B1). Two major groups were revealed: I, containing an A20 domain and an AN1 domain; and II, containing two AN1 domains. Members in Group I were further classified into four subgroups (Ia-Id), while Group II members were assigned to two subgroups: IIa, containing two AN1 domains and one or two C2H2 domain(s); and IIb, containing only two AN1 domains. Among these 30 *MdSAP* proteins, 25 (*MdSAP1*-*MdSAP20*, *MdSAP23*, *MdSAP24*, *MdSAP26*, *MdSAP29*, and *MdSAP30*) could be unambiguously classified as Group I, while four (*MdSAP21*, *MdSAP25*, *MdSAP27*, and *MdSAP28*) were assigned to Group II based on their relationship with the other SAP proteins (Figure 4 and Supplementary File B2). Further analysis revealed that the 25 Group-I apple proteins belonged to subgroups Ia (seven genes), Ib (five), Ic (five), and Id (eight). Subgroup IIa contained *MdSAP25*, while IIb contained *MdSAP21*, *MdSAP27*, and *MdSAP28*.

### 2.5. Genome Distribution of Apple SAP Genes

**Figure 5:**
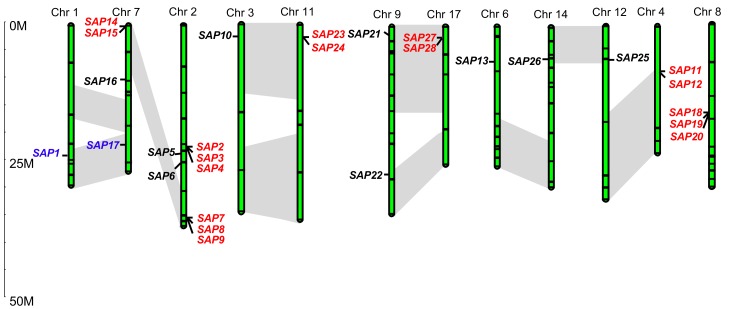
Chromosomal locations of 28 apple SAP genes. Scale is in megabases (Mb). Red font, tandem duplication; blue font, segmental duplication; grey area, genome-wide duplications.

We determined the genomic locations of these apple SAP genes based on their mapping coordinates. In all, 28 of the 30 *MdSAP* genes were assigned to chromosomes 1–4, 6–9, 11, 12, 14, and 17 (Table 1; Figure 5). However, we could not conclusively map two genes (*MdSAP29* and *MdSAP30*) to any chromosome. The genes were unevenly distributed among the 12 chromosomes, with Chromosome 2 containing the most (eight genes), followed by Chromosome 7 (five genes), and one each for chromosomes 1, 3, 6, 12, and 14.

The apple gene family appears to have expanded during the process of genome evolution [26]. To uncover the mechanism underlying this expansion, we investigated gene duplication events, including tandem and segmental duplications, and found that many *MdSAP* genes (19/30, or 63.33%) were present in two or more copies (Figure 5). In all, 17 had undergone tandem duplication, while two were subjected to segment duplication. Those segment duplications produced many homologs of SAP genes on different chromosomes, while tandem duplications produced SAP gene clusters or hotspots (blue and red font in Figure 5). A relatively recent genome-wide duplication is that in the Pyreae tribe, which was thought to result in the transition of nine ancestral chromosomes to 17 chromosomes [26]. We noted here that multiple gene pairs were each linked to at least six potential chromosomal segmental duplications (Figure 5, pairs of bars in grey areas), e.g., large sections of chromosomes 9 and 17, 3 and 11, and 7 and 2.

### 2.6. Promoter Sequence Analysis of Apple SAP Genes

To investigate putative *cis*-acting elements in their promoter regions, we isolated approximately 1500-bp genomic sequences upstream of the start codon from our *MdSAPs*. Along with some *cis* elements involved in light-responsiveness (Table 2 and Supplementary File B3), we found that many were responsive to various stresses and correlative hormones. In total, 10 types of *cis* elements were discovered in the 13 promoters. They were associated with responses to hypoxia, heat, chilling, drought, pathogens, wounding, or hormones such as salicylic acid, methyl jasmonate, ABA, or ethylene. Therefore, we concluded that these *cis* elements play important roles in plant stress responses.

Sequences and functions for ABRE (ABA response element), ARE (anaerobic response element), CGTCA (MeJA-responsiveness), ERE (ethylene-responsive element), HSE (heat shock response element), LTR (low-temperature response element), MBS (MYB binding site involved in drought response), TCA (salicylic acid response element), TC-rich repeat (defense and stress responsiveness), and W-box (elicitation; wounding and pathogen responsiveness; binding site of WRKY type transcription factors) were obtained from the PlantCARE database (http://bioinformatics.psb.ugent.be/webtools/plantcare/html/). Digits represent the number of regulatory elements on plus/minus strand. Blank space indicates no corresponding cis-acting element in either strand of the promoter.

### 2.7. Expression Profiles of MdSAP Genes

**Figure 6:**
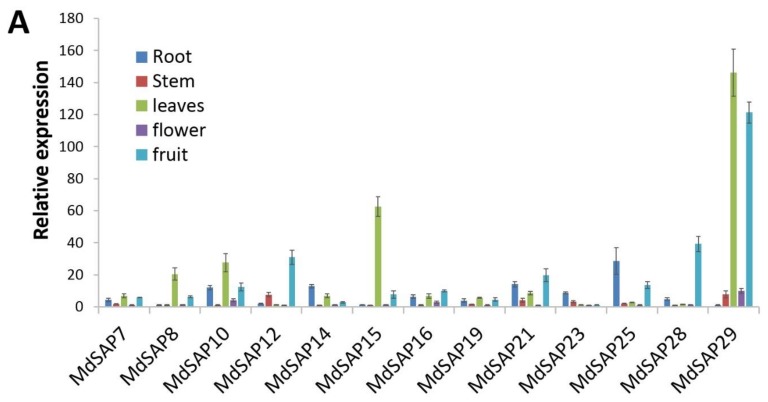

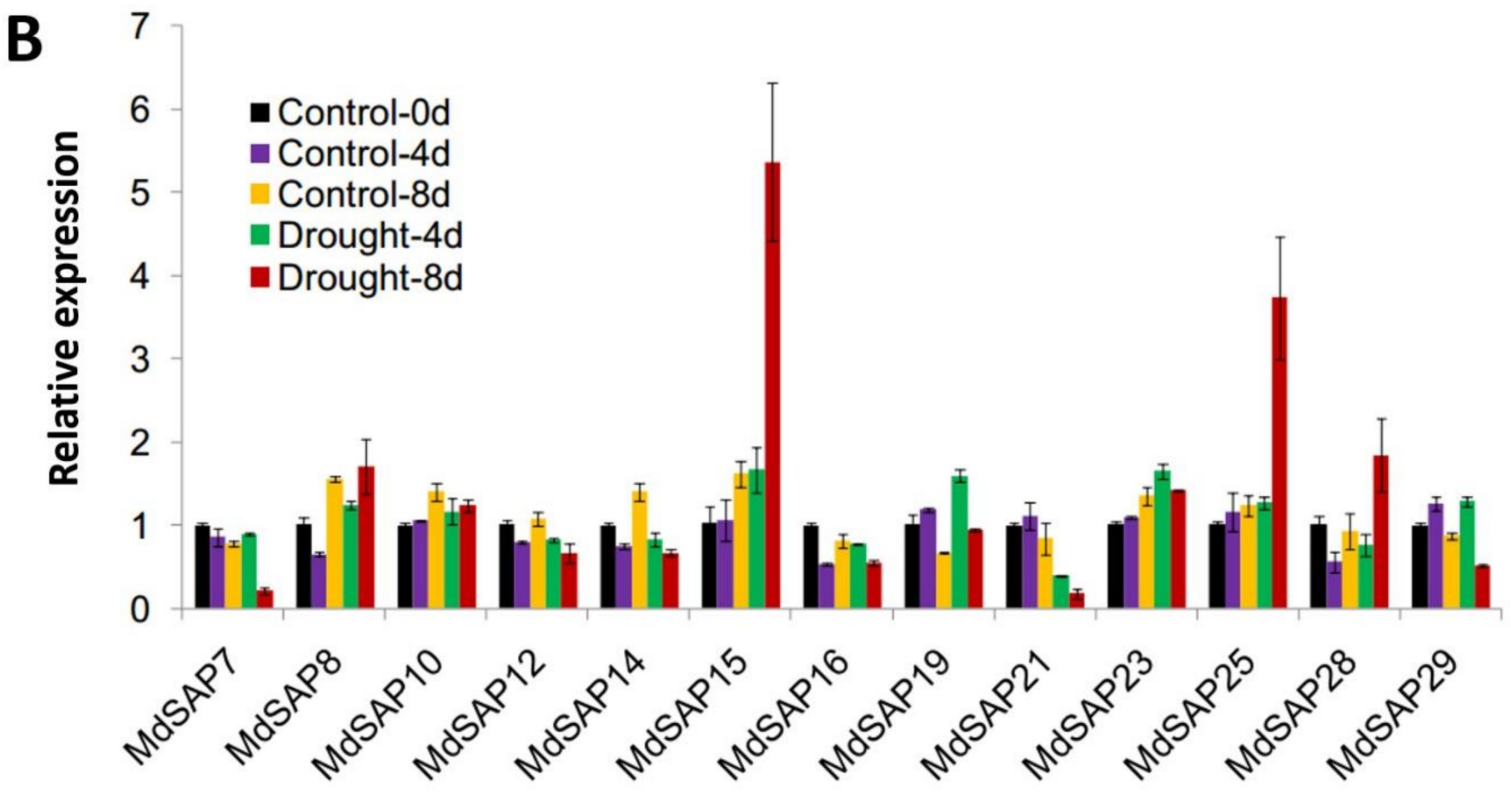
Tissue-specific expression and drought response of 13 *MdSAP* genes. (**A**) Expression patterns of 13 *MdSAP* genes in apple tissues. (**B**) Expression patterns of 13 *MdSAP* genes in response to drought stress, i.e., normally scheduled irrigation withheld for 0, 4, or 8 days. Three independent replicates were used for calculations. Error bars indicate standard deviation.

Knowing the patterns of expression in various tissue types can help us understand gene functions. Firstly, we collected different tissues including young roots, stems, fully expanded leaves, flowers, and mature fruit (70 mm, red peel, 150 days after bloom), from apple plants that were five years old after bud grafting. The scion was *Malus domestica* “Golden Delicious”, and the rootstock was *M. hupehensis*. Then, we isolated the full-length cDNA, 5′-UTR, and 3′-UTR sequences for 13 *MdSAP* genes and used specific primers for our qRT-PCR assays. These *MdSAP*s were constitutively expressed in the five tissues examined here, albeit at different levels of transcription (Figure 6A). For example, *MdSAP7*, -*8*, -*10*, -*15*, -*19*, and -*29* were most highly expressed in the leaves; *MdSAP12*, -*16*, -*21*, and -*28* were most highly expressed in the fruits; and *MdSAP14*, -*23*, and -*25* were most highly expressed in the roots. To induce a water deficit, irrigation was withheld up to 8 days, while the designated control plants continued to receive normally scheduled irrigation (“Golden Delicious” scions and *M. hupehensis* rootstocks). In response to drought stress, the expression of *MdSAP15*, -*25*, and -*28* was significantly induced from that detected in the non-stressed control plants (Figure 6B), transcripts of *MdSAP7* and -*21* mRNAs were significantly reduced, and expression of the other MdSAP genes remained constant.

### 2.8. MdSAP15 Overexpression Enhances Osmotic Stress Tolerance by Arabidopsis Seedlings

**Figure 7:**
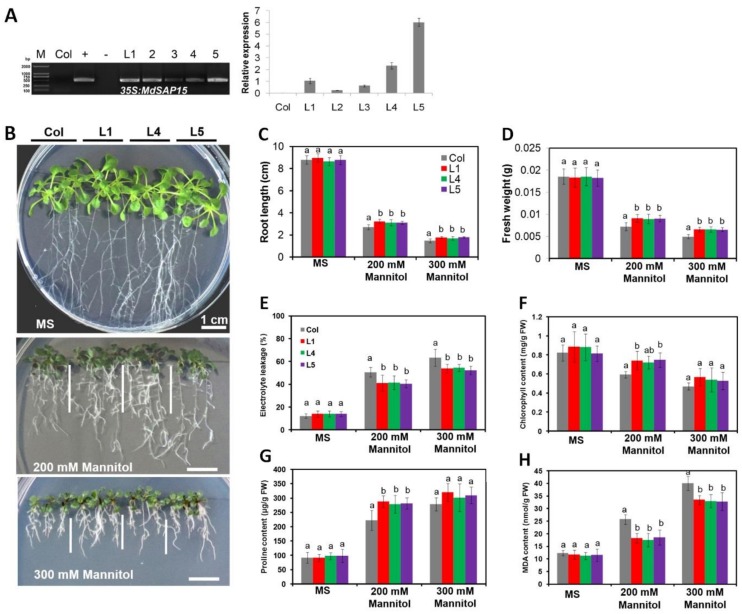
Overexpression of *MdSAP15* in *Arabidopsis* increased osmotic tolerance in response to mannitol treatment. (**A**) PCR identification and relative expression analysis of *MdSAP15* in Col-0 wild-type (Col) and transgenic *Arabidopsis* lines. M, DNA marker; −, negative control (H2O); +, positive control (plasmid DNA of *35S::MdSAP15* pRI101AN vector). Specific primers for *MdSAP15* were used to detect relative expression levels of Col and transgenic *Arabidopsis* lines. (**B**) Representative images of “Col” and transgenic seedlings at 5 days after seeds had been cultivated for 11 days on MS medium alone (0 mM mannitol) or MS medium supplemented with 200 or 300 mM of mannitol. Bars = 1 cm. (**C**) Primary root lengths and (**D**) fresh weights of “Col” and transgenic seedlings measured on Day 5 after plants had been exposed for 11 days to osmotic stress. (**E**) Relative electrolyte leakage, and levels of chlorophyll (**F**), proline (**G**), and malondialdehyde (MDA) (**H**) in “Col” and transgenic seedlings, measured on Day 5 following treatment on MS medium with 0 mM, 200 mM, or 300 mM of mannitol for 11 d. Error bars represent SD based on three independent replicates. For (**C**–**H**), bars not labeled with same letters in each panel indicate values are significantly different at *p* < 0.05, based on one-way ANOVA and Duncan’s tests.

Since the transcription of *MdSAP15* mRNA was significantly accumulated under drought stress, we chose this gene for investigating its biological functions in *Arabidopsis*. After kanamycin-resistance screening and PCR detection using *Arabidopsis* genomic DNAs as templates, more than five transformants were identified and confirmed, with elevated levels of *MdSAP15* transcripts (Figure 7A). From these, we selected three transgenic lines (L1, 4, and 5) with high *MdSAP15* expression to evaluate its potential functioning in response to osmotic and drought stresses. For the osmotic stress assay, five-day-old seedlings grown on Murashige and Skoog (MS) agar plates were vertically plated on an MS agar medium supplemented with 0, 200, or 300 mM of mannitol. While the roots of “Col” and *MdSAP15* OE seedlings displayed similar growth characteristics on MS agar medium plates (Figure 7B), their growth was affected when treated with different concentrations of mannitol. For example, the primary roots and fresh weights of the transgenics were longer and heavier than those of the wild-type (WT) “Col” upon exposure to 200 or 300 mM of mannitol (Figure 7C,D).

### 2.9. MdSAP15-Overexpressing Arabidopsis Seedlings Have Improved Physiological Traits Associated with Osmotic Stress Tolerance When Compared with “Col” Wild Type

For further investigation of this *MdSAP15*-mediated enhancement of tolerance to osmotic stress, we measured relative electrolyte leakage (REL) and concentrations of chlorophyll, proline, and malondialdehyde (MDA), all of which are important markers of such tolerance. Under normal growing conditions, we did not observe any obvious differences in REL. However, in response to mannitol exposure, REL values were significantly higher in “Col” seedlings than in the transgenics (Figure 7E). Levels of chlorophyll and proline were similar between “Col” and OE plants under control conditions, but were higher in the transgenics after they were treated with 200 mM of mannitol (Figure 7F,G). Furthermore, MDA concentrations did not differ among genotypes under normal conditions, but were lower in the overexpressed (OE) lines than in the WT following mannitol treatment (Figure 7H). These results suggested that the overexpression of *MdSAP15* in *Arabidopsis* seedlings leads to enhanced osmotic stress tolerance.

### 2.10. MdSAP15 Overexpression Enhances Drought Tolerance in Transgenic Arabidopsis Plants

**Figure 8:**
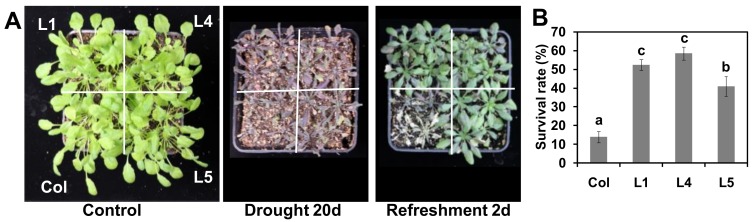
Assessment of drought tolerance in “Col” and transgenic *Arabidopsis* plants. (**A**) Representative images of four-week-old plants; (**B**) Survival rates of WT and transgenic lines 2 days after rehydration began. Error bars represent SD based on three independent replicates. For (**B**), bars not labeled with same letters in each panel indicate values are significantly different at *p* < 0.05, based on one-way ANOVA and Duncan’s tests.

To evaluate drought tolerance, we simultaneously germinated seeds of the “Col” and *MdSAP15*-overexpressing lines and grew the seedlings on MS plates for one week before transplanting them into soil for another three weeks of culture. Drought conditions were then imposed by withholding water. After 20 days of stress, all of the plants exhibited symptoms related to severe water loss, although those symptoms were milder for the OE transgenics than for the WT. When rehydration began (2 days of refreshment), most of the “Col” plants did not recover (Figure 8A), whereas the survival rate was significantly higher for the three transgenic lines (Figure 8B). These results again indicated that the overexpression of *MdSAP15* in *Arabidopsis* plants enhances their degree of drought tolerance.

## 3. Discussion

The proteins encoded by SAP genes comprise large families and are broadly distributed in higher plants [2]. Apple is an economically important woody plant and the most widely cultivated fruit crop in the world. Sequencing of its genome has provided a good platform for genome-wide analyses of all putative gene families in apple, including the DREB [29], MYB [30], MADS-box [31], and WRKY [32,33] families. However, genome-wide information about apple SAP genes has remained unknown, while members of that family have been identified in other plant species [6,10,11,14,24,25]. Moreover, the content of SAP genes varies substantially among species. For example, *Brassica rapa*, *Glycine max*, *Solanum tuberosum*, *Salix purpurea*, *Populus trichocarpa*, and cotton each have a relatively large number of SAP members, i.e., 28, 26, 19, 19, 19, and 19, respectively; while *Chlamydomonas reinhardtii*, *Lotus japonicus*, *Carica papaya*, and *Amborella trichopoda* have relatively few, i.e., 2, 6, 7, and 7, respectively (Table 3). Here, we determined that the apple genome contains 30 SAP genes, making this family much larger than in any other species.

Segmental, tandem, and whole-genome duplications are critical for both the diversification of gene functions and the rearrangement and expansion of genomes [31,32,33,34]. Whole-genome duplication events have occurred in apple [26], and tandem, segmental, and whole-genome duplications have caused some apple gene families to expand, including the MYB [30], MADS-box [31], and WRKY [32] families. We learned here that two *MdSAP* genes have undergone segmental duplication, and 17 have undergone tandem duplication. In addition, multiple gene pairs have each been linked to six potential chromosomal segmental duplications (Figure 5). Similar results have been reported for the Medicago SAP gene family. Our findings suggest that transposition events and the whole-genome and chromosomal segmental duplications have led to the expansion of the apple SAP gene family, and might partially explain why more SAP genes are present in apple than in any other species.

In several distinct species, the zinc finger types of some family members have either disappeared or increased in number. For example, the A20-A20-AN1 zinc finger occurs only in rice and *Eucalyptus grandis*; the A20 type is found in apple, rice, *Amborella trichopoda*, *B. rapa*, and grape (*Vitis vinifera*); the AN1-AN1-C2H2 zinc-finger exists in *Arabidopsis thaliana*, *A. lyrata*, *B. rapa*, *Capsella rubella*, *Thellungiella parvula*, and desert poplar (Table 3). We might speculate that the loss or the increase in zinc finger types of SAP genes in these genomes means that they are critical for the complicated enzymatic activity that is present in those species. In this study, we determined that apple SAPs are highly and structurally conserved based on analyses of gene structure, conserved domains, sequence alignments, 3D structures, and phylogenetics (Figure 1, Figure 2, Figure 3 and Figure 4). Similar results have been reported for *Arabidopsis*, rice, maize, tomato, cotton, desert poplar, and medicago [6,10,11,14,24,25].

Although members of the SAP gene family in *Arabidopsis*, rice, tomato, and cotton are arranged into five groups [10,11,24], the SAP proteins expressed in *Arabidopsis*, desert poplar, *Populus trichocarpa*, *Salix purpurea*, and *S*. *suchowensis* are classified into two major groups: I (Ia–If) and II (IIa and IIb) [6]. Due to the small number of plant species that have been examined, we cannot yet accurately analyze the evolutionary relationships within that gene family. In this study, we were able to divide those members into two major groups (Ia–Id/IIa and IIb) by comparing the apple genome with SAP proteins from 31 other species (Figure 4). We believe that this result from our evolution analysis is convincing. Usually, exon–intron structural diversity can provide important evidence for phylogenetic relationships and play a valuable role in the evolution of gene families [31]. An intronless structure is typical of SAP genes in various species, and is a key characteristic of that family. However, one exception is the grape genome, for which only two VvSAP members lack introns, while 10 members each contain one intron. Furthermore, we discovered here that the intronless gene structure of SAP genes is the dominant arrangement among Group I members, whereas most of the Group II members contain at least one intron (Table 4 and Supplementary File B2). Thus, the prevalence of an intronless gene structure reflects the ancient origin of SAP genes, and links well with their rapid accumulation of transcripts due to reduced post-transcriptional processing [2,35].

Plant SAPs are quickly induced by multiple abiotic stresses [1,12,13,14,36,37,38,39]. They include rice *OsiSAP1*/*OsSAP1*, which responds to drought, salt, cold, submergence, mechanical wounding, and ABA [1]; and *ZFP177* (*OsSAP9*), which is also from rice, and shows enhanced expression in response to cold, heat, and PEG6000 [12]. The expression of *OsiSAP8* in tobacco and rice is enhanced by salt, cold, heat, desiccation, wounding, submergence, heavy metals, and ABA [36]. Similarly, SAP genes in *Aeluropus littoralis*, banana, *Arabidopsis*, and maize respond to salt, cold, drought, and osmotic stresses in a tissue and stress-specific manner [13,14,16,37,38,39]. In this study, we comprehensively analyzed the expression patterns of 13 cloned SAP genes under drought stress. Whereas the expression of *MdSAP15*, -*25*, and -*28* was significantly induced, transcripts levels for *MdSAP7* and -*21* mRNAs were significantly reduced (Figure 6B). Our results suggest that these genes have important roles in the response to water deficits.

The constitutive expression of SAP genes confers tolerance to multiple challenges. The overexpression of rice *OsiSAP1/OsSAP1*, *OsiSAP8*, and *AlSAP* in tobacco and rice increases their tolerance to numerous abiotic stresses [1,17,36,37,40]. Similar findings have been described for the overexpression of *AtSAP5* in cotton and *Arabidopsis* [13,41]. The overexpression of *AtSAP13* and *MusaSAP1* in *Arabidopsis* and banana leads to greater drought and salt tolerances [16,39]. The downregulation of *PagSAP1* improves salt tolerance in poplar and alters the regulation of genes involved in maintaining cellular ionic homeostasis [18]. We noted here that *MdSAP15* overexpression conferred increased tolerance to osmotic stress by increasing the root lengths and fresh weights of transgenic *Arabidopsis* seedlings when compared with the WT. This overexpression also influenced a range of parameters associated with abiotic stress responses, including REL and the concentrations of chlorophyll, proline, and MDA, all of which are often used to evaluate the degree of plant tolerance under stress conditions [42,43,44]. Measured values for all of them were favorably affected in our transgenic lines (Figure 7). Finally, our experiments with induced water deficits demonstrated that the transgenic *Arabidopsis* plants showed milder stress symptoms when compared with the WT, and they also had higher survival rates during the period of rehydration and recovery from drought treatment (Figure 8). Taken together, these results indicate that the overexpression of *MdSAP15* in *Arabidopsis* plants leads to enhanced drought tolerance. This work provides a basis for exploring the molecular roles of SAPs and facilitates further investigations into the functions of these genes in abiotic stress responses. Our data also lay a solid foundation for future efforts to introduce improved apple cultivars.

## 4. Materials and Methods

### 4.1. Identification of Apple SAP Genes

We downloaded the database of the *Arabidopsis* SAP family from the TAIR website (http://www.arabidopsis.org/) [24]. As query sequences for BlastP (http://www.rosaceae.org/tools/ncbi_blast) against predicted apple proteins, we used 14 *Arabidopsis* SAP proteins and the consensus protein sequences of the A20/AN1 domain hidden Markov model (HMM) profile (A20-like zinc finger, PF01754; AN1 zinc finger, PF01428) from the Pfam database (http://pfam.xfam.org/family/PF01754; http://pfam.xfam.org/family/PF01428). We then searched all of those SAP sequences against the apple genome database (https://www.rosaceae.org/gb/gbrowse/malus_x_domestica/) with HMMER v3.0 and BlastP [31,32]. Confirming the reliability of those protein sequences ensured that the A20 and/or AN1 domains were present in each candidate MdSAP protein. For this, we used the Pfam database (http://pfam.sanger.ac.uk/search) and NCBI Conserved Domain Database (NCBI-CDD; http://www.ncbi.nlm.nih.gov/Structure/cdd/wrpsb.cgi) [34].

### 4.2. Sequence Alignments and Phylogenetic Analysis

We performed multiple sequence alignments of 30 *MdSAP* protein sequences, using DNAMAN 6.0.3.99 with its default parameters [45]. A phylogenetic tree for the SAP gene family was constructed with MEGA 6.0 software (www.megasoftware.net) and the neighbor-joining (NJ) method, together with the full-length amino acid sequences of 453 SAPs from 32 plant species, including apple (Supplementary Sequence A2). Related sequences were downloaded from the resource Plaza 3.0 (http://bioinformatics.psb.ugent.be/plaza/). We used the following parameters in the NJ method: bootstrap (1000 replicates), complete deletion, and amino: *p*-distance [34].

### 4.3. Sequence Logos and Structure Model Analysis

Sequence logos for the A20 domain in 26 *MdSAP* genes and the AN1 domain in 28 *MdSAP* genes were generated by the application WebLogo (http://weblogo.threeplusone.com) (Supplementary Sequences A3 and A4). We used the web server SWISS-MODEL (http://swissmodel.expasy.org/) for modeling and predicting the homology of protein structures for those two domains [46]. The proposed 3D structure was modeled on the original NMR structure in PDB ID: 2KZY and 1WFP, and RasTop 2.2 software (http://www.geneinfinity.org/rastop/) was used to present that model [47].

### 4.4. Analyses of Intron–Exon Structure, Genome Distribution, and Gene Duplications

Genomic sequences (apple v1.0), gene distributions on chromosomes, and genome locations of SAPs in apple and 31 other species were downloaded from Plaza 3.0 (Supplementary Sequence A1) [48]. The structural features of these *MdSAP* genes, including exons/introns, numbers, and locations, were obtained and presented by using the gene structure display server (GSDS) web-based bioinformatics tool (http://gsds.cbi.pku.edu.cn/) [49]. The chromosomal positions of all of the *MdSAP* genes were located via MapInspect (www.plantbreeding.wur.nl/UK/software_mapinspect.html) [50]. Segmental and tandem-duplication events were investigated according to the method of Tian et al. [31].

### 4.5. Prediction of Cis-Acting Elements in Promoters

To examine the putative *cis*-acting elements in the promoters of apple SAP genes, we isolated sequences that were 1500 bp upstream of the translational start codon, using the contig sequences of that genome and PCR amplification. Details for the promoters used here are listed in Table 5 and Supplementary File B3. Possible *cis*-acting elements in those promoters were then predicted according to the Plant CARE database (http://bioinformatics.psb.ugent.be/webtools/plantcare/html/) [51].

### 4.6. Plant Materials, Growth Conditions, and Stress Treatments

Young roots, stems, and fully expanded leaves, as well as flowers and mature fruit (70 mm, red peel, 150 days after bloom), were collected from apple plants that were five years old after bud grafting. The scion was *Malus domestica* “Golden Delicious”, and the rootstock was *M*. *hupehensis*. Samples used for examining the effects of water deficits were harvested from plants three months after bud grafting was performed with “Golden Delicious” scions and *M. hupehensis* rootstocks. These grafted plants were grown in pots (height, 320 mm; diameter, 300 mm) in a greenhouse and treatments began when the plants were approximately 500-mm tall. To induce a water deficit, irrigation was withheld from certain plants for up to 8 days while the designated control plants continued to receive normally scheduled irrigation [52]. Our sampling schedule involved harvesting mature leaves at the middle nodes on days 0, 4, and 8 of the deficit period. All of the tissues were frozen immediately in liquid N_2_ and stored at −80 °C.

Seedlings of *Arabidopsis thaliana* L. (Heyn), cv. Columbia (“Col”), were used for genetic transformations and assays of osmotic and drought tolerance. They were cultured in a growth chamber under a 16-h photoperiod at 23 °C. For the drought tolerance assay, water was withheld from four-week-old plants for 20 days before they were rewatered. Survival rates were scored 2 days after rewatering began. Well-watered plants were used as the negative control. For the osmotic stress assay, five-day-old seedlings grown on MS agar plates were vertically plated on an MS agar medium supplemented with 0, 200, or 300 mM of mannitol. Their root lengths, fresh weights, relative electrolyte leakage (REL), and concentrations of chlorophyll, malondialdehyde (MDA), and proline were measured 11 days after that transfer. All of the experiments were repeated three times.

### 4.7. Cloning of MdSAPs and qRT-PCR Analysis

We extracted total RNA from previously frozen apple tissues according to the CTAB method and from *Arabidopsis* leaves, using Trizol reagent (Thermo Fisher Scientific-CN; Shanghai, China; https://www.thermofisher.com/cn/zh/home.html) [53]. Two micrograms of total RNA were collected for synthesizing first-strand cDNA. For cloning *MdSAP*, complete open reading frames were obtained via RT-PCR from fully expanded leaves of “Golden Delicious” apple, using specific primers listed in Table 5. The 5′- and 3′-untranslated regions (UTRs) were obtained with a Rapid Amplification for cDNA Ends kit (TaKaRa, Dalian, China). For the qRT-PCR assays, reverse transcription was performed with 1 μg of total RNA from each sample, followed by PCR-amplification of 1 μL of the product. We conducted the qRT-PCR assays in 20-µL reaction mixtures that contained 10 µL of SYBR^®^ Premix Ex Taq™ (TaKaRa; Beijing, China; http://www.takarabiomed.com.cn), and used an iQ5 instrument (Bio-Rad, Hercules, CA, USA) as described before [34]. Thermal cycling included an initial 3 min at 95 °C; then 40 cycles of 10 s at 95 °C, 30 s at 58 °C, and 15 s at 72 °C; followed by 3 min at 72 °C and then 81 cycles of 7 s each, increasing by an increment of 0.5 °C from 55 °C to 95 °C. Three biological replicates were tested in each assay, and ∆Ct values were calculated by using *MdMDH* as our endogenous control [54]. Relative quantification was calculated according to the 2^−^^∆∆*C*t^ method [55], and dissociation curve analysis was performed for determining the specificity of the amplifications.

### 4.8. Vector Construction and Plant Transformation

To construct the *MdSAP15* overexpression (OE) vectors, we performed RT-PCR to isolate the full-length cDNA of *MdSAP15* from fully expanded leaves of the “Golden Delicious” apple. The cDNA was cloned into pRI 101-AN plant transformation vectors that were driven by the cauliflower mosaic virus (CaMV) 35S promoter. For *Arabidopsis* transformation, the recombinant plasmid described above was introduced into the “Col-0” ecotype via the *Agrobacterium tumefaciens* GV3101-mediated floral dip method. Seeds of the transgenic plants were individually harvested and screened with kanamycin monosulfate. Homozygous transgenic lines were used for further investigations.

### 4.9. Measurements of Physiological Indices

Relative electrolytic leakage was examined as described by Tan et al. [56]. Chlorophyll concentrations were determined using the protocol of Liang et al. [57], while MDA levels were obtained as described by Wei et al. [42]. Proline concentrations were calculated according to the method described by Dong et al. [43].

### 4.10. Statistical Analysis

All of the data were analyzed with IBM SPSS Statistics v.20 software (https://www.ibm.com/support/knowledgecenter/SSLVMB_20.0.0/com.ibm.spss.statistics_20.kc.doc/pv_welcome.html). One-way ANOVA and Duncan’s tests were used to compare the results. Differences between treatments were considered statistically significant at *p* < 0.05.

## Figures and Tables

**Table 1:** Properties of SAPs identified from apple genome.

Gene Name	Gene ID ^1^	Zinc Finger Domain	Protein Length (aa)	Molecular Weight (kDa)	Theoretical Isoeletrical Point	Chromosome Location
*MdSAP1*	MDP0000494946	A20-AN1	151	16.53	8.41	chr1:24227744..24228199
*MdSAP2*	MDP0000588934	A20-AN1	162	17.71	8.62	chr2:22718230..22718718
*MdSAP3*	MDP0000122842	A20-AN1	156	17.07	8.27	chr2:22745813..22746283
*MdSAP4*	MDP0000237812	A20-AN1	156	17.07	8.27	chr2:22753663..22754133
*MdSAP5*	MDP0000316313	A20	161	18.11	8.95	chr2:24190887..24193156
*MdSAP6*	MDP0000543745	A20	136	15.18	6.37	chr2:25865864..25866277
*MdSAP7*	MDP0000362676	A20-AN1	169	18.42	9.05	chr2:35848074..35848583
*MdSAP8*	MDP0000874708	A20-AN1	172	18.24	8.12	chr2:35871562..35872080
*MdSAP9*	MDP0000362677	A20-AN1	169	18.42	9.05	chr2:35878425..35878935
*MdSAP10*	MDP0000164222	A20-AN1	172	18.41	7.52	chr3:2250369..2250887
*MdSAP11*	MDP0000516205	A20-AN1	189	20.09	6.78	chr4:8801881..8802450
*MdSAP12*	MDP0000506127	A20-AN1	189	20.09	6.78	chr4:8807038..8807607
*MdSAP13*	MDP0000286185	A20-AN1	176	18.97	7.46	chr6:7080970..7081500
*MdSAP14*	MDP0000263150	A20-AN1	168	18.51	9.16	chr7:613109..613615
*MdSAP15*	MDP0000292844	A20-AN1	171	18.17	8.12	chr7:697510..698025
*MdSAP16*	MDP0000294781	A20-AN1	176	18.97	7.46	chr7:10610840..10611370
*MdSAP17*	MDP0000133254	A20-AN1	150	16.41	8.41	chr7:22721788..22722240
*MdSAP18*	MDP0000139359	A20-AN1	170	18.59	8.67	chr8:16795514..16796026
*MdSAP19*	MDP0000707978	A20-AN1	170	18.59	8.67	chr8:16801945..16802457
*MdSAP20*	MDP0000296953	A20-AN1	170	18.59	8.67	chr8:16804379..16804891
*MdSAP21*	MDP0000211516	AN1-AN1	193	21.36	8.67	chr9:2267267..2267848
*MdSAP22*	MDP0000165407	A20	209	23.09	8.29	chr9:27693011..27693884
*MdSAP23*	MDP0000231017	A20-AN1	172	18.18	7.51	chr11:2865175..2865693
*MdSAP24*	MDP0000683912	A20-AN1	172	18.18	7.51	chr11:2875969..2876487
*MdSAP25*	MDP0000652898	AN1-AN1-C2H2-C2H2	293	32.04	8.26	chr12:7214047..7215925
*MdSAP26*	MDP0000086327	A20-AN1	174	18.78	7.46	chr14:7829264..7829785
*MdSAP27*	MDP0000141121	AN1-AN1	192	21.39	8.72	chr17:2716099..2716758
*MdSAP28*	MDP0000853499	AN1-AN1	192	21.39	8.72	chr17:2716210..2716869
*MdSAP29*	MDP0000661416	A20-AN1	203	21.86	7.66	unanchored:14381067..14381678
*MdSAP30*	MDP0000284856	A20-AN1	203	21.86	7.66	unanchored:14408647..14409258

^1^ Gene ID in apple genome (https://www.rosaceae.org/gb/gbrowse/malus_x_domestica/).

**Table 2:** The *cis*-acting elements of 13 promoters in apple SAP genes.

*Cis*-Acting Elements	ABRE	ARE	CGTCA	ERE	HSE	LTR	MBS	TCA	TC-Rich Repeat	W-Box
Stress to Response	ABA	Hypoxia	MeJA	Ethylene	Heat	Chilling	Drought	SA	Defense	Pathogen
*MdSAP7*	2/2	2/0	2/2		1/0	1/1		0/2	0/2	
*MdSAP8*		0/1			0/1		3/0	0/3		0/1
*MdSAP10*	2/0	0/2	0/1				1/0	0/2		
*MdSAP12*			1/0			1/0				
*MdSAP14*		2/0	1/1		2/0	1/0		1/0	1/0	0/1
*MdSAP15*	0/1	0/2			0/1		0/1	0/1	1/0	1/0
*MdSAP16*		2/2		2/0					1/0	3/0
*MdSAP19*		0/2		0/1		1/1		1/1	2/0	
*MdSAP21*		1/0	1/0		1/2				1/0	
*MdSAP23*	1/0	1/4	0/2			1/0				1/0
*MdSAP25*	1/1	1/1	1/1			0/2	1/1	0/1	0/1	
*MdSAP28*		0/4	1/2		1/1	1/1	1/1			
*MdSAP29*		0/2	1/2		1/1	1/1		1/0	2/0	

ABA: abscisic acid; ABRE: ABA response element; ARE: anaerobic response element; CGTCA: MeJA-responsiveness; ERE: ethylene-responsive element; HSE: heat shock response element; LTR: low-temperature response element; MBS: MYB binding site involved in drought response; TCA: salicylic acid response element; TC-Rich Repeat: defense and stress responsiveness; W-box: elicitation, wounding, and pathogen responsiveness/binding site of WRKY type transcription factors.

**Table 3:** Numbers of SAP gene family members in various species.

Plant Species	A20-	A20-	A20	AN1	AN1-	AN1-	AN1-	Total Number
	AN1	A20-			AN1	AN1-	AN1-	
		AN1				C2H2	C2H2-	
							C2H2	
*Malus domestica*	23	0	3	0	3	0	1	30
*Arabidopsis thaliana*	10	0	0	1	1	1	1	14
*Oryza sativa*	11	1	1	3	1	0	1	18
*Populus trichocarpa*	15	0	0	2	1	0	1	19
*Solanum lycopersicum*	9	0	0	1	2	0	1	13
*Gossypium hirsutum*	14	0	0	2	2	0	1	19
*Populus euphratica*	15	0	0	0	2	0	1	18
*Arabidopsis lyrata*	12	0	0	0	1	1	1	15
*Amborella trichopoda*	3	0	1	1	1	0	1	7
*Brassica rapa*	18	0	1	5	1	2	1	28
*Beta vulgaris*	6	0	0	1	0	0	1	8
*Citrullus lanatus*	7	0	0	2	1	0	1	11
*Cucumis melo*	10	0	0	0	1	0	1	12
*Carica papaya*	5	0	0	0	1	0	1	7
*Chlamydomonas reinhardtii*	1	0	0	1	0	0	0	2
*Capsella rubella*	10	0	0	0	1	1	1	13
*Citrus sinensis*	10	0	0	0	1	0	1	12
*Eucalyptus grandis*	8	1	0	1	1	0	0	11
*Fragaria vesca*	12	0	0	1	1	0	1	15
*Glycine max*	18	0	0	2	2	0	4	26
*Gossypium raimondii*	14	0	0	2	1	0	2	19
*Lotus japonicus*	4	0	0	1	1	0	0	6
*Manihot esculenta*	14	0	0	1	1	0	1	17
*Medicago truncatula*	11	0	0	2	1	0	2	16
*Physcomitrella patens*	6	0	0	1	2	0	1	10
*Prunus persica*	8	0	0	0	1	0	2	11
*Ricinus communis*	5	0	0	2	1	0	1	9
*Solanum tuberosum*	13	0	0	3	1	0	2	19
*Theobroma cacao*	10	0	0	0	1	0	1	12
*Thellungiella parvula*	11	0	0	0	1	1	1	14
*Vitis vinifera*	4	0	1	4	2	0	0	11
*Zea mays*	8	0	0	1	1	0	1	11

**Table 4:** Statistics for numbers of intronless members within different groups of the SAP gene family.

Group	Ia	Ib	Ic	Id	IIa	IIb
Intronless number	105	43	63	61	1	5
Total number	123	53	74	78	39	34
Percentage (%)	85.36	81.13	85.13	78.2	2.56	14.7

**Table 5:** Application of primers and sequences. ORF: open reading frames.

Use	Primer Name	Forward Primer (5′–3′)	Reverse Primer (5′–3′)
Complete	MdSAP7	ATGAAAAAAATGGCACAGAGAA	TCAAACCCGGACGATCTTTGCGG
ORF	MdSAP8	ATGGAGCACAATGAGACAGGAT	TCAGATTTTATCCAGCTTTTCT
amplification	MdSAP10	ATGGAGCACGAGGAGACTGGATG	TTAGATTTTATCGAGCTTCTCA
	MdSAP12	ATGGCGGAAGAGCACAGATGCG	TCAAATCTTCTCGAGCTTCTCG
	MdSAP14	ATGAAAAAAATGGCACAGAGAA	TCAGAGCCGGACGATCTTCGCA
	MdSAP15	ATGGAGCACAATGAGACAGGATG	TCAGATTTTATCCAGCTTGTCTG
	MdSAP16	ATGGAATCTCATGATGAAACTG	CTAGATTTTGTCAAGTTTGTCTG
	MdSAP19	ATGGCGGAAGAGCATCGTTGCCA	TCAAATCTTATGCAGCTTCTCCG
	MdSAP21	ATGATGGGAGGAACAGAAGCTT	TCAATACGCTCGAACAGATGGCC
	MdSAP23	ATGGAGCACGAGGAGACTGGATG	TTAGATTTTACCAAGCTTGTCAG
	MdSAP25	ATGGGAACTCCGGAATTCCCAGA	CTATGCTCTTGAAGTACCGCCGT
	MdSAP28	ATGACGGGAGGAACAGAAGCTTT	TCAATAAGCTCGAACAGAAGGC
	MdSAP29	ATGGCGGAAGAGCACAGATGCGA	TCACGGATTTGTACGTTGGCAA
Promoter	MdSAP7	CAGATTTTGTTCAAATGTAGG	TGGGCGATGGAGGAGACAGAAAT
amplification	MdSAP8	TGTTTCAATTGCGTTCTTGAGG	CATTGTAATTCGCTTAAGTTCT
	MdSAP10	ACCTTTTCCAAAACCGTTATTAG	TGCGAAAACCAACAATTAATGG
	MdSAP12		
	MdSAP14	GTAAAGAGGTTAGTGGCCCTGAA	CAAATTCTGATCGATCGATCGAT
	MdSAP15	ATGCGCTTTACTGTTTTTTCAGT	CATTGTAATTCGCTAAGTCCTT
	MdSAP16	CACGAGGAGAGCACTAAAATGGA	CACCAAGAAAACCTCGCCGTTT
	MdSAP19	ACCTTTCTTTTGAGAAGTTTGT	TGCAATTCCAAAACAAATTATTC
	MdSAP21	ATGGATTCTAGTTTGATTTGGGC	GATTTTTCAGTTTGTTAAATTTT
	MdSAP23	ATATTTCCATCACATTGAATAA	CTACTCAGCTTACCTGCAAAGAG
	MdSAP25	GCAGGTAGAGTTTCAAAGTACG	AAATTTTGTATGTACAACACTA
	MdSAP28	ACAGGTCACCGTGGTGACTCCGG	GTCGGTCGGTCGGTCTGGGGTTG
	MdSAP29	GTGCTTTTTGTTGGAACACAAAG	CGATCGAGAGGACAAAAATATTA
qRT-PCR	MdSAP7	TCGTCCGGGTTTGATGATTT	TCCCCGGTCTCTGAATTTCG
	MdSAP8	GGGAAGCGGATAGGAACCAT	CTTGGGAGCTTCAGGAGGAG
	MdSAP10	GATTATCGCACTGCTGGACG	AGTGCTAAGATACCGCTGCA
	MdSAP12	GTTGGTCATAGCCGAGAAGC	ATCAGCTTAATTCCCACGCG
	MdSAP14	GCTCTGACCGGTTTGACAAT	TTGCTGATGATCTCCGGGAG
	MdSAP15	ATGATTACCGGACTGCTGCT	CCACATGGGTAGAAATGAGAGC
	MdSAP16	GCCAATCCTATCGTGAAGGC	GAGACCTATGCAGACAAGAAGC
	MdSAP19	CGATTTCAGAGGGATGGGGA	CAACCATCCCCTACCCCAAT
	MdSAP21	AGGGAAAGAATGCGGGAAGA	CGAAGAAACATGAAACTGCGG
	MdSAP23	GCCAACCCTGTCGTAAAAGC	TGCTAAGATACCGCTGCAGA
	MdSAP25	AATCCAATCCAAGCCTCGGA	TCCCATCCGAATTTTGCACG
	MdSAP28	TGCTTTGAGGGAAGGGAAAGA	ACATCGAATTGTGGAAGCAGA
	MdSAP29	TTCCTCCTCGCACAGATCAG	TCCGCCATGTCTACAGTCAA
	MdMDH	CGTGATTGGGTACTTGGAAC	TGGCAAGTGACTGGGAATGA
pRI-101AN	MdSAP15	TTGATACATATGCCCGTCGACATGGAGCACAAT	AGAGTTGTTGATTCAGGATCCTCAGATTTTATC
	35S	CGCACAATCCCACTATCCTT	
	qRT-MdSAP15	AGTCGTTGCAGCATCCATTG	GGAAGCCTGTGTTGAGATAAGC
	AtActin2	GTGAAGGCTGGATTTGCAGGA	AACCTCCGATCCAGACACTGT

## Supplementary Materials

Supplementary materials can be found at http://www.mdpi.com/1422-0067/19/9/2478/s1. Supplementary Sequence A1: Genomic information for cloned *MdSAPs*; Supplementary Sequence A2: Full-length amino acid sequences of 453 SAPs from 32 plant species; Supplementary Sequence A3: Sequence logo for the A20 domain in 26 *MdSAP* genes; Supplementary Sequence A4: Sequence logo for the AN1 domain in 28 *MdSAP* genes; Supplementary File B1: The IDs of SAP gene family members in various species; Supplementary File B2: The intron numbers and groups of SAP gene family members in various species; Supplementary File B3: Prediction of *cis*-acting elements in *MdSAP* promoters.
