# Supplementary material for: Genome-Wide Analysis and Cloning of the Apple Stress-Associated Protein Gene Family Reveals MdSAP15, Which Confers Tolerance to Drought and Osmotic Stresses in Transgenic Arabidopsis
Source: Int J Mol Sci. 2018 Aug 21;19(9):2478. doi: 10.3390/ijms19092478 (PMC6164895; doi:10.3390/ijms19092478)
Supplement: Supplementary file 1 [file ijms-19-02478-s001.zip › ijms-333142-supplementary/Supplementary Sequence A.docx]

**Supplementary:** **Genome-Wide Analysis and Cloning of the Apple Stress-Associated Protein Gene Family Reveals *MdSAP15*, Which Confers Tolerance to Drought and Osmotic Stresses in Transgenic *Arabidopsis***

Qinglong Dong ^†^, Dingyue Duan ^†^, Shuang Zhao, Bingyao Xu, Jiawei Luo, Qian Wang,
Dong Huang, Changhai Liu, Chao Li, Xiaoqing Gong, Ke Mao * and Fengwang Ma *

State Key Laboratory of Crop Stress Biology for Arid Areas/Shaanxi Key Laboratory of Apple, College of Horticulture, Northwest A & F University, Yangling 712100, China; dong19850412@163.com (Q.D.); duandingyue207@foxmail.com (D.D.); zhsh812972738@126.com (S.Z.); 18392610250@163.com (B.X.); m18391488428@163.com (J.L.); wangqian123@nwafu.edu.cn (Q.W.); Mrhaodee@126.com (D.H.); chliu@nwafu.edu.cn (C.L.); lc453@163.com (C.L.); gongxq0103@nwsuaf.edu.cn (X.G.)

***** Correspondence: maoke2002@nwsuaf.edu.cn (K.M.); fwm64@nwsuaf.edu.cn or fwm64@sina.com (F.M.); Tel.: +86-029-8708-2613 (K.M.); +86-029-8708-2648 (F.M.)

† These two authors contributed equally to this work.

**A1. Genomic information for cloned MdSAPs**

>MdSAP7

CAGATTTTGTTCAAATGTAGGAAAATTTAAAGTTAAAGAGGAAGCAATATATTGCTTTTGAAGCATTCTTTTTATATAATAGCTTTTTATAGCAAATCATGATAATTTAAAATCCTCCTTCGGTTTTATATCATCATAATCATAAGAAGCTTTTTGCATGCGGGAGTGTTACTATTTGAACCATTCATCTTAATGGTTCGATAGGATAGTACAAGGAGAGGGTCCAAATATATACATTGAGCCGAGAATGAATCTTAACTTTCAAATTTTGTTTTTGCTTTCTAATGTTTATATTTTTGTAAAAGTAACAATTTGGCCTTACATATAGAGATTTAATAAATTTTTACAATGGTTAAATTTTACATAATACATGTATCACTCAATTTGTAGCCGGACCAGCTTTCTTATACTCCTTGTATCATTGATGAAAAATTACTAGAGCAAATTAAAAACTATAAATCAAAACATAACAAATCCATCTACGATATTTTGAGCTTCTATCGATTTTGAGCTACTAGATTGTCATAACTACTCACTTTAATCCCTGAAATTTAAAATATCACTGAGATTGTCAATTGTTAATCATTTTGATCAGTCCGTAAAAAAATCTTCGCTAAATAGAAAAAACTACAAATTCAAGTGAAGGGGTTGTTTGTGAGAATAATATCCATATGTTAACGAAAATTTTCATATAAAATCAAAATAATTGTCAATAAACAATCTAAGAACTATTATTTTAAATCGTAATAATAGAATTCCATAAAACTAGAGAAATTAGAACATGGGCAACCAATAGAATCAATCGGACGGCCGCATGTTGTAATACGCTGCATGTGGGTAAAAAATAGGGTCCCACCGTACCTTAAAATAAGCTTTGTCAGGATATTTACCTTTTCTCAATAATGACGATTATCCACGTCAAAACAACCAACCAATTTCAATGCGATCGTAATACGTGTGGTACACTCAGTATCACTATATAAATGATAAGATACGTGTGTTAAAAAATTAACAACTTAAAAAATATAACTTTTCACCAATTAAATAAAAACACGTAATGTACTATTCGTGTTCCGATTACAATACAGCAATACCACACCCAAGAACCCAAAAAAAATCAATTATTCCTTCTTTTTATTTAATTTTTTAAGCAAAAAGGTAATAGTAAAAGAAAAGTAGGAAGGGCATTTCGGTAAAACCGAAAGGCAAAAATGGATACGCGTCTCTATGTGGTCCATATTTTCTCGCCAGTACCCGACCTTTCACTCCCTCATATTATCCCCCTCCCCCATTTCTGTCTCCTCCATCGCCCACCCAAACCTCTCCTCTCCTCTCCTCTCTCTCTAAAAACCACAACTTTTTCTTCCTCTCTCTCTGTCTCTCTAGAAAACCACAACCTCCAATTTTCTTCCTTTCCATCCATCTATATCTCTGAAAAAAAACAGAAAAAAAAAAGAGAGAAGACGAGAGAGATCCATCTCTCGATTGTTCAAAATTTGAATGAAAAAAATGGCACAGAGAACGGAGAAGGAAGAGACGGAGTTCAAGGTCCCCGAAACGCTAACGCACTGCGTCAACAACTGCGGCGTCACCGGCAATCCCTCCACCAACAACATGTGCCAGAAGTGCTTCAACGCCGCATCGGCCGCCGCCGCCGCCGCCACTTCATCGTCGTCGTCAGCGGCGATTCTGAAGTTTTCAGCCGAGAAAAGTCCGAGATCTAGCTCATCTTTCAGCTTCGAGGCTGCTGCCGAGACCTGCCGTAAGACGACGGCATCAGAGATCGCGAGATCGGACGAAACGCCGAATCGGCGCGTGGTTAATCGGTGCTCCGGTTGTCGGAGGAAGGTCGGGTTGACCGGATTCAGGTGCCGGTGCGGCGAGCTTTTCTGCTCCGAACACCGGTACTCCGATCGCCACGTGTGCAGTTACGACTATAAGGCTGCCGGTCGCGAGGCCATCGCGAGAGAAAACCCCGTGGTCAAGGCCGCAAAGATCGTCCGGGTTTGATGATTTATATATCGATTGACTTTGTAGAAATGAAGCAACGCAAAAAGTGACGCGAAAATTTGAAGAGGAATTTGTGGCTCGATCGGTTCTCATGGCGCGTCTTTCTCTCGGAGACCAAAACGAAATTCAGAGACCGGGGAGAGATCGCTTCGTTTATCAAATTCTCTCTGATAAT

>MdSAP8

ATTTGTTTCAATTGCGTTCTTGAGGTTTTATGAAAATCAGTAGTTCCATCGAGTTTATAGAAAGACCCTTCGTATTTTCATACGGTGTTGAATTCCCCTTTAAAAAAAAAGGTCCAGGTAAATTAAATTTTCTATGACCGAGAGGGCTACTGTTCTGTCAGTTGTTGTCTTCTGATACCCCAAATCCCGCATACCTTTTGCAGTGGGAAGACAGTGGCTGCTTTAGTAAGGCCTAGGAGTGCAATAGTTAACTTCAACTAGCACTTTTGGATGTGCATTGGGTTGTTGTGACAGTAGACTTGAATTACTGTTGTCTTCCCCACTCTTGAGCTAAATATATGTGGTTGGTGTTTATTTTACTGTGTTTACTCTTTATTACAAAATCATTGCAGCCGAAGTCTGTTCATATTGTTGTTATACCGACTGTCACCTTTACAAGGAGGTAAGACAGTGCTTGTTTACATTAATCTCAGATTTACATTAATGGAACTCCTCAGACAAAATGGCAAGACTGCAAAAGCTCATTCCTTGGTTTTTTATGGGGGTTGCCCTGTTTAATGATTGTAAAAAAAAATTTGCAGAATAAGGAAGTAGGCTTATTTTCTTTTACTTAAAACCTCTGCCTTCCGAGAATTATTATCTTTCGCTAGCCCATCTTTGTCTTTGTCTCTTTCTATCGCCATATCTCTCAAATGGCATTTTTTTTCATAAATCGCCCTCCCTTTGTATGAGGTGAAGGGGGAAATAGATTGGTGGGATAAAAACTTCTTCTGTGCCTGGCTTTTATCACTAGAAAAATAAAATAAGAGGAAGTGGCAGTGGCAGTGGCAGTGACAGTTGACCTCTTATGGTATTGCTTTCTTTAATAAGGTTTGCTATCCTATCAAGAAGGATGAAATACATGTTTTACAAGCTGTCATATCCGTGCATTGTTATATTTTCAGTTTAACTGATTGAACACTTGGGCAAAGGATACTACTCATATGGTCACACCCAACGGTGTTATGCACATTTTCCCCTAACAGAAAAAGATAAAAAGTATAAAGTGAGCTTTCTTCTTTCTCACTGTTTATTAGGTATGTCTAGTTAAGTGGAACTCTCCCAATTAGATACAAGTTTCATCTTGAGGCTTCAAAACTAGAATTGTAATCAGAGCATCAATACTTATATCTACTATCCAACTTATTGTCATACATGAAGCATACTTCTCTACACTGAGAATAAAGAGAAGTCCTCACTACTTTTTGTTGGCGATTTTCCAGTTAATATGGTCCTTTTAGTTGGCGATCTAACATTCCATCTTTCTTATGCTAAGATTCAAACTACTATGCTGATTTTAATTTAATGAGTTGATGGATGAGTCTGCTTGTTTATACTGTTGTAATTGCTTGGCTGTGTAATATTGTAGACGTCTGGCAGGGGTAGATGTCTACCTTAAGCTGTGTTATTGTATATTTTTGGAGAACTTAAGCGAATTACAATGCAGGAAATTCTAAGAAAATGGAGCACAATGAGACAGGATGCCAAGCTCCTCCTGAAGCTCCCAAGCTTTGTGCCAACAACTGTGGATTCTTTGGAAGTCCAGCAACCATGAATCTGTGTTCCAAGTGCCACAAGGACTTGGTGTTGAAGCAAGAACAAGCTAAAGTCGTTGCGGCATCCATTGATAGCGCGGTGAATGGCAGCCCCTCTGAAAGTGGGAAGGGGCCTGTTGCTACTGCTGCTGTAGATGTACAAGCTGGCCCCGCAGATGTGATGCTTATCTCAACACAGGCATCCTCTACTCCATCTTTGAACATTAAGAGGGAGGAGAAGGTGAAAGAGACTCCTACCAGGTGCGGCACTTGCAGGAAACGTGTTGGTCTAACTGGGTTCAGTTGCCGATGTGGAGATCTCTTTTGTGCAGTTCATCGGTACTCTGATAAACACAACTGCCCTCATGATTACCGGACTGCTGCTCAGGATGCAATAGCCAAAGCCAACCCGGTTGTCAAGGCAGAAAAGCTGGATAAAATCTGATAAAAGTCTTGGAATTACAAGGTTTAATCTTTTTATGTATTGCACACATGCTCTCATTTCTCTACCCATGTGGCTTCTGCTTCT

>MdSAP10

ACCTTTTCCAAAACCGTTATTAGCACCTCAGAAAAGGGACAAAAGAGGAATTTCAGGACAACGGAGGTAAGACCTCCCGCCAATAGGAAACCGCCACGTCGGCCTCATAATAGAAACGGTCGTTGTTAAATTGCACGGGAGCGTCACACAGGAAAAATAGCGAAACACGACTTTCTCTCTCTCTGGCTGGCTCTCGCTTTCTCTCTCCTCGGGCGATCCAAAATCTGCTAGGGTTAGGGTTTTGAAAATCCCCTAATTTTGATTACTGCTTGATTTCTTTGTGTTTGGTTCAATTTTTCTGCTTTTGATCGAGGGCTTCCTTTGATCTTCGTATTCGGTTACTGACTCTCTGATTGTAATTCTTGGTTTTTTGGTGAACAGGGACTTAGTGTCCGATGCGATTCTGAGCTCGTGAATCGGTAGGTTCAATTTCAAGAGATTTGTTTTAACAGGGTAGGGTTTCTGTGCATGTATATATATGTGTGTGTGTATGTGCTTATATAGCTGACCAAATTTGGGGGGTTTTTTTGTTTGTTGATTTTTAGTGTTTTTAAGGTTCTCTGCAAATTTGCAATTTTGGATGTATGCATGTGTATATGTGTGTTTGTATAGCTGACCAAGTTTGGGTTTTTGTAGATTTGAAGTGTTTTTGAGGGGTTCTCTGTAAATTTGGGATTTGGGGATTTTTGTGTTGATTACCCAGTTTTTGTTGATTCCAGGTGTTTTCGATGGGTTTTCTGCAAATTTGCAATTTGGGGTGTGGATTATTTATATGGATGATTGATTAGTGAAAGTTTGCGAGTCAAAGGTCCGGTATTATGTAGATTGTAATTGCTAATTGGCCATAAATCTGAGGTTGAGTTTGAACTTTGGCACAAATCAGGTGGTCTATAATGATTAGATTTTATTTTTTGTATGTAATTGCTATCAGTTGGTTGATAGGTGTTTATGTAGCTTCACTACTATACTTGTTGGCATAACGAATGTCCGTTCGCAGATTATAATAAAGATTTCGCTTCTGATTTTTATAGCACAATTAGCGGTTTTCTATTTTTATGTCTTATTGAATTTACAAGCATATGTTCATATGATGCAAATTCAATAAGCCTTTTACAGGTTTAACATCTTTGGATGTTGTTAGTGATGTTATACAGGTTTTGTGGATTGAAATCTAAACGTTTTCTGTTTAAAGATGAGTTGTTGTGTGCAAATTATAAAGATAATGAAATACCCTGACTTCACGTTCTTCGATATTAAGTAACATGGAATCGTATGCAGTCTTAACAACTTTTTCCTTTCAAACAAAAAATTAGTATTTTCATTGCTTATATACCAGCATTAAATTGCACCACGAGCATTATTTTCATTATAAGTGGCGTGTTTGCTGAGTTTTTCATGTGTTTGTTGTTTTGGTTGCATGTGTGTGGCGGACTTGTAAGTTTTCACTCTTAATAAGCTGCACCCCATTAATTGTTGGTTTTCGCAGGTAAGTTGAAAAAATGGAGCACGAGGAGACTGGATGCCAAGCAGCCCCTGAAGGTCCTATTTTATGTGTAAACAACTGTGGGTTTTTTGGAAGCGCGGCTACTATGAATATGTGTTCCAAGTGTCACAAGGATATGATGTTGAAACAGGAGCAGGCGAAGCTTGCTGCATCATCCTTTGGAAGCATTGTCAACGGAACATCAAGCATCGATGCAAATGAGCCTGTTGTTGCGGCTGCTACTGTGGATGTTCAACCCCATCCAGTGGAGCCAAAAACTCTCTCATCGCAACCATCATTTTCCTTTGGTTCAGGGTCGTCTGGTGAGCCAAGGCCGGAGGGCCCAAAACGTTGCAACACTTGCAACAAGCGGGTTGGATTAACAGGGTTCAATTGTCGGTGTGGTCACCAATTTTGTGCAGTACATCGTTATTCAGACAAACATGACTGCCCTTACGATTATCGCACTGCTGGACGCGATGCTATTGCGAAAGCCAACCCGGTCGTAAAAGCTGAGAAGCTCGATAAAATCTAAAGCCTGATGCAGTGAAGTTTCATTTTCGAAATTGATGCTTGTTTTCATCCACGAGATGGCTTCAGAGTTTGCAGCGGTATCTTAGCACTTGCCGTTTTATAATGTTATACCTACTGGGGTGCCAAGGCGATCTAGGCATCTCTGCACTCTGAAGAACTCTGTATTTGTTGTGATTGGGAGAACTTTATGTGTTGCCTCGTGTCTTTCTAAGTTGTAGTCTGTGCCGGTTTGGTGGACATGGTTCGGGTTAATCTACGTAATGCGTCTACGCGCTGTTTATATCGCATTTCTTTCTTCTATTACATGTCAGTGGATCTTGTATTGTAAAGTATTGATGTGGTTTCCATGCATAAATCTATTTACCCTTCCTTTCTGGTCCATCGTGTTGTCATGCATGTTGTAAATATTCAGGGCCAGTTTGGTATCTTACTTGAAAATTACATTTCTTGAA

>MdSAP12

TTATTAATTAATCCTCCGGTTTCTCAAAATAATTTAACGGAAAAGCGTTTGAGATTTCGTCAATTTTTTTTGATTATTTTTTACTCTCGATCGCAGATCAGTTTTCCGAAATCAATTCTCTTCGGCGAAGACGACGACGATCTGATCTAATCGAATCCGATCGGCGAATTTAAGAAAAACAAGATCTTGAGGCGAAATTGTAAGCAGACATGGCGGAAGAGCACAGATGCGAAGCCCCAGAAGGCCACCACCTCTGCGCAAACAACTGCGGATTCTTCGGCAGCCCGGCCACCATGAACCTCTGCTCCAAATGCTACAGAGACTTCTGCCTCAAGGAGCAGCAGCAGGCCTCGATCAAATCCACCGTCGAAGCCTCCCTCTCCGCCTCCGCTGCCGATGCCGCTTCTTCCTCTCTCTCCGCTCCTTCTTCCCCTCCTTCAACATCCTTGCCTGCCTCTCCGGCAGCGATCGAGACTCTCTGCCAACCTCCGCCCCCGGCCTTGACTTTGCCGGAGGTAGCCGGAGATATAATCGGTGAGCCCGCTGAAGTTGTTCGGGCTCCTGAGGTGGCTACGGTGGTGTCGCAGCCGAACCGGTGCACCGTTTGCAGGAAACGGGTCGGGTTGACCGGGTTCAAGTGCAGGTGCGGGACCACGTTCTGCGGGGTTCACAGGTACCCCGAGAAGCACGCGTGCTCGTTCGATTTCAAGACCCTCGGGAGGGAGGAGATCGCCAGGAGCAACCCGTTGGTCATAGCCGAGAAGCTCGAGAAGATTTGATCCGGTCCGTCGGATCTTAACACCTTTGTCAAAATTCAAATCGCTGATCGGCGGGTTTCAATCAATCAGG

>MdSAP14

GTAAAGAGGTTAGTGGCCCTGAATTGGCAAGGCATCCCAGACCATGAGAGGCAGCTTTTGTCCAAATGGAGGACAATTTAAAGGCAAAGAAAGCAAAATGCTTTTGAAGCATTCTTTTTATATAATGGCTTTTATGGCAAATCATGAGAATCACTATATTTGTATAAGATACGGGTTTATATCATCAAAATGACAAGAAGTTTTTTGCATGCGGGGATGAATCTAAATTTTTAATTTTTCTTCCTTAATATTTGTACCATCGCCATCGAGTGGTTCATAAAATTATTAAATAATTTTATACATAAGATATAATATATTTTTGTAAAGCTTACATAAAACTCGTAACGCAAAAGAAATTAAAATCCGCAACCAATAGAGGCAATTGGACGGCGGCATGCTGCTAATATGCTGCATGTGGGTAAAACGGCGGTCCCACCGAACCTTAAAATAAGCTTTGTCAGGTTTGACCTTTTCTCTATAATGACGATTACCACGTCAGAACCAAATCTAGAACAATGAATCCGGATTCTAGTCGGATCCTCTATGTGAGGATTATGAGAATTCATCAATTATGTCTGTTCATCGTATATTGTGCGGTCAGAAATTATTTTAAATATTTTTATTTAAAATTAAACACAAACAGTATTGATAAAAACTGATATAACAGAGAAGATTCACAAATCTTCATGATCCTCACCAAAAGGATCCGGAGAGGATCATGTTGGTAGAACAACTAATCCAATACCAAACAAACATTAGGTGCAAGAGTATACAAAGAGTTTTACTTTTGAATCTTTTTAGCATTTTTTGGTATTCCTATTCACTCAAAGCAATAGGTCTTAACTCGATTTTTGTCAAAAGCGAGTTCCAACAAAATTATTAGGATGATGTTGTTGACACACCCAACTTCACATCTCACACACTTTCATTAATTTTATAGTGTTGAATCGAATGAATTGAACAAAATTAATAAACAAAAAATAATAAGAGTATGAGATATTAAAAAAAGTGTATTGATATCACTACTCAATTAGTGAGAAAAGTCTTTTGTGAAATTTAGCGCATTTTCCGCACTTTATTATTAATAGTATCGTATATATTAAAAAATAAAAAATAAAGAAAAATCAATTGTTCCTTTTCTCACTTTAATTTGTTTAAGCAAAAAGGTAATAATCAAAGAAAAGCACGAAGGGCATTTCGGTAAAAGTGAATGGCAAAATTGTTTTATACGCGTCTCTCTGAGGCCCCATATTTCCCCCATTACCGACCCTTCAGTCCTTCATAATATCCCCCTCCCCATTTCTCTGCTCCCATCGCCCGTCCAAACCTCTCCTCTCCTCTCTCTCTCTAAAAACCATAACTTTTTCTTCCTTTCTCTCTCTAGAAAAACCACACCCTCCATTTTTCTTTCTATCCATCTATCTATATCTCTGAAAAAACAGGAGAAAAAACAAAGAGAGGAGAGCGATCGATCGATCGATCAGAATTTGAGTGAAAAAAATGGCACAGAGAGCGGAGAAGGAAGAGACGGAGTTCAAGGTCCCCGAAACTCTAACGCACTGCGTCAACAACTGCGGCGTCACCGGTAATCCCTCCACCAACAACATGTGCCAGAAGTGCTTCAACGCCGCATCGGCCGCCGCCACCACCTCATCGTCGTCTTCGTCCGCGGCGATTTTGAAGTTTTCTGCAGAGAAAAGTCCGAGATCTACCTCATCCTTCAGCTTCGAGGCCCCGGTCGAGACCTTCCGGAAGACGACGGCGTCGGAGATCGCGAGATCGGACGAATCGCCGAATAGGCGCGTGGTCAATCGGTGCTCCGGATGCCGGAGAAAGGTCGGGTTGACCGGATTCCGGTGCCGGTGCGGCGAGCTCTTCTGCTCCGAGCACCGGTACTCCGACCGCCATGTGTGCAGCTACGACTACAAGGCCGCCGGTCGCGAGGCCATCGCGAGAGAAAACCCCGTGGTCAAGGCTGCGAAGATCGTCCGGCTCTGACCGGTTTGACAATTATACATCGATTGAATTTGTAGAAATGGAAACGACTGAAAAAGTTTCGGAATTTCCTCGAGAAAATTTGAATAGGTATTTGTGGATCGATCGGTTCTCATGGCGCGTCTCTCTCCCGGAGATCATCAGCAATCGTTTAGACGAAAAGCAAAACGGAATTTAGAGATGGAGATAGATCGCTTCGATAATCAAATTCTCTCTGAATTTTTTTTTTAATTTTAATTATATTTTTGTGCTCTTATTTTTATTTTTTGTCGTGGATTGGAAATGATAAAAATTATTGTGATGGTTTTACGATATTCGATGTGTTTGTTGAGAGTTCA

>MdSAP15

ATGCGCTTTACTGTTTTTTCAGTTGAACTTATTGTACACTTGAAAGAAAAAGAGACCTTTTTTTTTCTTCTCCCTATCGTTTAATTGATATTTTTAATTAAAAAAAATCTCACAATTATATATTTGTTTGCTGTTTAGGCTTCAGAACTAGAATTGAAATTAGCTTCAATACTCGTCTCAACGGTATCCAACTATTGTCACGCATGAAGCTTACCTCTGTGCACTGTGACTAAATGAAAGTCCCAAGTGCAGTAAGCTAGAAGGTTATGAACTATAAAATGATTGCAAATGTTAGATGAAGGCAGCAAAACAGGAGTGGGAAAAAAAATAAGGCAAAAAGAAAGCAAAGGAGAAAATGACCAGTAGCTGGAACATAAGACATTTTGTTGTGTCATCTGAGGTTGTTTATTAGATGTTGGTGATGGTGGTTGCGAGGTTAGTCATTGTCAAGCGTGAAATGAAGAAGGGGTCGGTTTTGTTCATGAAATGAACTAAAATGGTGAAATATATTCTGCGGGTCTTGGCTTCACGTGAATGGTGTAGATTTCAGGTCATGTATATTTTTATGTTCGCCCATGAGCGGTAGGTCTCAGGTTCGAGACTTGGGAGCAGCCTCTCCATAAAATGGGGGTAAGGCTAGCCGACATTCACCTCTCCCAGACCCTGCGTAAAGCGGGAGCCTTGTGCACTGGGTACGACCTTTTTTACTGATAGCTGTAGTAATGGTGTTCTCTACTTTCTGCTTCCCCTTTATAGAACAGATTGTCTCATAAAACAATCAATTGTCTATCATCAGTGTGGTTTCACTGTCATTCGGGACGGCACCAGAAATGTGTATGCACACTTATGTGGGACTGTCCTCACTTTCCTTTGTATGTGTATTAAATATTGGGAATTTGCTGCTACGGTTCCATGTAACTCATTTTTCTTGGTTTTTTTTTTTTGGTCATCATAGTGCTGCTATTATTTTCTTGTTTCTCCCTCTTTTTTTATGCCAGGAAAGTATCCCATTAGAAGTAAAAGGCCTTTAAGCCAACTGTGAAATCAAAAGAATCAAAACTAAACTATTGTGCTTCTGTTTCCAAGTGAAACCTTAATTAGTTATATTCCCTATGTAATTATTTTTATTATTCTTTAAACTGAGGAAACAAGTGTGAATAACTAAATGTGAGTATTCATTCAGAGCCATCGCCACATTCTGAGTTGAGATCTTAGGATCATGTTAAGGTGGCAGTGCCTTGGTTAAAGTATTGGTCAAAGAGTCATTTCCTGTTAATATGGTCCTTCTGATTGGCGATCATCTTTCTTGTGCTAAAGATTCAAACTGCTACGCTGATTTTAATTTAACGAGTTGATGGTGGAGTCGGCTTGTTTATATTGTGTAATTGCTTGGCTGTGTAGTATTGTAGATATCTGGTAGGGGGAGATGTCTACCTAGAGCTGTGTTATTGTATAATTTTGAAGGACTTAGCGAATTACAATGCAGGAAATTATAAGAAAATGGAGCACAACGAGACAGGATGCCAAGCTCCTCCTGAAGCTCCCAAGCTTTGTGCCAACAACTGTGGCTTCTTCGGAAGTCCCGCAACCATGAATTTGTGTTCCAAGTGCCACAAGGACTTGGTGTTGAAGCAAGAACAAGCTAAAGTCGTTGCAGCATCCATTGATAGTGTGGTGAATGGCAGTCCCACTGAAAGTAGCAAGGGGCCTGTTGCTACTGCTGCTGTAGATGTACAAGCTGGTTCAGCAGATGTGATGCTTATCTCAACACAGGCTTCCTCTACTTCGTTGAACATTAAGAGTGAGGAGAAGGTGAAAGAGACTCCTACGAGGTGCGGCACTTGCAGGAAACGTGTTGGTCTGACAGGGTTCAGTTGCCGTTGTGGAGATATCTTTTGTGCAGTTCATCGGTACTCTGATAAACACAAGTGCCCCTATGATTACCGGACTGCTGCTCAGGATGCAATAGCCAAAGCCAACCCGGTTGTCAAGGCAGACAAGCTGGATAAAATCTGATAGAATCTCGGAATAACGAGGGTTTAATCCTTTTATCTATTGCACACATGCTCTCATTTCTACCCATGTGGCTTCTGCTTCTGTTTAAGTTGATCTGTGTTGTGTTAGGTCTCGTGTGATAGATGGTTGGAAGCTGTTGTGGGCATTTTCATATGCCAGGAACGATTGTGTGTAAT

>MdSAP16

CACGAGGAGAGCACTAAAATGGAAGGGCAGACAATCCAAGTCCAAGGTAAAATGGTGGTAGTAGAGATGACAATCATAATGGCGCAGACAACAATGTGGTTATTACCACATGATCTGGATCAATTTGCCTCTTCAAAACCAAAGCCCAACCCAACACGACTATGTGTCCGTCCAGCCCAGACCAAACTCCAAATCCCCGAACAGTTCACAGTCCGACTGGGACCTGTACAGTGAAATGGCAACACCGCAATTATGTGGTCGTCGAGGGGCGGTTACATCGCTACCACCGACTTAGTGGAAAGCGTTCGTTGGCAAAACGGAAAGGTGAAATAAAGAGCCCCCACGAGAAGCCTCCAAACCAAAAAACCCAGCACTACCAATTATCTCTTACCCCTTTTTATTTTTTATTTATTTTTTTATGTACCGCCCTACATATTTATTTTTATTTTTCCAATAATTAATATCAAAGCAATAATCTCCTCGATCAGGGTATGAAGCTCATCCCCCTCCCTCTCTGCTTTTTTTTTATTTTTTATATTTTATTTTCCAGTATTTGTTTGATTTTGTTATTAATTTATTGTTTTTTGAAATTTGAAATTTTGAAACGAGGAGCAGGAATTGCCTGGTTGGATTTTCGACTCGGATCGGCCGGAACCAAACAGGTTAGAGTTTGATTTTGTGTATGAATTTTGGGATTGGGAATTGTATGGGTCAATTTTTTGTACTTGTAAATGCAAGCTCCCGAATTCCGTAGTGTTTTCAATTTCTTTGGTCAATTAAGTGTGGAGGGTTTCATTCGTCTGGAATTGAAAACGTTTACGGTTGAAAGTTTGGATTTTTTGTTGTCTGACTGGGATTGTTCGTTTATTATCGGAGTTTTTGAAGATTTTGGCATGTATAGAGGAATTTGTAGGAGTTTATGGGTTATTTGTATAGGTTTGAACTGAGCTTGTTTGGATTATATCGGATTTGGGTCAAGTTCACGACGTTATAGGGAAAATTTTGATGTTTTGTTGGTTTTCTGCATATGTTATTAAGTGGCTTGAATTGTAAACGTTGGCCTGGACAGTTCACCTCGGGGCATTCGATTTGTATGACAGAGCAAGTGAAATTGAAGTTTTACCCATGTTGAAAATCTGAAAAGTTTGGCGGAAGGAGATAATTAGCCCGAATCATATACTACTTGCATGATTCGATGTGTTTAAGTCCATATGAGTTCTATTTTCGTCTTTGGTTATTTGGTACTTGTGGTATGGCATCTGTAAAATTGCTTTTATGTAATTCACTCTGCTGGGCGTTTGGTTATTTGGTAGCTCCAAGTTTGATTATATGTTTGTAGTTATAGAATTTCGCCTCTATTTTGCATGTTTATGGTGGTTTGATATTTTTGCAGTGGGTGAATAGGCCATTGGGTTAAGACTTGTTTTATGATGTTTAAACGGCGAGGTTTTCTTGGTGCAGATAAGTAGAAAAGATGGAATCTCATGATGAAACTGGATGCCAAGCTCCAGACCGCCCTATCCTTTGTGTTAATAACTGTGGATTCTTTGGAAGGGCCGCGACAATGAACATGTGTTCCAAGTGTTACAAGGACACGCTCTTAAAGCAGGAGCAAGCCAATTTGGCTGCATCATCCATTGACAGCATTGTGAATGGCGGCGGCAGCAGCAGCAGCAGCAACATCTTCATTGACCCAGTTGTTGCAGGTGTTGTCGATGTGCAAGCTGTACGAGTGGAGACAAGTGTTGTCTCAACAGAAGCATATATTGAATCATCCCCAAGCATGAAGATTGAGATGAAAGAAAATAAGGGACCAAGCAGATGCACTACTTGCCGAAAGCGCGTTGGTTTAACTGGCTTCAATTGCAAATGTGGAAACACCTTCTGTGCAAGTCATCGTTATTCTGATAAACATGACTGTCCTTTTGATTATAGGACTGCTGGTCAGGATGCTATTGCAAAAGCCAATCCTATCGTGAAGGCAGACAAACTTGACAAAATCTAGGAGTCGCGGGCGGAAGTGAAGATCAATCTATTTGAGGATGATGATTCTCTTTCTTGGCTTCTTGTCTGCATAGGTCTCCTAATGGTAAAATTGCTTAAGGTCATGATAATGAGGATATCATTTAAGTGAAGTCTATTCCTTGTTGGTGAAATATTTCTAAGCTGGCTCCACTGTCTTAAGTCGTTATTATTGTTGGTTTGATGGATGTCTTTGGAGTTATTTTAGTGTGTTCTATGCTTGGTATGCATTTCTTTATCGCAGTACAATTATGCAATGTAATGCAATGCAACTAAAATTTCCTTAAATTGTTTGTTCCACATTTTCACAATGTATATATGTGCTCAGTTCAGATTTGAAA

>MdSAP19

ACCTTTCTTTTGAGAAGTTTGTTTGATGCAAACTTCTTTTATATTTTTTTTATATTCTTTTATTTTATATATATATATATATATATATATATATTTTTTTTTTTGTTCAACACCGACAATTAAAAATTAAAAAGAACGTGTAAAGAAGTAAGAAAAGATGTGTGAATAGCACTACCCCGATAATAATTAATAACAACACGGGTTGGTGTTCGAGTGCATTTTTGCTCACCATCATTTAATGTGGTGTATACTCACCATCTTATTTATCATCGTTAGATGAGTTTGAATTTTGTGTTTATCCACTACACAAATCTCAAAATTCAAATTCATCTAACAGTAATTAACAGGACGGTGATAAAAAATGCAACCAGACGTAGACTTGTACCTTTCAATGGCCAGCAACAGCCGGATTTAAAAAAATTAAAACCGGGGACCCACTCCAACACCCGCACGAAGGGGAGGCCCACATGAAAACGATACGAGCGGCCCCGGATCGGCAAACCGAGCCGGTCCAAATTGGAGAGGCGAATTAAATATCCAAAATCCCTGCCGTGGCCGAAACGACTTCCAGAAGATGACCAATTTAATTATTTCAAAATTTGAACAGCCTCAATAAAATCCCCCTCTTCTGTCCATTTTACGCGCATTTTAATAAGAGAAAAAAAAATTAAAAAAAAGAGAGAGAGAAAAAGAGAGAGAAAAAGGAGAGGAAAGCTCTCTGGAATCTTGTTTCCCTTGAGGACCAGGAATCCCAATCGCCAAGGGACCTCCTGGTTTTTCTTTTTCCCTCCTTTCGGTTTGTTCTTCCCAGAAATTGTGATTCTGCGATCATCTTCTTAATTCGATTTCCAAACAGGTATTTTGGTTGTGTTGTTATTTGGATCTGTTTGGTTTGGGGCAAAATTTGTCTGATTTGTCGAGATTTTGATCGGATTCGCATATATGGTTCCGGAAATTTTCGAATAATTTGTTTTGGAATTGCAGAAAATTGAGATCGTAATGGCGGAAGAGCATCGTTGCCAGGCACAGCAGCTATGCGTCAACAACTGCGGTTTCTTCGGAAGCCCGACGACGCAGAACTTGTGTTCCAAATGCTACCGTGACTTGCAGCTCAAGGAACAACAGGCGGTCGCTCTCAACCAAACTCTCATCTCCTCTTCCTCTTTCGCTTCTCCTTCCTCCTCCTCCTCTCCCTCTCCCTCTCGTCCGTTTTCGTCCCCGCTGATCTCCGTCTCACCCGCGCACAAGGCACGGGTGGAGCGCGTGGTCGAAGCCAAGGAGGAAGAGGAGAAGGAGGCCGCGCCGTCGGCGGGGGCGCAGGCGAACAGGTGCACGACGTGCCGACGGCGCGTGGGGTTGACGGGGTTTAAGTGCAGGTGCGGGATGACGTTCTGTGGGACCCACAGGTACCCGGAGCAGCACGCGTGCGGGTTCGATTTCAGAGGGATGGGGAAGGAGCAGATCGCCAAGGCCAACCCGGTTGTGAAGGCGGAGAAGCTGCATAAGATTTGAGAGGAGGCGGCCGTCAATATCATTATTTCCAGGGCTGCAATGGTCATTTTACCTTTCGTGGTTTGGGAAATTGGGGTAGGGGATGGTTGGCTGATGGTGTGGGTGGGATAATGTTATTGCTTGGTCTTTGTAAAAATGTAATCATAATTTAATGGTTTCCGCTTCTTTTTTTCCCCCTTTTTTCAAAAGAAAAAGAAAACAATTTGTCCATAA

>MdSAP21

ATGGATTCTAGTTTGATTTGGGCATCAAAACAAAGAGAATCACTTATTGGTGGGCTGCGAAGTGGATGGTAGCTCAGGTCCAGTAACTTTTTTGGGCCCATACCCATCTAGTAACTTTCTCACTCGAACTTTCTTCGCTTCCATTCTCATTATGCATCGTTACTTTGATGTTTGGGGACTTGGATCCTCTCTTGAGCCCAAGGAGAGGATCCTCCTGACCAAATGATTTGGGCCGTAGGATTTCATCCAACGGGTATAAATAGGGGGTCTCTTTAAAGTTATAATAATTATATCCGTTGAATTTTCATCCAACGATCCAAGTTCTTTGGTTAGGAGGATCCTCTCCTTGGGCTTAGGAGAGGATCCAAGTCCGATGTTTGGAACCATTTACTTATGACATCACCCTTTGAAGGCCAAGTCTGCAAAGAATCAACGAAATTAGAACTCGTTTAATCGTTCGATGAATGCTGTTAAATTTAAGTGTTTAAGTGCGAAACATAGTTTGTGGTAAAGAGATCCATTCATTGGTAACAAAAAGAAAAAACGTGAACGGTGATTGGATTGATGATAAAATAGAATCTGAGGTCTCGAACAGTGATTCGATTGATGATAAAGAAAGAGAATTCTTAGTTCAATTCTCCACCTTAACGACCTAAAAAAGGATTGATCAAATTCTATTGAGTCATACTCATAAACAAAACGATATTATCAACACTAACGAGGCAGGGGATTATGTTAAGTTTTATAATGAGCTAGCTATAATATGGTTCAAATTCGTCTTTGGCGAAAATCGACCTCTCACTTTACAAGTGAAGAGGGAATACCACTAAACCATAATACTAATTAGCTTGTCTATTTATTAGATAACCGTTACAAGATTTAATTAATTTTTTGCTCAGATAATTTTTAAATAGTTTACTAGACATCTTGATCATCGAACAACATTTCACGATGAGTTGGAGAGCTGAAGTGAATTGATCGAAAAATACGTTGGGGGGATGTATGTCCTAATCCTCTTTGTAAGGATTCCAAAGATCCTCCAATTACATCCGTTCATCGTAAATTGTACAGTCAATTTTTGTCAAGTACTGTGTATTTTCAATTTAGATAAAAAAAATTTACAATGATTTCTGATCGCACAATATATAATGAACAAATGTAATTTAAAAATTTTCGAAATCTTTACAAAAAAGATCCGGGGAGAATCCTCACCCTTTTGCAAGGATGGTCCAACAGGTGGGTGGCGTGGGTCCTTACGTTCCGTGTCAGCGTTTGCGATGACGCGTACAAACTACAAATTGTAGGAGCGAAGGAAGGAGCATCGTGCGACACCGTGTGGAATCCGAGTCAAACCTGGCTTTTGGCTTTAGCTTTTCCCGCAAGCCAACCCCTGAACCGACCCCTCTCCCTCTATAAATAAAATTTAAATTCCCCCCTTTTGCTTACGAATTGTGTTTCTTTTCAAAATTTAACAAACTGAAAAATCAAATTACGATCAGGATGATGGGAGGAACAGAAGCTTTTCCGGATTTGGGTCGACACTGCCAGCTCTCCGATTGCCACCAACTCGATTTCCTCCCCTTCCAATGCGACGGTTGCCGTAAGGTTTTCTGCGTTGAGCATCGGTCTTACAAGGGCCACGAGTGCCCCAAATCGGACCACAACAGCAGAAAGGTGGTGGTTTGCGAAATCTGCTCCGCCTCCGTAGAAACCACAGGCTGCGACGGCGAACAAGACCACAAATTGCTGTTGCAGAAGCACGCCAAGTCCGGGAATTGCGACCCCAAAAACAAGAAGAAGCCCACCTGTCCCGTTCGCCGGTGCAAGGAGACTCTCACCTTTTCTAATACCAGCACCTGCAAAACCTGCCAGATCAAGGTCTGCCTCAAGCACCGGTTCCCGGCCGACCATGTTTGCAGGAAGCAAACGGCAGCTCAGCCGTCGTTGCTGGCGGGTAATGGCGTCAGCTGGAACGACAGGTTCATGGCTGCTTTTGCTTCCAGGAAAGGGAAAGAATGCGGGAAGAGTGAGCGGGATTCCAAGTCTTCTGCTTCGAGTGGGCCATCTGTTCGAGCGTATTGAGAGATTGTTCATATGCTTCTGCTGGGTTTGTTTATCATTCCGCAGTTTCATGTTTCTTCGAAACAGCGTCGTTTCGTTGTAATTCTTTTAAATGGTCAAGATTTTAAGATCCTTCATCATTAATCTACAAATTACGATTCATACTTTGATCCAATTGTAGTACATTAAAGAACGTTGGAGTAATCCTCTGTAACTGCAAATCACAGTGTTACGAGTATTTTATGTACTTGCAAATCACAGTGTTACGAGTATTTTATGTACAAAAGTACACCTACAAAACGAGAAA

>MdSAP23

AACCATATTTCCATCACATTGAATAATAGTTTTTACTTGGGAAAGAATAAAATCTCAACATTACCATTGTGATTAACCGACCACTACACCTAACCCTTACCAGCATATTTCCCAACCTTTTCGAAGAAAAGGGACAAAAAGGGCAATTCATGAGAGCCAATGGGATCCCGCCACGTCAGACTCATGGTAGAAATAGTCGTTATTAAATTGCACGAGAGGGTCGAACAGGAAAACGAAAAACAGAGAAACACGACTCTCTCTCTCTCTCTCTGGCTGGCTCTCGCTTTCTCTCTCCTCGGGCGATCCAAAATTTGCTAGGTTAGGGTTTTTGAATATCCCCTAATTTCAATTACTGTTGATTTCTTTGAATTTGGTTTCAAGTTGTCTGCTTTAGATAGAGGGCTTTCTTTGATCTTTGTATTCGATTGCTGCCTCTGATTTTAATTCTTGGTTTTTTTGGATGAACAGGGGCTTCGTGTTCGATGCGATTCTGAGCTCGGGAAATCGGTTTCAGAGGTTCAATTTCAAGAGGTTTTCTTTTAACAGGGTACGATTTCGGTGTATGTGTATGTATATTTATACATGTGCTTGTATAGCTGACCAAGTTCGGGTTTTTTTTGTAGATTTTAGGTGTTTTTTAGGGGTTGTCTGCAAATTTGGGATTTGGAATTTTTGTGTTGATTTAACAGGGTACGATTCCAGGTGTTTTCAGGGGTTATCTGCAAATTTGCAAATTTGGGTTTTTGTGTGGATTATTATTTGAATGATTATTTAGTTCAAATTTGAGAGTCAAGGGTCAGGTATTGGTTAGTTTGTGAAGTTATTGTAATTGCTAGTTGGGGTTGAATTTGAACTTTGGCACAAATGAAGAGGTCTATCATAATTAGATTTCATTTTTATTTTGTATTTGAATTTATTCTTTTGTATGTATTTGCTCTCAGTTCGTTGATAGGTGTTTTTGTGTGGCTTGACTACTAATACTTGTCGTCACGACGAATGTGCATTCGGGTATTGTAATAAAAGATTTTGCTTCTGATTTTTAGCAGGTTTCTGTTTTCTTTTATCTTATTGAATTTGCAAGCATATGTCCTTGTGACACAAATTCAAAAAGAATTTTTACTGGTTTAACATCTTTGGATGTTATTAATTTCCGGATTGAAATCTAAACGGTTTCTGTTCAAAGATTAGTTGTGTGGAAATTATAAAGACAATGCAATACCCTGACTTCACATTCCTTGATATTATGTCACATGGAATCGTATAGTCTTAACAAGTTGTGCCTTTCCAACAAGAATTTAGTATTTACATTGCTTATATACAACCAGCATTAAATTATACTGCGAGCATTATTTTAATTATACATGGTGTTCGGTAAGTTATTGCTTGTGTTTGTTCTTTTGATTTCGTGTATAGGGGGCTTGTGAATTTCCGCTTTTACTGAGCTGTCCCCCATTAAATTGATGGTTTTCAGGTGTTCTCTTTGCAGGTAAGCTGAGTAGATGGAGCACGAGGAGACTGGATGTCAAGCTGCCCCTGAAGGTCCTATTTTGTGCGTAAACAATTGTGGGTTTTTCGGAAGTGCGGCTACTATGAATATGTGTTCCAAGTGTCACAAGGACATGATGTTGAAACAGGAGCAGGCGAAGCTTGCTGCATCATCCTTTGGAAGCATTGTCAACGGAACATCAAGCATCAATGCAAATGAGCCTGTTATTGCTGCTCCTCCTGTGGACATTCAATCTCAGCCAGTAGCACCACAAACTATCAGCTCACAACCATCATTTTCCTTTGGTTCTGGGTCATCTGGTGAGCCAAAGCCAGAGGGCCCGAAACGATGCAGCAGTTGCAACAAGCGGGTTGGATTAACAGGGTTCAATTGTCGGTGTGGTCACCTCTTTTGCGCAGTACATCGTTATTCAGACAAACATGACTGTCCTTACGATTATCGCACTGCTGGACGGGATGCTATTGCGAAAGCCAACCCAGTAGTAAAAGCTGAGAAGCTCGATAAAATCTAAGCCTGGTGCAGTGAAGTTTCATTTT

>MdSAP25

ACGTGGCAGGTAGAGTTTCAAAGTACGTCAATTAATTAGATGTGCTTACTGAGCTTTGAGTACAAAGTACATACAAAAAC

CAGCAAGGTGATATATATGCAGGGAGGGTCAGGAAGTGCAAGCAACATCAGATTTCAGAACATTGAAATGCATAATGTTA

CCAACCCCATACTTATAGACCAATATTACTGTGACAGAAAGATACCGTGCAAAGAACAGGTAATTTATAGAAATACCACG

AGACCAAACAAACTTCTATCGCTTAATTTCTTGATCGAAAATGACGTATCTATTGGTTCAATATTAATCACAGAGATCAG

CAGTTGAAGTCAAAAATGTGGTGTACAAAAACATCAAAGGAACAAGTGCTTCAGATGTTGCTATAAAATTCGATTGCAGC

AAGAGCTTCCCTTGTCTGGGGATTATATTGCAAGATATTAACCTCCAACACGAAGGAAGGAAAACAGCTAAAGCTCTGTG

CAACAATGTTAATGTGACTAGCATAGGGGTTGTTTGCCCGCTTTGTCCTAAATTAGAAGGGCAATATGAGACTTGCCAAT

GTCTATGAACACAGCTGTGTTGAGCTGGCTTAATATGTGTTGTTATTGTCCTGCATTTCATTATATGTATCAAAAGTTGG

ACGGTATTAGTATAATAATTAACATGCATTATCATAATGCATGGATCCATTTATTTGATACATACGTCGACTAAACCTGT

TGGAGGAGTATCGCGCAAGGATTAATTTAGATCTTCTTAGATTATGCTTTGATTAATTAGTTTCCTTATTGTAACTATGT

TATGACCTCCAAGGCATACACTTGTGTGTGTGTGTATATGTGTATATATAGCTATCAATAAAATTAATAATTCTATATTT

TAGAGCTAACTGTTGCACATACGGTACTTTTCAGAATATGTAACTCATATTTTATTCAAAACGATTTTGCATTGAGACTT

TTTTATGTATTACCCAGTACATCATATGTAATAATACAAGTAAAAATATATTTTTAGTATCTCTTTTCTTTATTATATAC

CATGTATACAAAATCATGGGCTAAGTACAGTGAAAATATCTCATTTACATCGATAAAGAGAGTACGCATATTTAGTTTTA

CTAAAAGCCCATGGGCCCAAACACACTGGCTTTCTCATTTATTCCAGCCCAAATCTCCAATTCCTTCTTTGTTTTTTTTT

TTTTTGTCATTTTTCCGTTTTTGTTCGTCCATTTGGTTTGCCAGTAAATTTCCCAGGGGGCAATTTGGACGACCGTCTTT

TTTCCCTTCGGAGAAAACTCGGGCGTGGACTTGGGATTTCCGCGGATTCGGTGTTTCTCTCTGTATCCGCGGAACCCAAA

ATCCAATCCAAACCTTCGGACAATCCGATTCCGGCGTCTCTCAACAAATCCCGGGACTACATAATCCCGCCGATTCTACA

TTTCCAGCACGTTTCGTTTATTGGCGGGGAGCTTCTAGTGTTGTACATACAAAATTTGAATGGGAACTCCGGAATTCCCAGATCTGGGTAGGCATTGCTACGTCGCCGAGTGCCAGCAGATCGATTTCTTGCCCTTCACCTGCGATAGCTGCCATCAGGTCCTCTCTCTCTATATATATACACRCATTTGTGAAATTATATACGTCCATATTATATAAATATCGAATGTGCATTGTGTTTGGAAATAGGGATTCTAGTTATATGGATCGAGATTATCCTCTGTTTGGTTGCTGAGAAAAGTAGAATTACCCAGAAARAAAAAAAGAWGGGAAATTTTCAAATTTTGGTGATTCGCGGGCCGGGGGGATTGCTTGAGAAAACGCAAAATGGGTGTGAATGCACATTGGATTGGATTATGAATTTGTATTTCTTGGAAAAGAAAAGGATGAAATTCCGGATTAAATTTATCTTATCGAAATTTGAATGATTGACAATTTTCTGTCAACATTGTCTGGAAGCTTACAATAAATTTCTGATGTTGGCGTTTGCTTGAGTTGAAACTGTCTAGCTTTATAGGGTGATATTTCATTCCTGCTCTGTATTTTCTTTTGCTATAAATGTTTGYTTCGGATTCATGTTAGATTGACGTGCATGGTGCAATGTTTGGATTTAACTGAATTCCAAGTACATTACTGAATATTTACTTATKTGATGTTAAATATCGAGGTGCTAAAGTTGTTTGCTGGGAATAAATTGAATGTTGTAGCTTCATGTTCTTAGATTACTCTAAGAAGTGGTATGTTTTKGAATTTGGAGAAAATAATGACGGTGGAAAGTGTTTGAAATTTACAAACTGATGGTAAAAGGGAAGTGAAATGTTTTGCACCCTCGTTCTTCCGGAATGGTGGTGTGATGCATCACGTAGATCATGCAGTTTTTGACCAAATTTAACCAAAGATAGTTAGGGGTTGGAATGTTTTCCATTTACTTCAATATTTTTAGGTCAGAAAAGCCTTATTTACTTAGGTTAATACAATAGCATAGTCTTCAATCTTTTTACCATCTTCCTAATTGTGGATTATCATTCACCTGCCTTATTTCTGCTTCTCAAGAATATTCAATGCTTCATACTCACACAAACCTGACATTATGCAGGTATTTTGTTTGGAGCATAGAAGCTACATTAAGCATAATTGTCCGAAAGGTGACCGAAAAAATGTCACTGTTGTCATCTGTCCCCTCTGTGCCAAAGGAGTTCATCTGATTCCCGATGAAGATCCAAACATTACTTGGGAGAGACATGTTAACACTGATTGCGACCCTTCTAATTATGAGAAAGCCACAAAGAAGAAAAAATGCCCTGTCCCTGGCTGCAAGGAGATCTTAACATTCTCCAACACAATCAAGTGCAGGGATTGCACGGTAGATCACTGTTTGAAGCACCGATTTGGACTTGATCACAAGTGTCCTGGACCCAAGAAACCAGAAGCAGGATTTCCCTTTTTGGGTTATTTAAGTAGGAGTAGGAAAGAAGTGTCAAAACCGAATCATGCTCCTGCTGCATCCTCCCCGAATTGGAGTAGCTTTCTTACTGCAGCTTCATCTTTTCGAGCCTCAGCTGAAGCAAGTGTGGCAAAACTGAGTAGCGAACTTAGCCAAAAGTGGCAGATAGCAAAGGATGGAACTGGGCAGAGTAGCAGCAGCAGCGGGAGTAGAAACGGGCAGGGTGAGGTGTGTCCTCAGTGTGGTGCTAAGTTTTCCTCAGTCACCACTCTAGTAGACCACGTAGAAAAAGTTCATGAGAAGGGTGGCAACCGAGCTGCTGCGGTGAAGAAGGTGACAATTGATGCCTGCCCTAAATGTAGTAAGGGATTTCGTGATCCAGTGGCACTTGTAGAGCACGTTGAGAGAGATCACGGCGGTACTTCAAGAGCATAGGTATCGTTTTTGGAGAAAGTATTTGTTATTTATAT

>MdSAP28

ACAGGTCACCGTGGTGACTCCGGTTGGTTTGGATATTGAGCTATGGAATTAACCATACAGGACCAACTGCAGGCTCTCCGGTTGATTCATTATGTCACCTGTTATATTGATGCATTCATAACCTGTTATTGACAGTTATGACATGGCATATTTTCTGGAATTGATAAAGATTCGTGATTTGAGTTATATGAAGAGTTATATGCATATTATGTTAATTTCTGGGAAAGTATATAGGTTTTACAGCGAGGGGTTAGAATTGTTTTTGATGAAATGTTTTCGAAAAGCTTTGGTTTTACTTACCCACTCAATTTTGTTTTGCGCCCCTCCAGGTTCTAGTTAGCAGCGTTGGTGGCCCACGAGGATCCCCACGGCGTACTGACAGACTACATAAATGTAGGACTCACCTGCGGGTGTTGTAATTTAGTTATAGTCCTACTGACTGCACCTAGTTGTTATGCTCTGAAATTGTGTGTTTCGCACTTAAACTTACTCTAGCACGTTACTTGGTTATTATTGCTAGTATGTGGTTTTTTGTTTATTTGTATTTCTCTTATCTTTTACTTCCGCGTTGCACTTTTGGTTACGTCACACTCATGTGACGACCAGCACGCCTTGATTCTAGGATCGGGGTGTGTCATTTGTGATGAGTGCAGGACCAACTACAACCATGCTCTGATACCAAACTAACACATTTCGGGTTGGGATGTGTCAGTTTGGCCCTATGTTTTTATTATATTCCAATACTTCATAATACATATTTCATTCTATTTTGTAGTTTATAATGAAATTATATATATTTTAAGTATAAACAGACACTTATTTACAGAAATATTATGGATTTACTTAACTTTGTCTAGTCCACCTAGGCGCCCGCCAAGACCCCGCCTAGCCGTCTAGGCACTAGGCTCCAGCATGCCGCCTAATTAACGCCTAACGTCTTTTAGAACCTTGCTCTCGACTCCACATCCATGTCCATTTTGATCACTATCCATGTCCATTTAATATTGTCCTTGAATAATCAATGGGTCTTTATTTTCTTTTTGTTCAAAAAAAAAAAACGCCAATAACTATTAACTTTTGTGGGAAAACCTTCATGAGTTTAAGCGGATAAAAAACTTTTTATAATTTCAATTGTCATGCTTTAATTAATTAATTTATTTTTTGTTAATCGATAAATTTTGTTAGATTAGATGTTAGATTAGTCACCGAAAGAGTTCAAAAAAATTCTAATTTTTGTGGGTCTATCAATCTTGTATTTTTGGAGGGATATATTATGGGGTCCCATTCCGTCGAGGAGGAAAGGAAAGAGGAGTCGTGCGAAACTAGAAACTTGCATCTCCTGCAATCCGCGTCAAACCTGGTTTTGGCTTCCGCCCCAAGCCAACCCCAGACCGACCGACCGACCTTGTATCTCCCTCTATATATTAACCTTCCAAGAGGGGCCGACTGCCAATCCAAGATCAGAGATTCCAAGGGAATTGAATTCAGATTACGAACAAGATGACGGGAGGAACAGAAGCTTTCCCGGATTTGGGAAGACACTGCCAGCTCTCCGATTGCCACCAGCTCGATTTCCTCCCCTTCCACTGCGACGGTTGTCATAGGGTACGTTGGGCTTCGTGGATTTGATACGGAAAATCAAGCGTTTTTGCATGTTTTGATTGCCTTGAATTTACGAATGTGCAGGTTTTCTGCGTTGAGCATCGGTCCTACAAGTCCCACGAGTGCCCGAAATCGGACCACAACAGCAGAAAGGTGGTGGTTTGCGAAATCTGCTCGACATCCATAGAAACCACCGGCCGCGACGGCGAACAGGACCAGAAATTGCTGTTGGAGAAGCACGCCAAGTCCGGAAATTGCGACCCCAGAAAGAAGAAGAAACCAACCTGCCCGGTTCGCCGGTGCAAGGAGATTCTCACGTTTTCGAATACCAGCACCTGCAAGACCTGCAAGATCAAGGTTTGCCTCAAGCACCGGTTCCCGGCCGACCATGTTTGCCAGAAGCAGACGGCGGCACAGCCGTCGTTGGTGGGGAAGCCTGTGAGCTGGAACGACAAGTTCATGGCTGCTTTTGCTTTGAGGGAAGGGAAAGAATGCGGGAAGAGCGCGCGGGATTCCAAGTCTTCTGCTTCGAGTGCGCCTTCTGTTCGAGCTTATTGAGAGATTGTTCATCTGCTTCCACAATTCGATGTTTGTCCGAAACGACATCGTTACGTTGTAATTCTTGGATTCAATTATAGTACACGAATAATCGTT

>MdSAP29

GTGCTTTTTGTTGGAACACAAAGTGAAAATATTGTTTATAATTTTTTTGTCAAATGATAAATTTTATTAAATTAAATGTTAAATTAATTATAAAAAAGAATTAAACAAACACCGTAAGGCAAGAATTCAATACTTTTTTACAACTATAGGAAAAAGCTATATTGATAACAATTATAAAATAACAAGAATTATATATTGACATCTCACAATATGTCTAATTACTATACTTTTTGAATCTACAGGGATGTATTCAATTGAAAATTTGAGATATTTAATGGATTTTTATGGAGATTAACAGATTTGTGATGATTCTATGTAAAATTTTGATTCAATTCTCTCGAAATCTCATGAGCAGATGTGAGATTTGTATATACTTAAAATACACTACAAAATCTCTCCAATTTCCTCTAATTCCTCAACTTTTCCAAATTCTTTAAATTCTAATTCTAATTGAATACACCTGGAATGTTATAAACTTTTTTAAAATTCTAATTGAATACACCCGGATTTCTAAGGATTTTAATAAACTATCTTAAAATCCTAATTGAATACACATTGAATTTAAGAGAATCAATTAAAATCCTGATTGAATACTAAAAGAATTAAAACCTCTTACAATCTGAATTGAATACACCTCCTACATTTACGTAATATAATTTAAATTAAATAATAACATCTCTGACGACATTATGATGGAGAAAATGGCCGATCAATCGTATTAATATTGATATTGGACGGTCTAATTCTGTCACGTTTCACCGTTCCTTCGCCCTTTTTTATTAAATTATTAATATTCCTCCCTCTGACCTTGTCAATAATTCTTCCGTCTTAAGATTCTTAATAACGTCTCTCTCTCCTCGTTCATAATCTTATCCCCACTCCTCCTCGCTCCGCTTCACAAAAAATCTCTCGATTCCTCCTCGCACAGGTACTAAAATCTCTCCTTCTCCCACAATTTTTTTTTTATATATTTTTTTATTTTTTTTGTTGGTGATTTTCTGTTTGGTAGCTGAGAAAATTAAGGAGGAAAAATGAAAGAAAACAGATGGGAGAATCCAGAATCCGCTTCGTTTGGTTCCGAAAAATTAGTTTCCAAAAACGCGGATCCTCCGGACGCTGATGGTTTTTTGTTATTAGCCGAAGGGCTAGCTGTTTTTTTTTTTTTTTTTTGAGTGTGGGAACCGGAAATTGGATTTTTCTTTTTCCTTCATTTGGTTTCTCAGCAACCAAACAGCTTATTAATTATTTAATCCTCCAGTTTTTCAAATTAATTTAACGGAAAACCGTTTGAGATTTTTCGTCAAATTTTTTATAATAAACCGTTTGAGATTTTTCGTCAAATTTGTTATAATATTTTTGTCCTCTCGATCGCAGATCAGTTTTCGGAAATCGATTCTCTTCGGCGGAGAAGACGACGATCTGAACTGATTGCATCCTATCGGCGAATTTGAGAAAAACAAGATCTTGAGGCGGAAATTGAATAATTGATTGACTGTAGACATGGCGGAAGAGCACAGATGCGAAGCCCCAGAAGGCCACCACCTCTGCGCAAACAACTGCGGGTTCTTNGGCAGCCCGGCCACCATGAACCTCTGCTCCAAATGCTACAGAGATTTCTGCCTCAAGGAGCAGCAGGAGGCCTCGATTAAATCCACCGTCGAAGCCTCCCTCTCCGCCTCCGCCGCCTCCGCCGCCGCCGCTTCTCCTCCTTGTTCTCCGCCTTCAACATCCTTGCCTTCTTCTGCGGCAGCGATCGAGACTCAATGTCAGCCTCCGCCTCCGGCGTTGACTTTGCCGGAGGTAGTCGGAGATATAATCAAAGATCCCGCCGGAGATCTCCGGGCTCGTGAGGTGGCTGAGGTGGTGTCGCAGCCAAACCGGTGCACCGTTTGCAGGAAACGGGTCGGGTTAACCGGCTTCAAGTGCAGGTGCGGGACCACGTTCTGCGGCGTCCACAGGTACCCCGAGAAGCACGCGTGTTCGTTCGATTTCAAGACGCTCGGGAGGGAGGAGATCGCCAGGAGCAACCCCCTGGTCATAGCCGAGAAGCTCGAGAAGATTTGATCCGGGCCGTCAGATCTCAATACATTTGCCAACGTACAAATCCGTGACCGGCGGATTGCAATCAATCTGGCCGTCGGCGGAGCCACGCGGAACCGTCGATCTTATCAGGGCCGTTCATCGGGCACGCGTGGGAATTAAGCTGATATAAATATTTTCTATTCCCCTAGGTTTTTAATTTAATTTTATATTAGTTTTTGCAAATAATTATTGTGGGATTTGGTTTGTAAGTCCAAAAA

**A2. Full-length amino acid sequences of 453 SAPs from 32 plant species**

>MdSAP1

MESRKDINMEPPRCAKGCGFFGSVTNMNMCSKCYRECLKEEQFAKPAAMVGLASVDKPLIVSNSTATAAVISSLPSQSSQ

GSSDSSEKKRCLSCKKRVGPTGFECRCGGVFCGKHRYPEEHSCCVDYKKTGQDLLTKQNPLCNGDKLDWRV

>MdSAP2

MESQKKMTERPCCANGCDFYGSVETKNLCSRCYXDYLKQESRENMRAESAMVAXMNNLDRGSVAGRINPLPSLKASNSVS

PSVAVAGCSKSSSGSTSVKNRCESCNRKVGVLGFSCRCGGVFCGTHRYPEKHCCHVDFKMAGRDVLAKQNPLCKGDKLEC

RI

>MdSAP3

MEPPMCASGCGFYGTVENKNMCSKCYKDHLKHETMNAASADVTSKEKLNLGSFISGISSSSYFRTSSDTSLVSEDHNFGN

NNMGTSSVGVIKKNRCQSCSRKVGVLGFQCRCGGVFCGTHRYPEEHSCDVDLKQAGRDVLAKKNPLCKGDKLEWRI

>MdSAP4

MEPPMCASGCGFYGTVENKNMCSKCYKDHLKHETMNAASADVTSKEKLNLGSFISGISSSSYFRTSSDTSLVSEDHNFGN

NNMGTSSVGVIKKNRCQSCSRKVGVLGFQCRCGGVFCGTHRYPEEHSCDVDLKQAGRDVLAKKNPLCKGDKLEWRI

>MdSAP5

MAQRAEKEETEFKVPETLTHYVNNCGVTDNPSTNNLCQKCFNTATTSSSFSSAAIXKLSAEKSPISTSSFSFEALAETFR

KTTASEIARSDESLNRRVVNRCSECRRKVNYTELSLGNKSPTFPLPFAPKIRWKLASNWVKCTDGFRGCTVAINRFYKHH

C

>MdSAP6

MTQRAEKEETEFKVLETLTHCVNNYDVTGNPSTNNMCQKCFNAATAAATTSSSSSSVTILKFSAEKSPRSTSSFSFEAPA

ETFKKTKASEIARSDESPNRCVVNQCFECRRKVGLTGFRFYLAILSIISRYISQIMH

>MdSAP7

MKKMAQRTEKEETEFKVPETLTHCVNNCGVTGNPSTNNMCQKCFNAASAAAAAATSSSSSAAILKFSAEKSPRSSSSFSF

EAAAETCRKTTASEIARSDETPNRRVVNRCSGCRRKVGLTGFRCRCGELFCSEHRYSDRHVCSYDYKAAGREAIARENPV

VKAAKIVRV

>MdSAP8

MEHNETGCQAPPEAPKLCANNCGFFGSPATMNLCSKCHKDLVLKQEQAKVVAASIDSAVNGSPSESGKGPVATAAVDVQA

GPADVMLISTQASSTPSLNIKREEKVKETPTRCGTCRKRVGLTGFSCRCGDLFCAVHRYSDKHNCPHDYRTAAQDAIAKA

NPVVKAEKLDKI

>MdSAP9

MKKMAQRTEKEETEFKVPETLTHCVNNCGVTGNPSTNNMCQKCFNAASAAAAAATSSSSSAAILKFSAEKSPRSSSSFSF

EAAAETCRKTTASEIARSDETPNRRVVNRCSGCRRKVGLTGFRCRCGELFCSEHRYSDRHVCSYDYKAAGREAIARENPV

VKAAKIVRV

>MdSAP10

MEHEETGCQAAPEGPILCVNNCGFFGSAATMNMCSKCHKDMMLKQEQAKLAASSFGSIVNGTSSIDANEPVVAAATVDVQ

PHPVEPKTLSSQPSFSFGSGSSGEPRPEGPKRCNTCNKRVGLTGFNCRCGHQFCAVHRYSDKHDCPYDYRTAGRDAIAKA

NPVVKAEKLDKI

>MdSAP11

MAEEHRCEAPEGHHLCANNCGFFGSPATMNLCSKCYRDFCLKEQQQASIKSTVEASLSASAADAASSSLSAPSSPPSTSL

PASPAAIETLCQPPPPALTLPEVAGDIIGEPAEVVRAPEVATVVSQPNRCTVCRKRVGLTGFKCRCGTTFCGVHRYPEKH

ACSFDFKTLGREEIARSNPLVIAEKLEKI

>MdSAP12

MAEEHRCEAPEGHHLCANNCGFFGSPATMNLCSKCYRDFCLKEQQQASIKSTVEASLSASAADAASSSLSAPSSPPSTSL

PASPAAIETLCQPPPPALTLPEVAGDIIGEPAEVVRAPEVATVVSQPNRCTVCRKRVGLTGFKCRCGTTFCGVHRYPEKH

ACSFDFKTLGREEIARSNPLVIAEKLEKI

>MdSAP13

MESHDETGCQAPDRPILCVNNCGFFGRAATMNMCSKCYKDTLLKQEQANLAASSIDSIVNGGGSSSSSNIFIDPVVAGVV

DVQAVRVETSVVSTEAYIESSPSMKIEMKENKGPSRCTTCRKRVGLTGFNCKCGNTFCASHRYSDKHDCPFDYRTAGQDA

IAKANPIVKADKLDKI

>MdSAP14

MKKMAQRTEKEETEFKVPETLTHCVNNCGVTGNPSTNNMCQKCFNAASAAATTSSSSSSAAILKFSAEKSPRSTSSFSFE

APVETFRKTTASEIARSDESPNRRVVNRCSGCRRKVGLTGFRCRCGELFCSEHRYSDRHVCSYDYKAAGREAIARENPVV

KAAKIVRL

>MdSAP15

MEHNETGCQAPPEAPKLCANNCGFFGSPATMNLCSKCHKDLVLKQEQAKVVAASIDSVVNGSPTESSKGPVATAAVDVQA

GSADVMLISTQASSTSLNIKSEEKVKETPTRCGTCRKRVGLTGFSCRCGDIFCAVHRYSDKHKCPYDYRTAAQDAIAKAN

PVVKADKLDKI

>MdSAP16

MESHDETGCQAPDRPILCVNNCGFFGRAATMNMCSKCYKDTLLKQEQANLAASSIDSIVNGGGSSSSSNIFIDPVVAGVV

DVQAVRVETSVVSTEAYIESSPSMKIEMKENKGPSRCTTCRKRVGLTGFNCKCGNTFCASHRYSDKHDCPFDYRTAGQDA

IAKANPIVKADKLDKI

>MdSAP17

MDSRNDTNMEPPLCAKGCGFFGSVTNMNMCSNCYRQYLKEEQFAKPAAMVGLASVDNTLSDSSSATAAVISSLPSQSSQG

SSDLSQKKRCLSCKKRVGPTGFECRCGGVFCGKHRYPEEHSCSVDYKKTGQELLTKQNPLCKGDKLHWRV

>MdSAP18

MAEEHRCQAQQLCVNNCGFFGSPTTQNLCSKCYRDLQLKEQQAVALNQTLISSSSFASPSSSSSPSPSRPFSSPLISVSP

AHKARVERVVEAKEEEEKEAAPSAGAQANRCTTCRRRVGLTGFKCRCGMTFCGTHRYPEQHACGFDFRGMGKEQIAKANP

VVKAEKLHKI

>MdSAP19

MAEEHRCQAQQLCVNNCGFFGSPTTQNLCSKCYRDLQLKEQQAVALNQTLISSSSFASPSSSSSPSPSRPFSSPLISVSP

AHKARVERVVEAKEEEEKEAAPSAGAQANRCTTCRRRVGLTGFKCRCGMTFCGTHRYPEQHACGFDFRGMGKEQIAKANP

VVKAEKLHKI

>MdSAP20

MAEEHRCQAQQLCVNNCGFFGSPTTQNLCSKCYRDLQLKEQQAVALNQTLISSSSFASPSSSSSPSPSRPFSSPLISVSP

AHKARVERVVEAKEEEEKEAAPSAGAQANRCTTCRRRVGLTGFKCRCGMTFCGTHRYPEQHACGFDFRGMGKEQIAKANP

VVKAEKLHKI

>MdSAP21

MMGGTEAFPDLGRHCQLSDCHQLDFLPFQCDGCRKVFCVEHRSYKGHECPKSDHNSRKVVVCEICSASVETTGCDGEQDH

KLLLQKHAKSGNCDPKNKKKPTCPVRRCKETLTFSNTSTCKTCQIKVCLKHRFPADHVCRKQTAAQPSLLAGNGVSWNDR

FMAAFASRKGKECGKSERDSKSSASSGPSVRAY

>MdSAP22

MAEEHRCEAPEGHHLCANNCGFFGSPATMNLCSKCYRDFCLKEQQXASIKSTEMLRVDRVQVQVRDHVLRGSQVPREARV

LVRFQDPRKGGDRQEQPVGHSREAREDLIRSVGSNTFVKIQIADRRVSINQAVRGTDDMIRTFTLSPIRQMLENSSSFGS

TSSSPSLANLPSIRVHVEDGIGGGGEDLGRVQAGEMGRSQSRVSGLGLF

>MdSAP23

MEHEETGCQAAPEGPILCVNNCGFFGSAATMNMCSKCHKDMMLKQEQAKLAASSFGSIVNGTSSINANEPVIAAPPVDIQ

SQPVAPQTISSQPSFSFGSGSSGEPKPEGPKRCNTCNKRVGLTGFNCRCGHLFCAVHRYSDKHDCSYDYLTAGQDAIAKA

NPVVKADKLGKI

>MdSAP24

MEHEETGCQAAPEGPILCVNNCGFFGSAATMNMCSKCHKDMMLKQEQAKLAASSFGSIVNGTSSINANEPVIAAPPVDIQ

SQPVAPQTISSQPSFSFGSGSSGEPKPEGPKRCNTCNKRVGLTGFNCRCGHLFCAVHRYSDKHDCSYDYLTAGQDAIAKA

NPVVKADKLGKI

>MdSAP25

MGTPEFPDLGRHCYVAECQQIDFLPFTCDSCHQVFCLEHRSYIKHNCPNGDRNNVTVVICPLCAKGVHLIPDEDPNITWE

RHVNTDCDPSNYEKATKKKKCPIPGCKEILTFSNTIKCRDCMVDHCLKHRFGPDHKCPGPKKPEAGFPFMGYLSRSRKEV

SKPSHAPAASSPKWGSFLTAASSFRASAEASVAKLSSELSQKWQIAKDGTGPSSSSSGSRNGQVEVCPQCGAKFSSVTTL

VDHVEKVHEKGGNRAAAVKKVTIDACPKCSKGFRDPVALVEHVERDHGGTSRA

>MdSAP26

MESHDETGCQAPDRPILCVNNCGFFGRVATMNMCSXCYKDTLLKQDQAXLAASSIDSIVNGVXXSSNIVIDPVVASVVDV

QXVQVGTSIVXTEPSSDSSSSMKIEVKEKKGPSKCTTCRKRVGLTGFNCKCGNTXCASHRYSDKHDCPFDYKTAGQDAIA

KANPIVKADKLDKI

>MdSAP27

MTGGTEAFPDLGRHCQLSDCHQLDFLPFQCDGCHKVFCVEHRSYKSHECPKSDHNSRKVVVCEICSTSIETTGREGEQDQ

KLLLEKHAKSGNCDPRKKKKPTCPVRRCKEILTFSNTSTCKTCKIKVCLKHRFPADHVCQKQTAAQPSLVGKPVSWNDKF

MAAFALREGKECGKSARDSKSSASSAPSVRAY

>MdSAP28

MTGGTEAFPDLGRHCQLSDCHQLDFLPFQCDGCHKVFCVEHRSYKSHECPKSDHNSRKVVVCEICSTSIETTGREGEQDQ

KLLLEKHAKSGNCDPRKKKKPTCPVRRCKEILTFSNTSTCKTCKIKVCLKHRFPADHVCQKQTAAQPSLVGKPVSWNDKF

MAAFALREGKECGKSARDSKSSASSAPSVRAY

>MdSAP29

MAEEHRCEAPEGHHLCANNCGFXGSPATMNLCSKCYRDFCLKEQQEASIKSTVEASLSASAASAAAASPPCSPPSTSLPS

SAAAIETQCQPPPPALTLPEVVGDIIKDPAGDLRAREVAEVVSQPNRCTVCRKRVGLTGFKCRCGTTFCGVHRYPEKHAC

SFDFKTLGRGGDRQEQPPGHSREAREDLIRAVRSQYICQRTNP

>MdSAP30

MAEEHRCEAPEGHHLCANNCGFXGSPATMNLCSKCYRDFCLKEQQEASIKSTVEASLSASAASAAAASPPCSPPSTSLPS

SAAAIETQCQPPPPALTLPEVVGDIIKDPAGDLRAREVAEVVSQPNRCTVCRKRVGLTGFKCRCGTTFCGVHRYPEKHAC

SFDFKTLGRGGDRQEQPPGHSREAREDLIRAVRSQYICQRTNP

>AtSAP1

MGSEQNDSTSFSPSEPKLCVKGCGFFGSPSNMNLCSKCYRDIRATEEQTASAKAAVEKSLNPNKPKTQPQQSQEITQGVL

GSGSSSSSTRGGDSAAAPLDPPKSTATRCLSCNKKVGVTGFKCRCGSTFCGTHRYPESHECQFDFKGVAREAIAKANPVV

KADKVDRI

>AtSAP2

MDHDKTGCQSPPEGPKLCTNNCGFFGSAATMNMCSKCHKDMLFQQEQGAKFASAVSGTSSSSNIIKETFTAALVDIETKS

VEPMTVSVQPSSVQVVAEVVAPEEAAKPKGPSRCTTCNKRVGLTGFKCRCGSLFCGTHRYADVHDCSFNYHAAAQEAIAK

ANPVVKAEKLDKI

>AtSAP3

MAEEHRLQEPRLCANNCGFFGSTATQNLCSKCFRDLQHQEQNSSTAKHALTQSLAAVGAAASSSVSPPPPPPADSKEIVE

AKSEKRAAAEPEEADGPPQDPKRCLTCRRRVGITGFRCRCGFVFCGTHRYAEQHECSFDFKRMGKDKIAKANPIVKADKL

EKI

>AtSAP4

MAEEHRCETPEGHRLCVNNCGFFGSSATMNLCSNCYGDLCLKQQQQASMKSTVESSLSPVIAPVLENYAAELEIPTTKKT

EEKKPIQIPTEQPSPPQRPNRCTVCRKRVGLTGFMCRCGTTFCGSHRYPEVHGCTFDFKSAGREEIAKANPLVIAAKLQK

I

>AtSAP11

MGTPEFPDLGKHCSVDVCKQIDFLPFTCDRCLQVFCLDHRSYMKHSCPKGDREDVTVVICPLCAKGVRLNPNEDPNITWE

KHVNTDCDPSNYEKATKKKKCPVPRCKEYLTFSNTIKCRDCNVDHCLKHRFGPDHTCPGPRKLPFMGFLSSSTTRKEAKT

TRPNKAHPSTSSSSSSSRWSNLLSSAEAGISRLGNDISQKLQFSSSKDNGIVEVCPQCGAKFSSVTSLVEHVEKTHERNK

KQNHGNVTVDVCPRCSRGFRDPVDLVNHIERDHRGTSKA

>AtSAP5

MAQRTEKEETEFKVLETLTTTTTTLCTNNCGVTANPATNNMCQKCFNASLVSAAAGVVESGSILKRSARSVNLRSSPAKV

VIRPREIDAVKKRDQQIVNRCSGCRKKVGLTGFRCRCGELFCSEHRYSDRHDCSYDYKTAGREAIARENPVVKAAKMVKV

>AtSAP12

MAGGGTEAFPDLGEHCQDPDCKLLDFLPFTCDGCKLVFCLEHRSYKSHNCPKSDHGSRTVSICETCSIAIETTGFDEKGI

KSLLEKHERSGDCDPNKKKKPTCPVKRCKEILTFANNLTCKYCGVKFCLKHRFPTDHVCNKKIINTAGTSSRWNERFMEA

LSLRNQKGCGRGSSVSSKSSPSVRSF

>AtSAP6

MAEEHRCQTPESNRLCVNNCGFLGSSATMNLCSNCYGDLCLKQQQQSSSIKSTVESSLSVSPPSSSSSEISSPIIPPLLK

NPSVKLEVPEKKAVISLPTTEQNQQQRPNRCTTCRKRVGLTGFKCRCGTMFCGVHRYPEIHGCSYDFKSAGREEIAKANP

LVKAAKLQKI

>AtSAP13

MGTPEFPDLGKHCSVDYCKQIDFLPFTCDRCLQVYCLDHRSYMKHDCPKGNRGDVTVVICPLCAKGVRLNPDEDPNITWE

KHVNTDCDPSNYEKAVKKKKCPVPRCRELLTFSNTIKCRDCSIDHCLKHRFGPDHSCSGPKKPESSFSFMGFLSTNTKEA

PASSSSSSRWSSLFASAEASISRLGNDISQKLQFASGNDGNSEKTQERNGKQNCGKVTVDVCPKCSRGFRDPVDLLKHID

KDHRGTSKA

>AtSAP7

MGSEENNSTSFPPTEPKLCDNGCGFFGSPSNMNLCSKCYRSLRAEEDQTAVAKAAVKNSLKLPSCSIIAPGQKHPLEIKP

AHLETVVVTAEPSSVPVAAEQDEAEPSRPVRPNNRCFSCNKKVGVMGFKCKCGSTFCGSHRYPEKHECSFDFKEVGRDAI

AKANPLVKADKVQRI

>AtSAP8

MTGEPSLCIRGCGFFSTSQTKNLCSKCYNDFLKDESARYLATFNVNTKAAEEVTAQEATVLGSKGGCACKKKVGLLGFHC

RCGHLFFASHRYPEEHSCPSDYKSAAIDVLAKQNPVVKGDKLFRL

>AtSAP9

MGSEQNDSTSFTQSQASEPKLCVKGCGFFGSPSNMDLCSKCYRGICAEEAQTAVAKAAVEKSFKPSPPRSLFIAEPPAVV

VEPKPEKAAVVVVSAEPSSSAVPEANEPSRPARTNRCLCCNKKVGIMGFKCKCGSTFCGEHRYPETHDCSFDFKEVGRGE

IAKANPVVKADKIQRF

>AtSAP10

MVNETEALPCEGGCGLYGTRVNNNLCSLCYKKSVLQHSPALRFEPETEQSQCCPPTNSPAVEEEPVKKRRCGICKRKVGM

LGFKCRCGHMFCGSHRYPEEHSCPFDYKQSGRLALATQLPLIRADKLQRF

>AtSAP14

MGTPEFPDLGKHCSVDVCTQIDFLPFTCDRCLQVLCLDHCSYMLNPDEDSNITWDKHVNDTDCDPSNYENDVKKKKKQCP

VSSCSGPKEPDSSISSTNTTVAATSAPASSSSSSISSASFFASAEARFRKTRLNRTRLNRARLNPTREDEDPRVENLPLW

MIREGVADVVGELRGQMSQEEEEEDVEWEEE

>OsSAP13

MVLSGRGMLDGGADDVGLWLGMQLPTTSIVDSPGKRSLCVRASDGAGERDAGRAAAAPVRERLWLLRLCQHPRPLLQVLP

RQPPPDRDVPGAVVIVVHGASRRGTVPEGIPVDEGAMPPPPPPRAKTKSRCAACGRRVGLMGFECRCGAVFCGAHPLLGQ

ARLWLRLQGRAGRDAIARANPVVSADKVDKL

>OsSAP2

MEQGSERQDERPPLPCANGCGFFGSADTRGLCSKCYRQTVMSQASAPSAAAQSAEHDQVVLPAPEGVPVDEGAMPPPPPR

HGAKTKSRCAACGRSVGLMGFECRCGAVFCGAHRYSDRHDCGYDYRGAGRDAIARANPVVRPDKVEKL

>OsSAP3

MGQQVQHESRINVGEATHVSKAEMGANTMFATSRLNSNNKVGPELAYLSGVASSASDSSTAAPSPCYLCHKPAALHVFGL

AGRYVFGSVKREAYLSQEGPRSGRTPNRIAESLPVRVVNDFGLRLRVVTNQGPIKPRPPRPIDAIVFASIETRNRLRGFD

RSFCCSAPPETYVFLPRARETIVLRANIIKMSSEQQASAGQPVLCASGCGFYGNPATLDMCSVCYRQHCLLNGATMATGP

SSSVAAASAATVATGAVTSDSCSVPSAEVNGAAFSSKNNPEPATVVEKKAPANRCASCKKKVGLLGFACRCGATYCGTHR

YPEKHACGFDFKGASRDAIARANPLIKGEKLTNKI

>OsSAP4

MEHKEAGCQQPEGPILCINNCGFFGSAATMNMCSKCHKEMIMKEEQAKLAASSIDSIVNGCDGGKEHIVAASGSTAVAVA

QVEAKTLVVQPTDVAGTSEEVAVVPKVKEGPNRCATCRKRVGLTGFNCRCGNMYCALHRYSDKHECQFDYRTAARDAIAK

ANPVVKAEKLDKI

>OsSAP5

MAEEQRWQEGCHRLCANNCGFFGSPATLDLCSKCYRDRQGRESTAPVVVAAAASACPATHPSSPSSSSCPAFLPSSTAAE

AGVVVAAVAKASRCASCRKRVGLTGFACRCGGTFCGAHRYPERHACGFDFKAAGRDAIARANPLIKGDKLKDKI

>OsSAP6

MAQESWKKEAEETGVHTPEAPILCVNNCGFFGSRMTENMCSKCYRDTVKAKTVATVVEKKPLASLSSTPLVTEVTDGGSG

SVADGKQVMEEDTPKPPSNRCLSCRKKVGLTGFKCRCGGTFCSMHRYADSHKCTFDYKQVGREQIAKQNPLVKADKITKI

>OsSAP7

MASMKRKCPDDETACGSGAGAAMCVTGCGFFGSEATNNMCSRCYREHSADNDAVEEAAAANSDLELVGVAETTTKKARMS

AVVPVAVASSSSAAAEQPAAKAATAPNRCAACRKKVGLTGFKCRCGGNFCGGHRHADAHGCGFDYKSAGKEQIAKQNPLV

VADKLATRI

>OsSAP14

MATKRKCPANGDDGGVADLEPVAGGSFASPPPEKKAKLTVAVAVAVAPSSSSSATTAAAGEATAKREHGGFFAFARPENN

TRLSVAVASSSSSASAAAEKAMAKLTVAGVAPSSSASAAAAGKATAKREYGGFCAFARPDDKTRWRVAVASSAAAAADAS

YSSSSPATGEQPEANRCATCRRKVGLTGFKCRCGGTFCGGHRYADEHGCGFDYKSSGRELIAKQNPVVVADKLAFRI

>OsSAP15

MAQESCDLNKDEAEILKPSSSSSPSPSPTTASPSPPTAQMTEPPPPQSTPPTPPAAAAAASAAAAPQFSAKNCEGILIEV

SKKRKLAEATATDANAVVVAAVAEPLSPVLFVNRCNVCRKRVGLTGFRCRCGELFCPRHRHSETHECSFDYKTAGREEIA

RANPVIRAAKIIKI

>OsSAP8

MEHKETGCQQPEGPILCINNCGFFGSAATMNMCSKCHKEMIMKQEQAKLAASSIDSIVNGGDSGKEPIIAGHAEVAVAQV

EVKTLVAQPAEIAGPSEGVTVNPKGREGPNRCSTCRKRVGLTGFNCRCGNLYCAMHRYSDKHDCQFDYRTAARDAIAKAN

PVVKAEKLDKI

>OsSAP9

MAQESWKNESEETVHTPEAPILCVNNCGFFGSSMTNNMCSKCYRDFVKVTTMAAPVVEKKAFTPASSSKTPLEPAKPDEV

PAAAVEDKQAAQEPPKPPSNRCLSCRKKVGLTGFQCRCGGTFCSTHRYTEAHDCTFDYKKAGRDQIAKQNPVVIAEKINK

I

>OsSAP18

MAGSKMQAGDGGGAAMCAAGCGFFGSAATGGLCSKCYKEQQPQPRHHISSAPPPGTATKWWTRSSPTSRRS

>OsSAP10

MAPGNEMQARNGGGAAMCAAGCGFFGSAATDGLCSKCYKQQQPQPRHLIGTAAGDSDKTSLKVVADLSTLVIKDNSGVGG

EGTTVMAPPATVTKAKNRCKACRKKVGLLGFPCRCGGMFCGAHACAFDYKAAGREAIARHNPLVVAPKINKI

>OsSAP16

MGTPEFPNLGKHCSVGDCNQIDFLPFTCDRCDHVFCLQHRSYTSHQCPNANQKDVTVLICPLCAKGVRLNPNEDPNITWD

THVNSDCDPSNYQKVTKKKKCPVPGCRETLTFSNTIRCKDCTKEHCLKHRFGPDHKCPGPRKPESTFPFGNMLRRSQKAE

SCSNSNSSSTSSSWWSSSLLTAATSFKSSAEAGMQKLSTATTQAIQKAKDGISTSSSNSGDLVEQCVQCPARFSTVGALI

EHCEKSHQSNSQSSRSRVTVDVCPKCSKAFRDPVLLVEHVERDHGGTSRA

>OsSAP12

MEEQQAAAAGGGGGGGGASMCANGCGFFGSEATKKLCSKCYRDQLKAAPSSPPAAPDLVANEEEEASTAAAAAADEQLAL

CSSGCGFFGSKETNNMCSKCYRDHLKATSPLFSSSSSPATASTTDITVPIAPATTAPTPSLKGKEEEATAAASSSAAAAA

KPNRCVACRKKVGLLGFECRCGGTFCSTHRHADKHACTFDFKKSDREKIAKENPLIVAPKITKF

>OsSAP11

MAQREKKVEEPTELRAPEMTLCANSCGFPGNPATNNLCQNCFLAASASSSSSSAAASPSTTSLPVFPVVEKPRQAVQSSA

AAAVALVVERPTAGPVESSSKASRSSSVNRCHSCRRRVGLTGFRCRCGELYCGAHRYSDRHDCSFDYKSAARDAIARENP

VVRAAKIVRF

>OsSAP17

MARRGTEAFPDLGAQCDREDCNQLDFLPFDCDGCGKTFCAEHRTYRDHGCARAADQGRTVVVCEACGDAIERRAGDGGGD

DAAVLEAHARSRRCDPARKRKPRCPVPRCKETLTFSNTSGCKGCGQKVCLKHRFPADHACAGAGAGAASKAAGAAAAARS

AGQCGRDAQKKEGGGWKLPQSVRNMKIF

>OsSAP1

MAQRDKKDQEPTELRAPEITLCANSCGFPGNPATQNLCQNCFLAATASTSSPSSLSSPVLDKQPPRPAAPLVEPQAPLPP

PVEEMASALATAPAPVAKTSAVNRCSRCRKRVGLTGFRCRCGHLFCGEHRYSDRHGCSYDYKSAARDAIARDNPVVRAAK

IVRF

>SlSAP1

MAQRTEKEETEFKAVPETITLCINNCGVTGNPATNNMCQKCFNATTAATSTSSSSPTGTSVTIPHNFAEK

LVRSEKSARFSSLRSSPDRKSDLDRMSQDLKKVGDTMMVKEEDQLKASLPPAKREVNRCSGCRRKVGLTG

FRCRCGELFCGEHRYSDRHDCSYDYKTAGREAIARENPVVKAAKIIKV

>SlSAP2

MEHDETGCQPHPEGPILCINNCGFFGSAANMNMCSKCYKDVILKQEQEKLAASSIENFVNGSTSQKGPVI

VGSVDVQPALLESKSVVLSSPPSSSSGEAAELMAKEGPSRCSTCKKKVGLTGFKCRCGNFYCGSHRYSDK

HDCQFDYRSAARNAIAKANPVVKAEKLDKI

>SlSAP3

MEHNETGCQPPREGPILCINNCGFFGSAANMNMCSKCYKDMVLKQEQAKLAVSSIENLVNGSSASEKGMV

IAGPVDVQPDTIEAQSIALPSSQTSSSSDMPDVKAKVGPNRCGTCKKKVGITGFKCRCGNLYCGAHRYSD

KHDCLFDYRSAGQDAIAKANPVVKAEKLDKI

>SlSAP4

GCQAPQAPVLCVNNCGFFGTAATMNMCSKCYKDMIFKQEQANFAASSIESFVNGSSNASVKAVDVAVTVQ

EGPAESLVIPTQVAVPVESEQVEKAKEGPNRCSTCRRRVGLTGFNCRCGNLFCSAHRYSDKHECPYDYRK

AGQDAIAKANPVVKAEKLDKI

>SlSAP5

MESSKETGCQAPEGPILCINNCGFFGSAATMNMCSKCHKDMILKQEQAKFAATSIENIVNGNSSSNGKEP

IATGAINVQPGSADLKVISTEASSDLSSGPSSEVKPKEGPTRCTTCRKRVGLTGFNCKCGNLFCAAHRYS

DKHECPFDYKNAGRDAIAKANPVVVAEKLNKI

>SlSAP6

FPPADPILCSNGCGFFGTAATNGLCSKCYRDFKMKEDHAAMAKVAMEKLVISRPQIESIGKVDFCSSTTS

TAAERPVVEAATAEIGGSQPNRCLSCRKKVGFVGFKCRCGSTFCGTHRYPEKHDCTFDFKIKGKEEICKA

NPVVKADKIQRF

>SlSAP7

MAEEQRMQEGGGHRLCANNCGFFGSPTTLNLCSKCYKDHCMKEEQSRTAQLAMEKTRHQQQQQQQSESTS

AYIPCTEPLSILEVSQPRETEIATRAPHVQSDTAAEVPQVQSDTVAEVPQVHTQLNDVADQAPQVQSNRC

ATCRRRVGLTGFKCRCGVTFCGSHRYPEHHGCTFDY

>SlSAP8

MAEEHEFQSQEGGRHQLCANNCGFFGNSTTENYCSKCYRDIEKQKSDAKSIDSLFSPIKKVSEKKIIEPI

VLTTDTMKTTTSNVVTPQSNRCLVCKKKMGLMGFRCKCGTIFCGTHRYPEVHACTFDFKSMGREAIAKAN

PLIKAEKLKKI

>SlSAP9

MAEEHGFEAPEGHILCANNCGFFGSPTTQNFCSKCYNEVYIKGGQQKPIDSLFPPSQLPIPSTSSILVLQ

ESTAAEEEPEVVTAAVTVAVQPISAQPNRCSACRKKVGLTGFKCRCGTTFCGTHRYPEIHGCSFDFKSIG

REAIAKANP

>SlSAP10

MAAQKREKEETELKVPESIPLCSPTLPVPSPSPPSTTTHLSVAVISNLKRSDRSSTESIDLKVSSMDDQS

RSTSAASPESMDLVGRKTGVKRQREANRSSGMGCRRKVGLMPFRCRCGEVFCSEHRYSDRHDCSYDYKAA

GREAIAKENPVVKAAKILKV

>SlSAP11

MEGGTEAYPDLGRHCQISDCHQIDFLPFTCHACLKVFCVEHRSCKSHECPKSDFNSRIVLVCEICSMSME

TTGCKVEDHKAILQKHEESGDCDPKKKKKKPTCPVKRCKGILTFSNTNTCKICRIQVCLRHKFPADHACD

PTSSSSQLLLKEPNNKFLTALLARNGKDCGNKSRASSPSPANPSVKAC

>SlSAP12

PLCAKGVHLIPDEDPNITWESHVNTDCDPSNYEKATKKRKCPVPGCREFLTFSNTIRCRECTVDHCLKHR

FGPDHKCPGRKKPEAAFSFMNFRTGSRNGEPNKAPATSSSSWASSFFKAAEAGMAKLGSGRGQSSNATNH

SGSANRQVEQCPQCTLRFSSVTALVSHVQKVHEKNDVMNLTVDVCPRCSKGFRDPVSLVEHVER

>AL4G28970

MGTPEFPDLGKHCSVDVCKQIDFLPFTCDRCLQVFCLDHRSYMKHSCPKGDREDVTVVICPLCAKGVRLNPNEDPNITWEKHVNTDCDPSNYEKATKKKKCPVPRCKEQLTFSNTIKCRDCNIDHCLKHRFGPDHTCPGPRKLPFMGFLSSSSSSSRKEAKTTRPTKAHASTSSSSSSSRWSNLLSSAEAGISRLGNDISQKLQFLSSSSDGIVEVCPQCGAKFSSVTTLVEHVEKTHERNRKQNHGNVTVDVCPKCSRGFRDPVDLVNHIERDHRGTSKA*

>AL5G07400

MAGGTEAFPDLGEHCQNLDCKLLDFLPFTCDGCKLVFCLEHRSYKSHSCPNSDHGSRTVSICETCSLAIETTGFDEEGIKSLLEKHERSGDCDPNKKKKPICPVKRCKEVLTFANNLTCKDCGIKFCLKHRFPTDHVCNKKTITTARTSSRWNERFMEALSLRNEKGCGRGSSVSSRSPPSVRSF*

>AL5G27980

MGTPEFPDLGKHCSVDYCKQIDFLPFTCDRCLQVFCLDHRSYMKHDCPKGNRGDVTVVICPLCAKGVRLNPDEDPNITWEKHVNTDCDPSNYEKAVKKKKCPVPRCRELLTFSNTIKCRDCSIDHCLKHRFGPDHSCSGPKKPDSSFSFMGFLSTNTKEAPPPSSSSSSSRWSSLFASAEASISRLGNDISQKLQFASGSDGNSGKTQEKNGKQNCGKVTVDVCPKCSRGFRDPVDLLKHIDKDHRGTSKA*

>ATR_00019G03420

MARTGTEAFVDLGKHCSWAECNQLDFLPFTCDGCRRDFCAEHRSYESHDCSKAHPKNRVVILCDLCSASMETAEDLEKHQENSRECEENRKQKQKKRCSVGGCREILTFSNTIACKSCGMKTCLKHRFPRDHACGACAVAAAATSRVRVDKGQALRVH*

>ATR_00103G00670

MLTLTNFLDDLAMGTPEFPNLGRHCSVDDCRLIDFLPFTCDRCDQVLCLEHRSYSKHHCSGPNKDDVTVLVCPLCAKGVRLDPEEDPNITWESHVRTDCDPSNYQKATKKPRCPVPGCKETLVFSNTIRCKDCNRDHCLKHRFGLDHKCPGPKKPEPSFSFSGFLRGQKNEPSGPKWAWKTETTGSRRAQPQVPWGPSLLSAASSMRASAESSFAKLSIATSEALQKAKEGAMASWEAKGQGSGALAEKCRQCGAQFGDVADLINHVERVHEGPMNNGEAIDVCPKCQRGFRDPVLLVEHVEKDHRGSSRS*

>BR04G25000

MGTPEFPDLGKHCSVDICKQIDFLPFTCDRCLQVFCLDHRSYMKHTCPKGDREDVTVIICPLCTKGVRLNPNEDPNITWEKHVNTDCDPSNYEKATKKKKCPVPRCKEQLTFSNTIKCRDCSVDHCLKHRFGPDHTCPGPRKPEPPRFLGFMSSGSSSKKEAKTITRPNKPSSSSRWSNLLSSAEAGITKLGNDISHKLQFSSSSSSGGNDGTVEVCPQCGAKFSSVTVLVEHVEKTHERNKKQQNHGKSHKY*

>BR06G32400

MAGRGGTEAFPDLGEHCQNPDCKLLDFLPFTCDGCKLVFCLEHRSYKSHDCPNSDHGSRTVSICETCSVAIETTGFDQEGIKSLLEKHERSGDCDPSKKKKPICPVKRCKEVLTFANNITCKDCGVKFCLKHRFPTDHVCNKKTVANSGTRSRWNEKFMEALSLRNEKGCGRGTTSVSSSSSPSIRSF*

>BR07G15250

MGTPEFPDLGKHCSVDYCKQIDFLPFTCDRCIQVFCLDHRSYMKHSCPKGNRGDVTVVICPLCAKGVRLNPDEDPNITWEKHVNTDCDPSNYEKTVKKKKCPVPRCRELLTFSNTIKCRDCTIEHCLKHRFGPDHSCSGPKKLESSFSFMGFLSTTNTKEAPASSSSRWSSLLASAEASIRELGNDISQKLQFSSGGDGNLEKTQERNGKVTVDVCPKCSKGFREPGELLKHIDKDHRGTSIA*

>BR09G37980

MGTPEFPDLGKHCSVDDCKQIDFLPFTCDRCTQVFCLDHRSYNNPNCPKGNRGDVTVVICPLCAKGVRLNPDEDPNITWEKHVSSNCDPSSYEKTVKKKKCPVPRCRELLTFSNTIRCRDCSVEHCLKHRFGPDHGCAGPKKPESRWSSLLASAEASISRLGTDLSQKLQFANGNSEKMPEKNGKVTVDVCPKCSEGFVIRWIY*

>BV0G13940

MGTPAFPNLGKHCFVDDCKQIDFLPFTCDRCHQVFCLEHRSYKQHQCPHGDNQGVTVVICPLCAKGVRLIPDEDPNITWESHVNTACDPKNYEKVTKKRKCPVTGCKETLVFSNTVKCKDCKIDHCLKHRFGPDHKCSGPPKSNTGFPFMGLLNTTSRKEVSRSNQPVQSKSSAWSRFVSAASSARASAEAGMAKLSSDFNQTLQVGQGSNSTVNTGGSGGQEVCPQCNVRFSSVTALVEHVEKVHERSNNRSSVRKLSIDVCPKCSKGFRDPVALVEHVEKDHGGTSRV*

>CL04G04410

MGTPEFPNLGKHCNFQDCKQIDFLPFTCDCCNQVFCLEHRSYNRHSCPKADKWDVTVVICPLCAKGVRLIPEQDPNITWEVHVNTECDPSNYDKVTKKKKCPVPRCREFLTFSNTIKCRDCLVDHCLKHRFGPDHKCPGPKKPEPAGFPFVGLLSRSRKEEKGPKKALPTSSSSKWTTAFLNAASSVKASAEASMTKLSNEFSQTWITSGSSSNGRGGNGQVEQCPQCSSKFSSVSSLIDHVEKVHERGGNRPGAKTVVIDACPKCSRGFVDPVALVEHIEKDHGGTSRA*

>CL10G12700

MAAAGGTEAFPDLGRHCQHSDCHQLDFLPFTCDGCQKVFCLEHRGLKSHDCPKSDRNSRKVVVCEICSTSIETTGKDGGDETRILERHHESGDCDPSKKKKPTCAVRRCREILTFSNTCVCKTCRMKVCLKHRFPAEHGCGRPSSTAAAVVDKGRWNEKFMAALGLRSGSDCGKRERPTAAKGSSPTAPSVKAC*

>CM00011G08720

MAAAGGTEAFPDLGRHCQHSDCHQLDFLPFTCDGCQKVFCSEHRGFKSHDCPESDRNSRKVVVCEICSTSIETTGKDGGDETKILERHHESGDCDPSKKKKPTCAVKRCREILTFSNTCVCKTCRLKVCLKHRFPAEHGCGRPSSTAGAVADKGRWNEKFMAALGLRSGEDCGKRERPRTGAKGSSPTAPSVKAC*

>CM00058G00270

MGTPEFPNLGKHCNFAVCKQIDFLPFTCDCCHQVFCLEHRSYNRHSCPKADRRDVTVVICPLCAKGVRLIPDQDPNITWEIHVNTECDPSNYDKVTKKKKCPVPGCRELLTFSNTIKCRDCSLDHCLKHRFGPDHKCLGPRKLEPTGFPFVGLLNRSRKEEKGPKKALPTASSSKWTAFLNAASSVKASAEASMTKLSNELSQTWISSGSSSNGVGGNGQVEQCPQCSAKFSSVSSLIDHVEKVHERGGNRTGAKKVVIDACPKCSRGFVDPVALVEHIERDHGGTSRA*

>CP00014G00830

MGGGTEAFPDLGDHCQHPDCHQLDFLPFHCDGCKKVFCLEHRSYKSHECPKSEHNSRRVVICGICSTSLETTGFDEDSARLMVERHKKSMDCDPSKKKKVVCPVRRCKEVLTFSNTSTCKVCHLKVCLKHRFPAEHSCNQALKATVMAGNGRWNDKFLVALGLRNGKDCGGRPTSPTSTSKHSVKAC*

>CP00092G00400

MNTGYEKFSQVYCLEHRSYFKHNCPKADRGDVTVVICPLCAKGVRLNPDEDPNITWEAHVNTECDPSNYDKVTKKKKCPVPGCREVLTFSNTIKCRDCTLDHCLKHRFGPDHKCPGPKKPDAGFAFMSLLSRSRKEESKTYQAPATSSSKWTSTFLSAASTVRASAEASMAKLSSEISQKLQIARDGVVQGNNSNGNGGAVKMETCPQCGAKFLSVAALVDHVEKVHERNSKQGRAKVTIDVCPKCSRGFRDPVALVEHVERDHGGTSIA*

>CRU_004G23260

MGTPEFPDLGKHCSVDVCKQIDFLPFTCDRCLQVFCLDHRSYMKHSCPKGDREDVTVVICPLCAKGVRLNPNEDPNITWEKHVNTDCDPSNYEKATKKKKCPVPRCKEQLTFSNTIKCRDCNIDHCLKHRFGPDHTCPGPRKLPFMGFLSSGTSRKEAKTARPNKASASTSSSSSSRWSNLLSSAEAGISRLGNDISQKLQFSSSSNDGIIEICPQCNAKFSSVTTLVEHVEKTHERNKIQSHGKVTIDVCPKCSRGFRDPVDLVNHIERDHRGTSNA*

>CRU_005G06160

MNSSRTYQEVRQEKLIAMAGGGTEAFPDLGEHCQNPDCKLLDFLPFTCDGCKLVFCLDHRSYKSHNCPKSDHGSRTVSICQTCSIAIETTGFDEEGIKSLLEKHEKSGDCDPNKKKKQTCPVKRCREVLTFANNLTCKDCGVKFCLKHRFPTDHVCNKKTVTNSGTSSRWNEKFMEALSLRNEKSCGRGSSVNSSRSSPSVRSF*

>CRU_005G22990

MGTPEFPDLGKHCTVDYCKQIDFLPFTCDRCLQVFCLDHRSYMKHDCPKGNRGDVTVVVCPLCAKGVRLNPDEDPNITWEKHVNSDCDPSNYEKAVKKKKCPVPRCRELLTFSNTIKCRDCSIDHCLKHRFGPDHCCSGPKKPESSFSFMGFLSTNTKEPPASSSSSWASLFSRLGNDISQKLQFSSGSDGSSEKTQERNGKQNCGKVTVDVCPKCSRGFRDPKDLLKHIDKDHRGTSKA*

>CS00015G00780

MGGGTEAFPDLGSHCQHQDCHQLDFLPFKCDGCHKVFCFEHRSFKSHECPKSDIKSRKVIVCEVCSVSIETTGEFGEGEKTMLEKHKKSGDCDPRKQKKPSCPVKRCKEKLTFSNTATCKTCNLKVCLKHRFPADHSCKKDSLLGKNAAADAAVGKGRWNDKFLFALASRNGKECSKCDRGSSSSSPSVKAY*

>CS00137G00060

MGTPQFPDLGKHCSLPDCKQIDFLPFTCDCCHKVFCLDHRSYNQHQCPKAERHDVTVVICPLCAKGVRLRPDENPHITWENHVNVDCDPSNYDKVTKKRKCPVRGCKEILVFSNTIKCRDCTVDHCLKHRFAPDHSCPGPKKLDTGFAFMSLLNRSRKEEPKPKQSPATSSTKWTSSFLNAASSFRASAEAGMSKLSNEISQKLQIAKDGAGQSSSSGTGGTELCPQCGARFSSVTTLIEHVEKIHDRSGNQARPKVTVDVCPKCSQGFCDPVALVEHVERDHGGTSRA*

>EG0001G19860

MGTPEFPDLGKHCTVDDCKLIDFLPFTCDRCNQVFCLEHRSYIKHRCPKADKKDVTVVICPLCAKGVRLNPDEDPNITWESHVNIECDPSNYDKVTKKRKCPVPGCREILTFSNTIKCKDCTLDHCLKHRFGLDHNCAGPKKPDATFPFVNFLNRSKKEVSKSSGSPATSSKWSARFLNAASTVRASTEAGMAKLSNELNRTLQIAKDGVGQSGSSVSSAGSGSGSRQLEECPQCGAKFSSVTSLIEHVEKVHESSGNRAPVRKVTIDVCPKCSRGFRDPISLVEHVERDHGGTSTA*

>EG0009G15720

MAGGGTEAFPDLGRHCQNPDCNLLDFLPFTCDGCRKVFCAEHRSYKSHGCPGSDHRSRRVVVCEACSAAIEATGEDERAALARHEGSGDCDPRRKKKPVCPVRRCREVLTFSNTSACKACGIKVCLKHRFPADHSCGTGAAAARAAAAAGGRWNDRFVAVLASRGAKEYGKKERGSSSPPSTPTVKAC*

>FV0G38110

MSGGTEAFPDLGSHCQHPDCHQLDFLPFKCAGCHDVFCLDHRSYKSHDCPKSDHNSRKVIVCEVCSTSIETTGHDGEESQKVLLERHSKSGNCDPRKKKKPRCPVTRCKQILTFSNCSTCKTCHLKVCLKHRFPADHECNKQARAAVAVAVNEGAWNGKFLAAFASRNGKDCGKSERGSKSPPSTPSVGAF*

>FV6G47890

MGTPEFPDLGKHCSVVECQQIDFLPFTCDSCYKVFCLEHRSYIKHNCPKGDRQNVTVVICPLCAKGVHLIPDEDANITWERHVNTDCDPSNYEKATKKKKCPVPGCREVLTFSNTIKCRDCMADHCLKHRFGPDHKCPGPKKVEAGFPFMGLLNRSRTETPKRNHAPATSSPNWGSSFLSAASSFKASASAGVAKLTNELGQKWQTTKDGVGQSSSEGSRSGQVEECPQCGAKFSSVTTLIDHVQKVHEKGGVRAGVKKLTVDACPKCSKGFRDPVALVEHVEKEHGGTSRA*

>GM02G31550

MGTPEFPDLGKHCAVSDCKLIDFLPFTCDRCDQVYCLEHRSYIKHLCTKADKQDVTVVICPLCAKGVRLVPDQDPNITWENHVNTECDPSNYEKVTKKKKCPVPGCREILVFSNTIKCRDCTVEHCLKHRFGPDHKCPGPKNVESSFSFMNLLNGSKKQESKPKSSATTSSKWSTSFLNAASNIRASAEAGVSKLSAWQTARGGVGQSHSSGQVEQCPQCGAKFSSVTTLVDHVQKVHERSGNRSGAKVTIDVCPKCSRGFRDPVALVEHVEKDHGGSSRS*

>GM03G29400

MGTPEFPDLGKHCSVSYCKQLDFLPFTCDRCNQIYCLEHRGYIKHKCTKANKHDVTVAICPLCAKGVRLIPDQDPNITWDNHVNVDCDPSNYEKITKKRKCPAPGCKEVLVFSNTIKCRDCLKDHCLKHRFGPDHKCSGPKKLETSFSFMSLLNRSRKEEPKPNLTSTAPSKWSSSFLKVASNIRASAQVSMSKLGDEINQVWQAATTRDGVGQSSGSGNRNDHQVEQCPQCGSKFSSVTALVDHVQKVHERNGNRLGVKKIDACPKCSRGFLDPVSLVEHVERDHGGGSRA*

>GM10G17510

MISSRGWSVRRERGSVKVSIERLPLSSDEAATLHSHPPNFSLFLSLGFRHLSLIKLISISFLRLCLKVCSMGTPEFPDLGKHCAVSDCKLIDFLPFTCDCCDQVYCLDHRSYNKHQCTKADKQDVTVVICPLCAKGVRLVPDQDPNITWENHVNTECDPSNYEKVTKKKKCPVPGCRVILVFSNTIKCRDCTVEHCLKHRFGPDHKCPGPTKVESSFSFMNLLNGSKKQESKPKSSATSWSTSFLNAASNVRASAEAGVSKLSSWQTARGGVGQSHSSGQVEQCPQCGAKFSSVTTLVDHVQKVHERSRNRSGAKVTIDVCPKCSRGFRDPVALVEHVERDHGGSSRS*

>GM13G04630

MASGGTEAFPDLGKHCQHRDCNQLDFLPFTCDGCQQIFCLEHRSYKSHACLKSDHNSRKVVVCEACSMSIETTGHVGQDEEAILQKHLKSGNCDPTKKKKPICPVKRCREVLTFSNTSTCKTCHIKVCLKHRFPADHACSRGASASSSACVSNGLWNNRFLTAFAKRTGQECAKNGATCSTSPPSTPSVKAY*

>GM19G01640

MASGGTEAFPDLGKHCQHRDCNQLDFLPFTCDGCQQVFCLEHRSYKSHSCPKSDHNSRKVVVCEACSMSIETTGNVGQDKKAILQKHLKSGICDPTKKKKPTCPVKRCKEVLTFSNTSTCKTCHIKVCLKHRFPADHACSRGASASVANGLWNNRFLAAFG*

>GM19G32220

MGTPEFPHLGKHCSVSYCKQLDFLPFTCDRCNQVYCLLHRGYIKHKCTKANKQDVTVAICPLCAKGVRLIPDQDPNITWDNHVNVDCDPSNYEKVTEKRKCPAPGCKEVLVFSNTIKCRDCLKDHCLKHRFGPDHKCSGPQKLETSFSFMGLLNRSRKEGPKPNLASTTSSKWSSSFLNVASNIRASAETGMSKLSDEINQVWQAATTRDGMGQSSGSGNRNDHQVEQCPQCRSKFSSVTALVDHVQQVHERNGNRLGVKKIDACPKCSKGFLDPVSLVEHVERDHGGGSGA*

>GR01G25920

MAGGTEAFPDLGKHCQFSGCYQLDFLPFKCQACHKVFCVEHRSCKSHECPEPEHNSRKVIICEICSMSIEITGKEDQEKMILEKHEKSGNCDPRKKKKPTCPVKRCKETLTFSNRTICKTCRLEVCLKHRFPADHACKQASSSTTPAAAAGGSWNEKFLVAFGLRNGKDCGKSGRPSSSTTPFLKAY*

>GR02G23040

MGTPEFPDLGRHCSVQHCKQIDFLPFTCDRCDLSYCLEHRSYIKHQCSKAGNNDVTVVICPLCAKGVRLIPNEDPNISWENHVNTECDPSNYDKVTKKKKCPVRRCKEVLTFSNTIKCKDCSVDHCLKHRFGLDHDCPGPKKPATTSSSFWATSLLNVASSFRENRQVARDGAGLRRSSSGSAGQMEECPQCRVKFSSVTALVEHVKKVHERNSQSRVLKMSIDVCPKCGRGFRDPVSLVEHVEKDHGGTSKA*

>GR06G13740

MGTPQFPDLGKHCSVEDCKQIDFLPFTCDRCHLVYCLEHRSYIKHHCPKADKKDVTVVICPLCAKGVRLIPDEDPNITWEMHVNTECDPSNYDKVTKKKKCPVPGCREVLTFSNTIKCRDCTLDHCLKHRFGPDHKCPGPKKSDPGFPFMGLLSRSRKESKTNQAPATSSSKWATSFLNAASTVRASAEAGMTKLGSEISQKLQIARDGVGLSSSGGGSNGNAGQVEECPQCGAKFSSVTTLVDHVEKVHERNNQSRVFKMSIDVCPKCSKGFRDPVALVEHVERDHGGTSKA*

>LJ2G016120

MASGGTEAFPDLGKHCQHHDCHQLDFLPFTCDACKQVFCVEHRSYKSHACPKPDHNSRKVVVCETCSMSIETTSQVGQEEEEALLKRHLKSGNCDPNKKKKPICPVKRCKEVLTFSNASTCKTCQVKVCLKHRFPADHACGRVASASTSSSAAVNGGWNNRFLDALASRIKGQACTKSAERRSTSPPSTPSVKAY*

>ME02631G00030

MGTPQFPDLGKHCTREDCKQIDFLPFTCDRCLQVFCLEHRSYAKHGCPKADRQDVTVVICPLCAKGVRLNPDEDPNISWETHVNTECDPSNYEKVTKKRKCPVRGCREVLTFSNTIKCRDCTIDHCLKHRFGPDHDCPGPKKLEAGFQFLSLLNRSRKEESKPNKTPAASSTKWASAFRNAASTVRASAEAGVAKLSTEISQAWQTAKSPAGPSSSNGRDAIGLEEECPQCGARFSSVMVLVEHVQKVHERSANQSRVLKLPVDVCPKCSKGFRDPVALVEHVERDHGGTSKA*

>ME10833G00010

MAAGGTEAFPDLGRHCQHSDCNQLDFLPFKCQGCQKVFCLEHRSYKSHDCPKSDHNSRKVVVCEICSTSIETTGRDEDDEKLILEKHLKSGNCDPKKKKKPTCAVRRCKEILTFSNTCICKTCELKVCLKHRFPADHSCNKASSTPAATAASGGLFNNKFLAALASRMGKDWAKDQPSSSKTKSNTLSVKVF*

>MT00G39420

MGTPEFPDLGKHCADSDCKLVDFLPFTCDRCYQVYCLEHRSYIKHRCTKADKQDVTVVICPLCAKGVRLVPEQDPNITWENHVNTDCDPSNYEKVTKKKKCPAAGCKEILVFSNTIKCKDCTIDHCLKHRFGPDHKCPGPKKVETNFPFMNLMNRSRKKESKTNSSSTSSSSKWTTSFLNAASNIRASAEAGMSKLSGEINQAWGTSSDGGRKSNDNGQVEQCPQCGAKFSSITTLINHVQKVHERSGSRSAANVTIDACPKCSKGFTDPVSLVEHVEREHGGTSRR*

>MT6G008210

MASGGTEAFPDLGKHCQHVDCHQLDFLPFTCDGCKQVFCVEHRSYKSHECPKPDHNSRKVVVCEECSMSMEIRGNMGENEEAILKKHRSSGKCDPSKKKKPTCPVKRCKEILTFSNTSTCKTCHIKVCLKHRFSADHACSKGDSSSLTTTAGNGRWNNRFMAALASRNGQDCGKKSGSRSTTSPPSNPSVKAC*

>MT7G091810

MGTPEFPDLGKHCSVSDCRLIDFLPFTCDRCNQVYCLGHRSYIEHNCTKPNKQDVTVVICPLCAKGVRLIPDQDPNIVWEHHVNTDCDPSNYEKATKKKKCPVPGCRETLVFSNTIKCRDCEVDHCLKHRFGPDHKCPGPKKLETSFSFMSLWNMSSGKEVSKPNLSSTTSSKWTSSLLDMASKLSGIGQSSGSGTSNNQLEKCPLCDAKFSSAASLVDHAKKVHQRSGGNQYGAKKVSISACPKCSKGFLDPVSLVEHVERDHGGCS*

>PP00083G00800

MGTPALPDLGQHCSRVDCHQLDFLPFTCDACHKVFCLEHRQYKSHNCPNTKDHDVTVIVCPVCHKSIRTVANEDENVTWDRHVRTNCDPSNYEKATKKPRCPVRGCKEILVFSNKVLCNDCKREVCLKHRFGLDHGCEDFRKANRSNWGISDYGNMFMKSFKERKAPVVQSNGNSKLPANGASSVQSGSIKGAFSGLFSSVEAGINKLGLATSSSGTSHGNAVGRQTPSEPLKGSSGQASKTEECPQCRARFANVAQLIKHVETMHDTPNQEMLDECPKCGRKFRDPIQLVNHVERDHGGSSS*

>PP00325G00370

MEFPDLGLHCSEETCHQLDFLPFKCDGCRKVFCLEHRTYKAHSCANANHKDVSVQICPVCAMSVKTVFGETVELTMKKHQQSKTCDPRNYVKMTKKPKCPVRGCRELLTFSNKYCCNACQKTVCLRHRFPSDHACGIAPTRATAQTAAGSKFLASFASRHSDMDCGAESNRMASLYIFEKKIDQEKTPTVKP*

>PP00415G00090

MEFPDLGLHCSEETCHQLDFLPFKCDGCRKDFCLEHRAYKAHSCANANHKDVSVQICPVCAMSVKTVFGETVGLTMKKHQQSKTCDPRNYVKVTKKPKCPVRGCRELLTFSNKYCCNSCQKTVCLRHRFPSDHACGIAPTRATAQIAAGSKFLASFASRHSDMHCGAESNRMASLYISEKRIDQEKTPTVKP*

>PPE_003G25710

MTGGTEAFPDLGRHCQHSDCHQLDFLPFQCDGCHKVFCVEHRSFKSHECPKSDHNSRKVLVCEICSTSIETTGRDGEKDQMLLLEKHHKSGNCDPRMKKKPTCPVRRCKEILTFSNTSTCKTCQVKVCLKHRFPADHVCRKQTTPSSSLLVGNGVHWNEKFMAAFASRKGKECGKSERDSKSSSTTPSVRAY*

>PPE_007G07700

MGTPEFPDLGRHCYIADCQQIDFLPFTCDSCHQVFCLEHRSYIKHNCPKADKQNVTVVICPLCAKGVHLIPDEDANITWERHVNTECDPSNYEKATKKKKCPVPGCKELLTFSNTIKCRDCMVNHCLKHRFGPDHKCPGPKKPTTGFPFSGLLSRSRKEVSKPNHAPAASSSNWSNSFLTAASSFRASAEASVAKLSSELSQKWQIAKDGVGQSSNSGSRNGQLEECPQCGAKFSSVTALVDHVEKVHEKGGNRAGVKKATIDACPKCSKGFRDPVALVEHVERDHGGTSRT*

>PPE_007G07720

IDFLPFTCNSCHQVFCLEHRSYIKHNCPKANKQNVTVVVCPLCAKGVHLIPDEDANITWERHVNTECDPSNYEKATKKKKCPVPGCKELLTFSNIIKCRDCTVNHCLKHQFGPDHKCPGPKKPTTSFPFSGLLSGTSSFRASAEASVAKLSSELSQKWQIAKDGAGQSSSSGSRNGQLEECPQCGAKFSFVTTLVDHVEKVHEKGGDRAEVKKATIDACPKCSKGFRDPAALVEHVERDHGGISRT*

>PT06G05220

MGTPQFPDLGKHCSVEDCKQIDFLPFTCDRCRQVFCLEHRSYIKHSCPKADSNGVIVVICPLCAKGVRLNPDEDPNISWEVHVNTECDPSNYDKVTKKRKCPVRGCRELLTFSNTIKCRDCTLDHCLKHRFGPDHTCPGPKKPDVSFPFMGLLNRSKKEESKPNRATAVSSSKWTTNFLSAASTVRASAEAGMSKLSSEISQAWQTATNSASPSSSNGSGGMGPEECPQCGTRFSSVTNLIDHVQKVHEKGGNQSRVLQLPMEVCPKCSKGFRDPVALVEHVERDHRDNLVEPT*

>PT11G13850

MGGGTEAFPDLGRHCQHSECKQLDFLPFNCNGCRKVFCLEHRSYKSHECPKSDHKSRKVVVCETCSASIETTGCNEDAEKVVLLKHEKSGDCDPRKKKKKKPTCAVKRCKEILTFSNTCTCKTCQLKVCLKHRFPADHACKKYHPLQYM*

>RC29761G00070

MIVLPWNHCHVFCLEHRSYAKHDCPKADSKDVTVVICPLCAKGVRLNPDEDPNLSWESHVNTECDPSNYEKVTKKRKCPVRGCREVLRFSNTIKCRDCTIDHCLKHRFGPDHDCPGPKKPELGFQFLSLLNRSKKEDSKLNQTPAASSTKWSAAFRNAASSVRASAEAGMTKLSSEISQAWQTATNGAGPSSSNRLEEECPQCHARFSSVTTLVEHVQKLHERNGNRSRVMNLPVDVCPKCSKGFRDPVALVEHVERDHGGTSKA*

>RC29822G00800

MAAGGTEAFPDLGKHCQHSECNQLDFLPFQCNRCQKVFCLEHRSYKSHECTKPDQNSRKVVVCEICSSSIETTGRNEDDEKLILEKHVSSGDCDPKKKKKPTCGARRCKEVLTFSNTCICKTCHLKVCLKHRFPADHECNKASSAGAAAAGGGGRRSNDKFLVAWASRNGKDCAKNAKPSSSKPSIFSVKIF*

>ST02G035660

MEGGTEAYPDLGRHCQLSDCHQLDFLPFTCHACQKVFCVEHRSCKSHECPKSDFNSRMVLVCEICSMSIETTGCQVEDHKAILRKHEESGDCDPKKKKKPTCPVKRCKGILTFSNTSTCKTCRIQVCLRHRFPADHPCNRTSSSSQPLVKEANNKFLTALLARNGKDCGNRSRASSSTPANPSVKAC*

>ST04G012830

MGTPEFSNLGKHCSVEDCRQIDFLPFTCDCCYKVYCLDHRSYIRHQCPTANKNNVTVVICPLCAKGVRLNPVEDPNITWESHVNTECDPSNYEKATKKKKCPVPRCKELLTFSNTIKCRDCTVDHCLKHRFGPDHSCPGPKKPESTFQFMNFLNGSKEESKKAQPTTTSRWTTSLLKVVTSVKEKFNSEFNQPQQTGQSSRATNHSVTNNSSQVEPCPQCHLRFSTVRALIDHVQKVHEKNGVMNMTIDVCPRCSKGFRDPVALVEHVEREHKGSSM*

>ST10G021950

MGTPEFPNLGKHCFVDDCRQIDFLPFTCDCCHQVYCLEHRSYNRHHCPTANKNDVTVVVCPLCAKGVHLIPDEDPNITWESHVNTECDPSNYEKATKKRKCPVPGCREFLTFSNTIKCRECTVDHCLKHRFGPDHKCPGRKKPEAAFSFMNFRTGSRKDEPKKAPATSSSSWASSFYKAAEAGIAKLSTGRGQSSNATNHSGNANGQVEQCPQCTLRFSSVTALVSHVQKVHEKNGVMNLTVDVCPRCSKGFRDPVSLVEHVEREHKGTSKA*

>TC0004G00360

MVTRKRTSSNIDKNKQGDKPTQLFPLEVSQQLYITEIKHGIRFQARLGLGHKTTSLYKGLPSLASAQNILNSDKSLLTLSMGGGTEAFPDLGRHCQHSDCHQLDFLPFKCDGCHKVFCLEHRSYKSHECLKSDHKSRKVVVCEICSTSIEIRAEGEEEKMVLERHEKSGDCDPTKKKKPTCPVRRCKQILTFSNTSVCKTCRLKVCLKHRFPSEHACKQTSTAPAEAARGGWNDKFLAALASRNGKDCAKNGRRSSSPTTPSVKAY*

>TC0005G15970

MGTPEFPDLGKHCSVEDCKQIDFLPFTCDRCRLVFCLEHRSYIKHRCPKADKKDVTVVICPLCAKGVHLIPDEDPNITWETHVNTECDPSNYDKVTKKKKCPVPGCREVLTFSNTIKCRDCTIDHCLKHRFGPDHNCPGPKKPDSGFPFMGLLSRSRKEESKPNRAPATSSTNWATSFRNAASTVRATAEASMTKLSSELSQKWQIARDGAGLSGSSSSSSNGSTAQVEECPQCGAKFSSVTTLVEHVEKVHERNNQSRVFKMSIDVCPRCSKGFRDPVALVEHVERDHGGTSKA*

>TP2G17110

MVGGRGGTEAFPDLGEHCQNPDCKLLDFLPFTCDGCKLVFCLEHRSYKSHDCPKSDHGSRTVSICETCSIAIETTGFDEEGIKSLLQKHERSGDCDPKKKKKPICPVKRCREILTFANNLTCKDCGVKFCLKHRFPTDHVCNKKSVANSGTSSRWNEKFMEALSLRKEKGCGRGTTSVSSNSPSIRSF*

>TP4G24100

MGTPEFPDLGKHCSVDICKQIDFLPFTCDRCLQVFCLDHRSYMKHSCPKGDREDVTVVICPLCAKGVRLNPKEDPNITWEKHVNTDCDPSNYEKATKKKRCPVPRCKEQLTFSNTIKCRDCNIDHCLKHRFGPDHTCPGPRKPEPSFPFMGFLSSSSRKETKTTRHNKAPASTSSTSSSSSSRWSNLLSSAEAGISRLGNDISNKLQFSSSSNDGLVEVCPQCGAKFSSVAVLVEHVEKTHERNKKQNHGKVTVDVCPKCSRGFRDPVDLVNHIERDHRGTSKA*

>TP5G05120

MGTPEFPDLGKHCSVDYCKQIDFLPFTCDRCIQVFCLDHRSYMKHNCPKGNRGDVTVVICPLCAKGVRLNPDEDPNITWEKHVNTDCDPSNYEKTVKKKKCPVPRCRESLTFSNTIKCRDCNIDHCLKHRFGLDHSCSGPKKPESSFSLMGFLSTNTKEAPASSSSSRWSSLLASAEASISRLGNDISQKLQLTSGGDGNLEKMQESNGKQIRGKVTVDVCPKCSKGFRDPKDLLKHIDKDHRGTSKA*

>VV08G07580

MGTPEFPDLGKHCTVDDCKQIDFLPFTCDRCHQIFCLEHRSYIKHHCPTADRKDVTVVICPLCAKGVRLIPDEDPNVTWETHVNTECDPSNYEKATKKKKCPVSGCREILTFSNTLKCRDCTIDHCLKHRFGPDHKCPGPKKPEAAFPFIGLLNRSKKEDSKPSRAPTSSSKWATSFLNVASNVRASAEAGMTKLTSEFSQAWQTTRDGVTIDVCPKCSRGFRDPVSLVEHVERDHGGTSKA*

>VV14G12190

MGEGTEAFPDLGRHCQRSDCNQLDFLPFKCDGCHQVFCLEHRTYKSHECPKPEPNSRKVVVCETCSTSIETTGRDEKEEKAMLERHEKSGDCDPSKKKKPICPVRRCKEVLTFSNTSTCKTCQLNFCLKHRFPIEHACKQRLPSSTTGAKERWSDKFLAALGARIGNDCAKNGGRTVSPPGTSHPVKAC*

>ZM02G30140

MTCPDSGLGTRLLLAAKERKAKRFFFRVGNSDTHSRPAQVYKCTLVLSSLAHHPMTQSIANNPINRGRSRKEVEGRRPMARRGTEAFPNLGAHCDKPDCNQLDFLPFDCDGCGKVFCAAHRTYGDHGCAKAADQGRTVVVCPDCGDAIERLVPGQGEREILEAHVRSRRCDPGKKRKPRCPARRCREQLTFSNTQDCKGCGRKVCLKHRFPADHDCAASAPGAAAAARRASGECGRGARKEGSGGWALPASIRSLKIF*

>ZM07G24750

MGTPEFPNLGKHCSVGDCNQIDFLPFTCDRCDHVFCLDHRSYTSHQCPNANMKDVTVLICPLCAKGVRLNPSEDPNITWDTHVNTDCDPSNYQKVMKKKKCPVPGCRETLTFSNTIRCKDCTREHCLKHRFGPDHKCPGPRKVDSGFPFVSMLRRSQKAETRSNSSNNNGSSWWSSSLVNAATNFKSSAEAGMQKLSTVTSQAFQKAKDGIAPNSSSSSGDLVEQCVHCPARFSTVGALIEHVEKSHQINSQPSHGRVTIDVCPKCSKAFRDPVLLVEHVEKEHGGTSRV*

>AL0G05070

MAEEHRCQTSESNRLCVNNSGFLGSSATMNLCSNCYGDLCLKQQQQSSIKSTVESSLSVSPPSSSSEIASISSPIIPPLLKTPSVKLFRSRILLGYSLKLLLCSCERGKVIRNC*

>AL1G12810

MGSEQNDGTSFSPSEPKLCVKGCGFFGSPSNMNLCSKCYRDIRATEEQTASAKAAVEKSLNPNKPKIQPQQSQEITQGVLESGSSSSSTTSGGDLAAAPLDPPKSTATRCLSCNKKVGVTGFKCKCGSTFCGTHRYPETHECEFDFKGVAREAIAKANPVVKADKVERI*

>AL1G45860

MVLEMDHDKTGCQSPPEGPKLCINNCGFFGSAATMNMCSKCHKDMLFEQEQGAKFASAVSGTSSSSNLIKETITAALVDVETKSVEPMTVSVQPSSVQVVAEVVAPEEAAKPKGPSRCTTCNKRVGLTGFKCRCGNLFCGTHRYADIHDCSFNYHAAAQEAIAKANPVVKAEKLDKI*

>AL3G13060

MAQRTEKEETEFKVLDTLTTTTTTTLCTNNCGVTANPATNNMCQKCFNASLVSAAAGVVESGSILKRSARSVNLRSSPAKVVIRTREIDAVKRDQQIVNRCSGCRKKVGLTGFRCRCGELFCSEHRYSDRHDCSYDYKTAGREAIARENPVVKAAKMVKV*

>AL4G12660

MAEEHRLQEPRLCAKNCGFFGSTATQNLCSKCFRDVQHQEQNSSTAKHALNQTLAAVSTGGGTASSSVSPPPQADSKEIVEANSKKRAAAEEEEAAPSQDPKRCLTCRRRVGITGFRCRCGFVFCGTHRYAELHECSFDFKRIGKDKIAKANPIVKADKLEKI*

>AL4G21740

MAEEHRCETPEGHRLCVNNCGFFGSSATMNLCSNCYGDLCLKQQQQASIKSTVESSRSPVIAPVLENYAAELEIPTTKTEEKKPVQNPTEQPPPPPQRPNRCTVCRKRVGLTGFMCRCGTTFCGSHRYPEVHGCTFDFKSAGREEIAKANPLVIAAKLQKI*

>AL5G22730

MAEEHRCQTPESNRLCVNNCGFLGSSATMNLCSNCYGDLCLKQQQQSSIKSTVESSLSVSPPSSSSEIASISSPIIPPLLKTPSVKLEVPEKKPVNSPPEQNQQQRPNRCTTCRKRVGLTGFKCRCGTMYCGVHRYPEIHGCSYDFKSAGREEIAKANPLVKAAKLQKI*

>AL5G27260

MAEEHRLQEPRLCAKNCGFFGSTATQNLCSKCFRDVQHQEQNSSTAKHALNQTLAAVSTGGGTASSSVSPPPQADSKEIVEANSKKRAAAEEEEAAPSQDPKRCLTCRRRVGITGFRCRCGFVFCGTHRYAELHECSFDFKRIGKDKIAKANPIVKADKLEKI*

>AL6G35210

MGSEQNNSTSFPPTEPKLCDNGCGFFGSPSNMNLCSKCYRSLRAEEDQTAVAKAAVKKSLKLPSCSIIAPGQKHPLEIKPAHLETVVVTAEPSSVPVATEQDEAEPSRPVRPNRCFSCNKKVGVMGFKCKCGSTFCGNHRYPEKHECSFDFKEVGRDAIAKANPLVKADKVQRI*

>AL7G16740

MKPNRVSPRGDLNARLLLRGCGLYGTRKNNNLCSLCYKHSVLQHSTALRFEPKTEQSQCCPLTSSPVAEEEPVRKRRCGICKRKVGMLAFNCRCGHMFCGSHRYPEEHSCPFDYKQSGRLALATQLPLNRADKLQRF*

>AL7G19400

MGSEQNDSTSFTQSQASEPKLCAKGCGFFGSPSNMDLCSKCYRSICAEEAQTAVVKAAVEKSFKPSPLPLPSRSLFIAEPAVVKPEPEKAVVVVSAEPSSATVPEEREPSRPARPTNRCLCCNKKVGIMGFKCKCGSTFCGDHRYPEIHDCSFDFKETGRGEIAKANPVVKADKLQRF*

>AL7G30220

MTGEPSLCIRGCGFFSTSQTKNLCSKCYNDFLKDESARYLDTFNINPETAAEAAEEVTAEEETAVEAVVVKKKDKKSSSRCNACKKKVGLLGFHCRCGHMFCGSHRYPEEHSCPSDYKSAAIDVLAKQNPIVKADKLFRL*

>ATR_00022G03270

MAQREKDVTDCEVPETHQNRFGEAGILTPTPCPSVSGAGDKRSGCVPSSERAGERVLSVPAFSSEKISRKRPEESPENPTRYPNRCQSCRKRVGLTGFRCRCGDLFCGEHRYSDRHVCSFDYKAVGKEAIARENPVVKAAKIIKI*

>ATR_00022G03350

MEHDETGCEAREGPILCVNNCGFFGSAATMNMCSKCHKDLILKQEQAKLAASSIGSIVNGNNNSKDPIIANAADVVVGPIENKVVTSQPSGLVGSSSEEGGGEVKAKEGPNRCTACRKRVGLMGFNCKCGSLFCGVHRYSDKHNCPFDYRSAARNAIAKANPVVKAEKLDKI*

>ATR_00040G01200

MAEESWNRGTEGCQAPEGHRLCANNCGFFGSPATLNLCSKCYRDFCLKEDQANSAKAAVEKSLNPNNDPSVSSSIVSASVPELLDNRQKPATVSSNSSADSNASVLSSSSSLQSGPQTTAPNRCSCCRRRVGLTGFKCRCGMTFCSAHRYPEQHACSFDFKAAGREAIARANPVVKAEKLDKI*

>ATR_00041G00040

MPEKSWESCRPSENPRLCANNCGFFGSPATLNLCSKCYKDYRLKEEQAAAAKAAVEKSLAGNNPTPAAETEILAKDCQDAEIDGSAPLPSRLSSSPESDPPKNSEDRQSKIVRLGGPASLPSTSSSLPEASRDRISESASCHPSENGGHCTNNCGFFGSPATLNLCSRCYKDVRLKEEQAAAAKATVEKSLVETTPLRRPTLKAPPKIAKTR*

>ATR_00078G01580

MAQESKKRDAEDMGHEPPKGPIFCANKCGFFGSASTNNLCSKCYKDFYLKQLKEDENITDKVIPCLNNEPNAKVDAANLQNSEDGKKNKGVVVDGTSSEEPQKQAPNRCRLCRKRVGLAGFRCRCGGLFCSTHRYSDKHNCSFDYRASGQDAIAKANPVVKAEKVEKI*

>BR01G29240

MNSEQNNSTSFPPTEPELCDNRCGFFVSSNMNLCSKCYRILRAEEDLTAVAKSAVKNSLKLPSCSLIITPEQKQPLETKPASVVVTAEPSSVPIATGQEEAEPSKPARTNRCFSCNKNVRSLSVNYNQHILILLWCCLYKREEIPH*

>BR01G36430

MAQRTEKEETEFKVLETLTTTTPTLCSNNCGVTANPATNNMCQKCFNASVAAAGVDSTSILKRSARSVNLRSTPAKVVIRPREIDPVKRDQQTINRCSGCRKKVGLTGFRCRCGDLFCAEHRYSDRHDCSYDYKTAGREAIARENPVVKAAKMVKV*

>BR02G01480

MAEEHRCQTPEGPRLCANNCGFLGSSATMNLCSNCYGDLCLKQQQASMKSTVESSLSAVSPPSSEIGSMQSTVESSLSDVSPPSPETISISSPMIQPLVRNPSAELEVTATKTVTPPPEQQQKRPNRCTTCRKRVGLTGFKCRCGTTFCGAHRYPEVHGCTFDFKSAGREEIAKANPLVQAAKLQKI*

>BR02G26880

MSSEQNNSTSFPPTEPNLCDNGCGFFGSPSNMNLCSKCYRSLRAEEDHTAVAKAAVKNSLKLPSCSLIITPEQKQPLETKPASVVVTAEPSSVPIATGQEEAEPSKPARTNRCFSCNKKVGVMGFKCKCGSTFCGSHRYPEKHECSFDFKEVGRDAISKANPVIKADKVERI*

>BR03G17930

MAEEHPCQTPEGHRLCVNNCGFFGSSATMNLCSNCYGDLCLKNQQQASMKSTVESSLSAASPPSSPEIQSISTSTIAPVVQIHAAEIQIEKQIQQQRPNRCTVCRKRVGLTGFMCRCGTTFCGTHRYPEVHGCTFDFKSAGREEIAKANPLVVAAKLQKI*

>BR03G23530

MSEEHRLQEPRLCANDCGFFGNTATQNLCSKCFRDLKHEQENSSTAKNALKQTLAACVASSSSVSPPPPPPPSDLKEVNTENPEKRAAASEPEEEELKPPQDPKRCLTCRRRVGITGFRCRCGFVFCGTHRYAEQHECTFDFKRVGKEKIAKANPIVKADKLEKI*

>BR03G33060

MAQRTEKEETEFKVVLETLPTTTTPTLCSNNCGVTASPSTNNMCQKCFNASVSGIDPTPIVKRSARSVSLRSTPARAVIRPRETDPVKRDHHQQTINRCSGCRKKVGLTGFRCRCGDLFCAEHRYSDRHECSYDYKTAGREAIARENPVVKAAKMVKLS*

>BR03G46640

MYQNLMNICVSAGNLEAHYLRGMQEYFQNENMAAGLELLEIAAQGSYDNAIYLYGIIMLCRGDPDIRKPQLDSLGWRENKARSDACWQSIKTSLHGVMVKRGRNLTVPTGRMGSEQNDSTSFTQSSEPKLCANGCGFFGSPSNMDLCSKCYRDICAEEAQTAVAKAAVEKSFKPSPSPPPPTLFIAEPDVAKPEKEKAVATVLVVAEPSSATGEATVPEQNEPPSKPARPNRCLCCNKKVGILGFKCKCGSTFCGEHRYPERHDCSFDFKEAGRGEIAKANPVIKADKLQRF*

>BR03G48490

MMHANETEALPCAGGCGLFGTRTNNNLCSLCYKKSVLEQLATLKLESKPEPSTVPTRSPTVAVQEPVRKHRCETCHRKIGMTGFSCRCGHMFCGSHRYPEEHSCPFDYKQSGRLALAKQLPLTRAEKLHRF*

>BR03G55940

MESREIVPGIFEKKKKKGTTMNSEQNNSTSFPPTEPELCDNRCAEPSSAPIATGQEEAEPSKPARTNRCFSCNKKAGVMGFKCKCGSTFCRSHRCPEKHECSFNFKEVGRDVIAKANHVIKEDKVERI*

>BR04G08650

MESREIVPGIFEKKKKKKGTTMNSEQNNSTSFPPTEPELCDNRCAEPSSAPIATGQEEAEPSKPARTNRCFSCNKKAGVMGFKCKCGSTFCRSHRCPEKHECSFNFKEVGRDVIAKANHVIKEDKVERI*

>BR05G08700

MAEEHRCQTPEGHRLCVNNCGFSGSSATMNLCSNCYGDLCLNQQQQQASTKSTVESSLSAASPPSSLEIESISSSSSTAEKQIPLIQTSTEQKQPPQRPNRCTVCRKRVGLTGFMCRCGTTFCGTHRYPEVHGCSYDFKSAGREEIAKANPLVIAAKLQKI*

>BR05G30250

MAQRTEKEETEFKVLETLTTPATATTTLCSNNCGVTANPATNNMCQKCFNASIAAAGVDSGSTSKRASRSVNLRPSPAKVVIRPREIDPVKRDQQTVNRCSGCRKKVGLTGFRCRCGDLFCSEHRYSDRHDCSYDYKTAGREAIARENPVVKAAKMVKV*

>BR06G02490

MDHDKTGCQSPPEGPKLCINNCGFFGSAATMNMCSKCHKTILFQQEQGAKLASAVSGSPSNILKETFTADLVDAGTKSIEPVAVSVQAVAEVVAPEEAAAKPKEGPSRCTTCNKRVGLTGFKCRCGDLFCGTHRYADVHNCSFDYHVAAQEAIAKANPVVKADKLDKI*

>BR06G09700

MAEEHRCQTPEGHRLCANNCGFLGSSATMNLCSNCYGDLCLKQQQQGSSSLSAVSPPSPVITSISTPMIQPLVQNPSAELEVPAKNVPVTVTATEQPQKRPNRCTTCRKRVGLTGFKCRCGTTFCGAHRYPEVHGCTFDFKSAGREEIAKANPLVKTAKLQKI*

>BR08G02690

MDHDETGCQSPPEGPKLCINNCGFFGSSATMNMCSKCHKAILFQQEQGARFASAVSGCTSSSSNILKETFAATALVDAETKSVEPVAVSLQPSSVQFVVAEVVAPEASAAKPKEGPSRCATCNKRVGLTGFKCRCGDLFCGTHRYADIHNCSFNYHAAAQEAIAKANPVVKAEKLDKI*

>BR08G02710

MRFHNEHVFQVSKPKEGPTRCATCKKRVGLTGFKCRSGDLFCGTHRYADIHNCSFNYHVAVQEPIAKANPVVKAEKLDKI*

>BR08G20520

MTGEPSLCINGCGFFSTPQTKNLCSKCYNSFLKDESARYLDTIRDHRTTATVVEKSEEAVVVVRNKKRSRCNACNKKVGLLGFECRCGHVFCGSHRHPEEHSCLSDYKSAAITELTIQNPVIKPDKLYRI*

>BR09G10970

MNLCSKCYHDLRVTQEASFEPETSLESALASSSSSSESSGTAAQTSAKTRRCLSCNKKVGLMGFKCKCGSTFCGDHRYPENHECEFDFRGQGRDAISKANPLVKGEKVKRF*

>BR09G14970

MNPMFYFLTLTAVLAATANAGGPVLDSKGHHIFSGSYYVIPRIFGAAGGGLTLVPRGDKQCPLYVGHETLEVKMGIPVKFSSWMSRVGFVPESENLNIMMDAKATTCSQSNYWWVAPSDKDRKTWFIAAGPKPKTKEDRSMTSFQIKKTGDSLKGYKIVYCPKAEPSSAPIATGQEEAEPSKPARTNRCFSCNKKAGVMGFKCKCGSTFCRSHRCPEKHECSFNFKEVGRDVIAKANHVIKEDKVERI*

>BR09G24860

MSSEQNNSTSFPPTEPKLCDNGCGFFGSPSNMNLCSKCYRSLRAEEDQTAVAKAAVEKSLKLPSCSLITAPEPKQPLETKPASVETVVIAETSSVPPVATGQDEGEPSKPARPNRCFSCNKKVGVMGFKCKCGSTFCGSHRYPEKHECSFDFKEVGRGAIAKANPVVKADKVQRI*

>BR09G34170

MAEEHRCQTPEGPRLCANNCGFLGSSATMNLCSNCYGDLCLKQQQQASIKTTVESSLSAVSPPSTEIASVSSPVISPLVQNPSAELEVTKKNVPATLTPPPSQTTTEEKQLKRPNRCTTCRKRVGLTGFKCRCGTTFCGVHRYPEVHGCTFDFKSAGREEIAKANPLVKAAKLQKI*

>BR09G49630

MESEQNDSFSPSEPKLCVNGCGFFGSPSNMNLCSKCYRDIRATEEQAASAKAAVDKSLNPNKPHTKPPQQSVETAPEPSSSASGGDSSDPPRATRCLSCNKKVGVTGFKCRCGSTFCGAHRYPESHDCEFDFKGAAREAIAKANPVVKADKVEKI*

>BR10G04460

MNSEQNNSTSFPPTEPELCDNRCAEPSSAPIATGQEEAEPSKPARTNRCFSCNKKAGVMGFKCKCGSTFCRSHRCPEKHECSFNFKEVGRDVIAKANHVIKADKVVRI*

>BV0G17280

MHNVQDPSQLCAKGCGFFASPSLRNMCSSCYRDYLKETLLKMERKSSHNDSKQQSVVDSSDEKDANTTTSTCTRCDLCRRRVGLMGFKCRCGGMYCGSHRYGKEHSCAIDNIRKALQREALSKQLNASTCKADKLNCRI*

>BV0G43400

MANHDGMAATPTLCRNSCGFFGRPDQRDLCSKCFRELMKTEATSQGTPIVPEAPNVPTLQPAVTPPEPTIKEPVVAAPVVKKSDRHRCSVCRKKLRLAQQFECMCQETFCGEHRYADSHSCSFDYMKRHQERLRKDNPEVIAAKLDKI*

>BV3G15960

MDHDETGCQTPDRPVLCVNNCGFFGRAGTRNMCSKCHRETVLKEEQTRLAASSFDNIVNGTSKGKEAAAAASVDLKSEVVVTKSVNVKVSCDSGVERPSEAKKAGPTRCMTCRKRVGLTGFSCRCGNMFCSTHRYSDKHDCPFDYQAAAQSAIAKANPIIKPDKLDKI*

>BV4G12570

MAQKREKDETLELKVPAETPLSLSCPDNCKKITSSSSSTSNTTRPDMARIDPVSSEPARPRSEREDHQQQQEHHETTSLLKDTQQNAASSDLLLSSVSTNPVKKAVNRCSGCGKRVGLTGFRCRCGDLFCADHRYSDRHDCSYDYKTAGREAIARENPVVRAAKILKV*

>BV4G12610

MDHDETGCQAPPEGPILCVNNCGFFGSVATMNMCSKCYKDAVLKQEQAKLAASSIENIVNGSSNGNGKEPVVIGTTMDMQVKLIAPKAITIPESSVPSSLGEGGDAKPKQGPSRCTTCKKRVGLTGFNCRCGNLFCATHRYSDKHECPFDYRTAARDAIAKANPVIKAEKLDKI*

>BV5G08690

MNCETRDTTINCANGCGFFGTAATMNLCSKCYRDHRLKEEQTAAVGKSMTSILTSQPEHKEERESGKSVEIVAEAATMEVAQHSLKKGRCGSCNKKVGVVGFKCKCGVTFCGSHRYPEEHQCKFDYKVVGREELAKANPQIVADKLQRI*

>BV7G12310

MAEEHGYQAAEGHRLCANNCGFFGSSATLNLCSKCYRDVHMKEEQDAIAKAALDSTNPHPHPNPETLSGLGLDAGSSTLVEPNKLLGSTEEGWNSAVEIVSIAEVQPGFVVNLITRPEPVSVISAQPSRCLTCRKRVGLTGFKCRCGLMFCGSHRYPEQHGCTFDYKTVGKDAIAKANPVIKARKLDKI*

>CL01G03270

MGSEQNDGTSYQPSEPKLCANNCGFFGSAGTENLCSKCYRDSRIKEEQAASAKAAMEKSLQSKILKPAEGSSPVVFSDSTVDSASPSSSTGISLRSSNKSPSPDVPNRCRSCNKKVGLMGFKCKCERTFCGIHRYPEKHDCCYDFKSIGREEIATANPVVKADKVERF*

>CL02G22250

MAEEHRCQAPKLCVNNCGFFGSPATQDLCSKCYRDLQLKEQQSSSAKLALNQTLTSAASSSFADAALLNPKPASSEANQPLAETRTEVVPTPAATQQQPSRCTTCRRRVGLTGFQCRCGMVYCGTHRYPEQHGCEFDFKRMGKEQIAKANPVVKGEKLQKI*

>CL02G23340

MCSKCHKDMMLKQDQAKLAASSIENFVNGSSSSSKEVPESVTDGAISVEPKTIQSHALPAMGSVEGEKPNEGPKRCNSCNKRVGLTGFNCRCGNVFCAVHRYSDKHDCPFDYRMAAQNAISKANPVVKAQKLDKI*

>CL03G12960

MLKEDQAKLSASTLGSIMNGSSSSNQKETFVTASVDEPVSLVEPKITSMQASPVVVSDGSSGVKPKDRPRRCNSCNKRVGLMGFDCRCGNIFCAIHRYSDKHDCPFDYRTSAQDAIAKANPIVKAEKLDKI*

>CL04G09860

MAEEHRCQAHEGHRLCANNCGFFGSPATMDLCSKCYRDFRLKEQEQASTKSTIEAALSASSSPSPSSSPIDPPPVPPVVELSLPDSTTDLTIPPLAAVGSEAANQPNPTPNPNPNPNRCSSCRKKVGLTGFKCRCGTTFCGAHRYPEKHGCTFDFKSIGXXXSEERRSLERTPSSKPINWKGFERFLRPLHSSMATTVANFTVAHHPIIFAAVETLSAVRSPFNVAVDDDAALSLSISLQYKNNEDGFPCKKRISIFFLLVSELKVSSLLPVNRVRLSSLLN*

>CL05G08570

MAASGSSKNDADAPLCANNCGFYGNPNNRNLCSVCYAAFLKGTAEDSERRENSKNQIKDEEPNLGTRQSPSASHSPETCENNPALIPKNRCKICRKKVGLLGFNCRCGGPFCGKHRYPEEHSCDFDHKAIGRNILAKQIVECKADKLEFRV*

>CL09G03880

MEHKETGCQAPPEAPKLCANNCGFFGSAATMNMCSKCHKDFIMKQEQAKLAASSFGNIVNCGPNDHGKKAVITSPTKLEIEPAESSVIPTQASCTSSNASSTMEKVKESPKRCSSCRKRVGLTGFNCRCGSLFCAVHRYSDKHNCPFDYQAVGREAIAKANPVVKAEKLDKI*

>CL09G03890

MAQRTEKEETEFKVPETITLCVNNCGLTGNPATNNMCQKCFNATTATASMAMKFSGEKSPRSTTSRSPEKFRFVSESRRIVTAADRPKPDESAKREVNRCSGCRKRVGLTGFRCRCGDLFCAEHRYSDRHDCSFDYKAAGREAIARENPVVKAAKIVRV*

>CL10G10660

MAQRTEKEETELKVPETITLCVNNCGFTGNPTTNNMCQKCFNATTATATTTATSSSNNGISASRKLSGEKSSRSRSRSPVLMDSVRDSSRNMSMDRFKSEESAKRQVNRCSGCRKRVGLTGFRCRCGELFCAEHRYSDRHDCSFDYKAAGRDAIARENPVVKAPKIVRV*

>CM00005G05640

MGSEQNDGTSYQPSEPKLCVNNCGFFGSAGTENLCSKCYRDSRIKEEQAASAKAAMEKSLQSKILKPAEGNSPVVFSDSSVDSASSSSSTGISLGSSSKSPSPDVPNRCRTCNKKVGLMGFKCKCELTFCGIHRYPEKHNCCYDFKSAGREEIATANPVVRADKVERF*

>CM00006G09510

MATDATPCANNCGFYGNPNNRNLCSVCYMAFLKETGVKYFEQQINSKSKINLETRQSSSSGASESSETCDHNDPAPPKTKSRCEICRKKVGIIGFSCRCGGCFCGKHRYPEKHSCGFDYKEVGRTILAKQIVECKADKLEYRED*

>CM00006G09520

MATDATLCANNCGFYGNPNNQNLCSACYIAFLKETGVKYFEQQISSKSKINLETRQSSSSGSLESPETCVHNDPEPSKTKNRCEICGKRVGIVGFSCRCGGCFCGKHRYPEEHSCGFDHKEDGRMILAKQIVECKADKLEFRA*

>CM00022G04370

MDPHDEAGCQAPPEGPFLCINNCGFFGSAATMNMCSKCHKDMMLKQDQAKLAASSIENFVNGSSSGSMEVPESVTDGAVSVEPKTVQAHALPAMGSVEGEKPSEGPKRCNSCKKRVGLTGFNCRCGNVFCAVHRYSDKHDCPFDYHMAAQNAISKANPVVKAQKLDKI*

>CM00024G02400

MEHDEASCQAPPEGPFLCINNCGFFGSAATMNMCSKCHKDLMLKQEQAKLSASTFGSIMNGSSSSNQNETFATASVDDPVSLVEPKITSMQASPVVISDENSGAKPKDRPRRCNSCNKRVGLMGFDCRCGNIFCAIHRYSDKHNCPFDYRTSAQDAIAKANPIVKAEKLDKI*

>CM00047G02930

MAEEHRCQAPKLCANNCGFFGSPATRDLCSKCYRDLQLKEQQSSSAKLALNQTLAASSSSSSSSLLLNLKPEAKEQRVEEEVRTEVVRTEAAEARQQQQQQQPSRCMTCRRRVGLTGFKCRCGMVYCGSHRYPEQHGCEFDFKQMGKDQIAKANPVVKGEKLQKI*

>CM00060G01830

MAQRTEKEETEFKVPETITLCVNNCGLTGNPATNNMCQKCFNATTTTASMAMKFSGEKSPRSTISRSPEKFRFVSESRRIITAADRPKPDESAKREVNRCSGCRKRVGLTGFRCRCGDLFCAEHRYSDRHDCSFDYKAAGREAIARENPVVKAAKIVRV*

>CM00060G01840

MEHKETGCQAPPEAPKLCANNCGFFGSAATMNMCSKCHKDFIMKQEQAKLAASSFGNIVNCGPNDHGKKAVVTSPTKLEIEPAESSIIPTQATCTSSNASSTVEKVKESPNRCSSCRKRVGLTGFNCRCGSLFCAVHRYSDKHNCSFDYQAVGREAIAKANPVVKAEKLDKI*

>CM00064G00490

MAQRTEKEETELKVPETITLCVNNCGFTGNPTTNNMCQKCFNATTATATTTATSSSNTGISSSRKLSGEKSLRSRSRSPVLMDSVRDNSRNMSMDRFKSEESAKRQVNRCSGCRKRVGLTGFRCRCGELFCAEHRYSDRHDCSFDYKAAGRDAIARENPVVKAPKIVRV*

>CM00068G00850

MAEEHRCQAPEGHRLCANNCGFFGSPATMDLCSKCYRDFRLKEQEQASTKSTIQAALSASSSPSPSSSPIDPPPVPPVVALSLPDTTTDLTIPPLAAVGSDVTNPSPSPSPNRCSSCRKKVGLTGFKCRCGTTFCGAHRYPEKHGCTFDFKSIGREEIARANPVVKADKLERI*

>CP00005G00390

MAEEHRCRAPEGHRLCANNCGFFGSPATMNLCSKCYRDFRLKEQQQASIKSTVESSLAASASSPPLSFPAIGSVSSPAAPVLPDVGGDVPNPAMEVVAEDRQPKEATKQQPNRCLVCRKRVGLTGFACRCGITFCGVHRYPEKHGCAFDFKKLGREEIAKANPLVKAEKLEKI*

>CP00029G00600

MGSEQNDGTSVPPSEPKLCANGCGFFGTAANMNLCSKCYRDLRVKEEQAASAKAAVEESLNPKPPKQQEQQPIAASVVSVAASATAAASSSGVLTVGGHQVEPKVANRCSSCNKKVGLTGFKCKCGSTFCGVHRYPENHQCIFDFKGAGRDAIAKANPVVKADKVERI*

>CP00032G00890

MAEEHRFQAPKLCANNCGFFGSPATQNLCSKCFGDLQLKEQQSSSAKQSLSKALISAPSSSSSSSSTFSPPSPIDVSSPSIQVLETAKKHAAVGEREERSEAAARVEEQEQRQRPGRCTACGKRVGLTGFKCRCGLVLCGTHRHPEQHGCSFDFKGMGKQQIAVANPIVNAEKLEKI*

>CP00107G00190

MCQRGCGFYGSVENQNLCSKCYKEFLKQRRKRKSSGASRKNSAAHLILLYRLVCHSCNRKVGLTGFACRCGQVFCGMHRYPEKHSCRFDYKIAGRQVLAKQNPVCKGDKLHHRI*

>CP02168G00010

MAQKTEKEETEFKVPETLTLCVNNCGVTGNPATNNMCQNCFNATTATSSSSTITTAATTAGGVGVGVGGGNGGSGILKFSSCEKTARSTSSSRSTDRSDSFTTTSRSISLDRREGDVSAEKRIVNRCSGCRRKVGLTGFRCRCGELFCADHRYSDRHDCNFDYKAAGREAIARENPVVKAAKIVRV*

>CR09G01740

MEREQASSPQLCEKGCGFFANVGCGGMCSKCHREEARQHANAQATSSQPKPVEVAASRPVHESFPQPAAPSEAVASPVAEASTSSGDASPSATKSANPSRCLCCKKKVGLTGFKCKCGDVFCGTHRYAESHNCPFDYKTVHKEKLASNNPVVQASKVQKI*

>CRU_001G11520

MGSEQNDGTSFCPSEPKLCVNGCGFFGSPSNMNLCSKCYRDIRAKEEQAASAKAAVEKSLNPNKPKIQPQQSKDVTSGVVESGSSSSSTSGDGAAPVDPPKSTATRCLSCNKKVGVTGFKCKCGSTFCGAHRYPETHDCEFDFKGVAREAIAKSNPVVKADKVDRI*

>CRU_001G38200

MDHDKTGCQSPPEGPKLCVNNCGFFGSAATMNMCSKCHKDMLFQQEQGAKFASAVSGASSSSNIIKETITAALVDVETKSVEPMAVAVQPSSVQVVAEVVAPEEAAKPKGPSRCTTCNKRVGLTGFKCRCGDLFCGTHRYADIHDCSFNYHAAAQEAIAKANPVVKAEKLDKI*

>CRU_003G11450

MAQRTEKEETEFKVLETLTTTTAATLLCTNNCGVTANPATNNMCQKCFNASLVSAATSGVVVDTGSILKRSARSVNLRSSPAKVVIRPREMDAVKRDQQVVNRCSGCRKKVGLTGFRCRCGELFCSEHRYSDRHDCSYDYKTAGREAIARENPVVKAAKMVKV*

>CRU_004G08060

ISSFISFLSHLLFSFSIDLSLSHHSLPLYIPDPSSSFSYLFFLESNRFNQKSRRSLDLLLFFFFGNFPVMAEEHRLQEPKLCANNCGFFGSTATQNLCSKCFRDGQHAQQNSSSAKLALNQSLAAVAVSSGGGVASSSVSSSSSSSPIETDLKETNTGKRAAETEVEEEEEVVPPQDPKRCLTCRRRVGITGFRCRCGFVFCGTHRYAEKHECSFDFKRVGKDKIAKANPIVKADKLEKI*

>CRU_004G17500

MAEEHRCETPEGHRLCVNNCGFFGSSTTMNLCSNCYGDLCLKQQQQASLKSTVESSLSASPPPPPPAVIAPLLDNYAAELEIPTKAEEKKPIQASPTEDQPQQRPNRCTVCRKRVGLTGFMCRCGTTFCGSHRYPEVHGCTFDFKSAGREEIARANPLVVAAKLQKI*

>CRU_005G18130

MAEEHGCQTPESNRLCVNNCGFLGSSSTMNLCSNCYGDLCLKQQQQSSSSSSIISTVDSSSSLSVSPPSSSSSSEEIASVSSSPTPIIPLLLHTPSPKLEKKPPQTTTTEQQKPNRCTTCRKRVGLTGFKCRCGTMFCGAHRYPEIHGCTYDFKSAGREEIAKANPLVKAAKLQKI*

>CRU_006G29960

MGSEQNNSTSFPPTEPKLCDNGCGFFGSPSNMNLCSKCYRSLRAEEDQTAVAKAAVKNSLKLPSCSIIAPEQKQPLELKPAPVETVVVAAEPSSVPVVANAEQDEAEPSRPARPNRCFSCNKKVGVLGFKCKCGSTFCGNHRYPEKHECSFDFKEVGRDAIAKANPVIKADKVQRI*

>CRU_007G14770

MNANETEALLCTGGCGLYGTRKNNNLCSLFYKKSVLQHSPAFRLEPETERSQFCSPTSSPVPVKEEPVGKRRCDVCKRKVGVLGFKCRCGHMFCGSHRYPEEHSCPFDYKQSGRLALATQLPLNRADKLQRF*

>CRU_007G17560

MGSEQNDSTSFTQSQASEPKLCASGCGFFGSPSNMDLCSKCYRSLCAEETQTVAVKAAVEKSLKPSSPSLSPRPRSLFIAEPSVKPEHHPEKAVLAVAEPSAARGGEEEEKEEEAVPDENNEPSRPARPNRCLCCNKKVGIMGFKCKCGSTFCGEHRYPETHDCSFDFKEMGRGEIAKANPVVKADKLQRF*

>CRU_007G26930

MTGEPSLCIRGCGFFSTSQTKNLCSKCYNDFLKDESARYLATLNNNSKTAAEAAEEVTAAEKATAVEVKKKSSRCNSCKKKVGLLGFQCRCGHMFCGTHRYPEEHSCPSDYKAVAIHELTKQNPIVKGDKLFKL*

>CS00031G00320

MDRNNVTMCMKGCGFYGSKENKNMCSKCYDDYLKAELIAKSSKLLDAAKKSIGPTNAPNPSVLDKSWPPQWIISAAKTTNTSNAVDSRASNQTTSTIESGAGSSVKRRCEICNKKVGLIEFRCRCGHLYCGTHRYPKEHACTFDFKKFDREMLVKDNPLIRADKLEGRI*

>CS00031G00340

MTPPLCAKGCGFYGTKEHKSMCSKCYNDFLEEQVTDGVVKRPLKLMQPNPSILVFDPRSLQSPSCSSSERTTIDSAAVECSSGKTTSALEKRCEICDKKVGSIELKCRCGHLYCGTHRYPKEHACTFDFKKFDRETLVEDDPLIRADKLEGRI*

>CS00031G00350

MTPPLCTKGCGFYGTKEHKSMCSNCYNDFLEEQVTDGVVKRPLKLMQPNPSILVFDPRSLQSHSSSSSERTTIDSAAVEYSSAKTTSALEKRCEICDKKVGSIELKCRCGHLYCGTHRKFDRETLVEDNPLIRADKLEGRI*

>CS00033G00100

MYWGGENMDHDETGCQAPPEGPILCINNCGFFGSVATMNMCSKCYKDIMLKQDQAKLAASSIGSIVNETSSSNGSESVAAATVDVQASSVEPKIISVQPSCASELSESVEAKPKEGPSRCSSCKKRVGLTGFKCRCGNLYCVSHRYSDKHNCPFDYRTAARDAIIKANPVIKAEKLDKI*

>CS00040G00770

MGSEQSDGTSYTTSEPKLCVNGCAFFGTAANMGLCSKCYRDLRVKEDQAASAKAAMEKSLNLKSPKQIHQTPELETAKVAAEPFVGSSLSAAASQQLSVEQPEPQAKGPTRCLSCNKKVGLTGFKCKCGSTFCGIHRYPEKHDCTFDFKVTGRDAIARANPVVKADKLDRI*

>CS00056G00730

MAEEHRFQAQEGHRLCANNCGFFGSTATMNLCSKCYRDHCLKEQQQASIKTTVENSLSASPPSSSAPFLGSVPDPPALALPEVNGDKDADVAVVLEQQQQQPPQPNRCSVCRKRIGLTGFKCRCGTIFCGTHRYPEKHGCSFDFKKVGREEIARANPLIKAEKLEKI*

>CS00092G00100

MNTCEVEKMESHDETGCQAPEGPILCVNNCGFFGSAATMNMCSKCHKAMILNQEQAQLAASSIGSIVHGSSGGTGKEPIVAGSVDLQVGPVEIKKFSMEPSSGSSFGFAGVKAKEGPKRCTTCNKRVGLTGFNCRCGNLFCAVHRYSDKHGCPFDYRTAAREAIAKANPVVKAEKLDKI*

>CS00207G00150

MEQESQKRKLEETSHETQNTPIYCANNCGFFGSANTNNLCSKCYKDYLLKQSKNPNPSPIEEEKNKNKTNIEQNEANPVGGVVENRDPAAQVVVVDEGASSENPEKRPANRCSFCRKRIGLTGFKCRCEQTFCSLHRYSDKHNCVFDYKSAGQDAIAKANPVVKADKIEKI*

>CS05856G00010

MDRNNVTMCIKGCGFYGSKENKNMCSKCYDDYLKAELIAKSSKLLDAAKKPIGPTNAPNPSVLDKSWPPQWIISAAKTTNNSNAVDSRTIESGAAGSSVKRRCEICNKKVGLIEFKCRCGHLYCGTHRYPKEHACTFDFKKFDREMLVKDNPLIRADKLEGRI*

>CS09133G00010

MAQKTEKEETEFKVPETLTLCVNNCGFTGNPATNNMCQKCFNATATTTASAVAGSSTGGGGGGGSGVAIIKFSSEKSLRSRPIIRSGSSDAAGTTGQNQELTDRREKEANVEKRVVNRCSGCRRKVGLTGFRCRCGELFCGEHRYSDRHDCSYDYKSAGRDAIARENPVIKAAKIVRV*

>EG0001G20240

MEFPNGMGWQSTKGCILCINCGFFGSAAMGNMCSKCHKDMLQKREQAELAASSSTTSIGAASSGESGKEHAVAGARDVHSGAVDVEASSITSLPESTSTGNSAALLFRNLRMNVKEGPNRCTACRKRVGLTGFNCRCGNLFCAAHRHADEHDCSIDYWTANRAALAKINPIVKAEKLGKI*

>EG0002G32530

MAEEHRCQAPRLCANNCGFFGSPATQDFCSKCYRDLQLKEQQSSNAKLAFNQTLSSSSSSSPSSSSSSSSSSIVEPPAAAVDCKVAAVALEAKPEGAVAAGEAPMPAVSNRCSTCRRRVGLTGFKCRCGMTFCGSHRYPEKHDCGFDYKGLGREQISKANPIVKAEKLDKI*

>EG0003G29380

MTVNTSTPTMSKKKNRCTLCNKHVGLLGFECGCGDLFCRAHRRTRLRCRFQDGGEAEIVRGESSLQG*

>EG0003G29390

MKTNADFTGEPPLCAAGCGFYGSREHHDLCSKCYAAFLKDQVAKSTAAAAAKAASGRQPSNSASRVAASLDDMAALFSDLTVDTSTPMTSKKKNRCAACSKRVGLLGFECRCGNVFCGAHRYPEEHGCDVDFKTAARRRLSKENPVCKADKMDFRI*

>EG0004G15980

MGSERDDSVPVTEARLCANNCGFWGNPATMGLCSKCYKDLCLREQRAAAAKAAMEKSLAPDAAPAPQETSTRAAASPSQAMPGVGASSSEFPPTSGEPKSKATNRCEACNRKVGLTGFNCKCGSTFCGAHRYPESHRCEFNFKKSGQDAIAKANPLIKADKVDKI*

>EG0007G27350

MAQRTEKEETEFKVPEALTLCVNNCGFAGNPATNNMCQKCFNASSSSSSSSTTTSTTASTASSAPTTSSSAGLTGGPSVLKFSAERIHRSGPFSRSPDRSGLAMGMIRRDTGASPPSSTESNSARREVNRCSGCRKRVGLTGFRCRCGELFCAEHRYSDRHDCSYDYKAAGREAIARENPVVKAAKIVRV*

>EG0008G39620

MSLLLICAVAYVAGSPRAGPGCILYANQQTTLSLSLSRLQARTARHGRILQICDGFPVRIKIRSLFGGDRNSWRLCCEFRFGCEFGSCPIGFSGLSVGIANWVVVRMDEQSQKRKLRDVDCEQSSSSGDPVLCANDCGFFGNPSTNNLCSRCYYEHLLKQPKDAVDSTAAGSEGAGVGGGDAGDGSCCVEEAPAGDVANWVEAAAISEKQETTPADHSSQPSSSNPVLCANSCGFFGNPTTNNLCSKCHCEYLLKQSKESSGSAAVAEDTKDVGAVDGSSHAKETPVAATANRVEQVAALENQEMRPVNRCGFCTKRVGLTGFKCKCGETFCSVHRYSEKHDCVFNYKTAGRDAIAKANPVVKADKIEKI*

>EG0010G06960

MEHDETGCQAPPEGPILCINNCGFFGSPATMNMCSKCHKDMLLKQEQAKLAASSIAAAENGSSSTTASEPVFAASVDTQLNTVEPKTIPKQPACDSVTVATVEKPKEGPTRCSSCNKRVGLTGFSCRCGNIFCGTHRYSDKHDCPFDYRSAGRNAIAKANPVIKAEKLDKI*

>EG0011G14900

MESHDETGCQAPKGPILCINNCGFFGSAATANMCSKCHKDVILKQEQAQAAASSIESIVNRSSNENGKGPVATEKLDLQAGLVDAMVISTEPSAGSSSSKDVDMKVSDGPKRCTTCQRRVGLTGFNCKCGNLFCSTHRYSDIHGCPFDYRTAARDAIAKANPVVRADKLDKI*

>EG0011G14910

MRKGRKGNNRPFSLSIVYSIVEAIERERRTLLHKPLSLSLSSPVSVDSRWSRVPRIVSIAYAESRREFGRGFDFVERDQQNQEIRTMESHNETGCQPPKGPILCINNCGFFGSAATANMCSKCHKDVMLKQEQAQAAASSLESIVNGSSSENGKEPAASETLDLRAGLVDTKFIPTELSAGSSSNRDVETKVNEGPKRCACCHKRVGLTGFNCKCGSLFCAVHRYSDKHDCPFDYRTAARNAIAKANPVVKADKLDKI*

>FV0G51630

MDSHDECQAPDCSILCINKCGFFGRATTMNMCSKCYKDMLLKQEQADLAATSIGSLVNGNVGIRPVVATAVNVQAGEVEAVAVSTEPSTGSSSSKVSEEVKEITGPKRCTSCRKRVGLTGFNCKCGNTFCSTHRYSDKHDCPFDYRTAGQDAIAKANPVVKAEKLDKI*

>FV1G19700

MEQESQKRKLDETGIEPSNAPILCVNNCGYYGSAKTNDLCSKCYKEFLLTQSKGAVEGTVIVENKVGEGGANVVNKSHVEQVVEEVRQSQVEEGTTSENPEKRPANRCSFCRKRVGLTGFKCRCGDTFCSLHRYSNSHNCMFDYKSAGQDAIAKANPVIKADKIDKI*

>FV1G23590

MAEEHRCQALEGHRLCANSCRFFGSPATINLCSKCYRDFCLKEQQEASIKSTVEASLSASVSPSVSSSSSSSLVIDFRSLPPPPLLTLPEVTEAEDVPTVPEVAVAAVSQPNQCDVCRKRVGLTEFKCKCGTTFCGIYRYPEKHACSFDFKTVGKEEIARSNPLIKAEKLEKI*

>FV2G44220

MAEEHRCQAQKLCVNNCGFFGSPTTQNLCSKCYRDLQHKEQQALSLNQTIISAAAAAATSSSSSAFFPSSPVSVATQQSERVVVDAKVEEEEKKEEVAAVPAAASQANRCMACKRRVGLTGFKCRCGMTFCGTHRYPEQHACGFDFRSMGREQIAKANPVIRGEKLQRI*

>FV3G42390

MDHEETGCQAAPEGPILCVNSCGFFGSAATMNMCSKCHKDMMMKQEQAKLAASSIGSIVNGTSSSHGKEPVAANPADPKTIFMNSAVAFRSEYDSPSPFSFGPSESAVAKPDPESFTFGSGKNDEPKPEGPKRCTTCNKRVGLTGFNCRCGDLFCAVHRYSDKHDCPYDYRTAGRDAIAKANPVVKAEKLDKI*

>FV5G30030

MNLHDESSGCQAPPAGPIICIKHCGFYGAAATMNMCSKCYKDSVMKQEQPKLAASSTGIAINGKPKEPIVSADVHVNVQADPADPKSNFKNSFFSFASQYDASDPRPSSFTFGSGQNNGEVKPPEGKKRCTSCNKRVGLTGFSCRCGDLFCAVHRYPEKHECSYDYRSTGREVLAKANPAVKAEKLDKI*

>FV6G18510

MAEEHRCQAPEGHRLCANSCGFFGSPATMNLCSKCYRDFCLKEQQEASIKSTVEASLSASVSPSVSSSSSPVIDFRSLPPPPLLTLPEVAESEDVPTVPEVAVAAVSQPNRCNVCRKRVGLTGFKCKCGTTFCGIHRYPEKHACSFDFKTVGKVEIARSNPLIKAEKLEKI*

>FV6G20940

MAEEHRCQAPEDFCRLKEQQEAPIKSTIEVSLSASVSPSVSSSSSSSPVIDFRSLPPPPLLTLQEVAEVEDVPTVPDVAITAVSQPNRCDVCQKRVGLTGFKCKCGTTFCGIHQYPKKHACSFDFKTDSTAEAHTPLTLELHRDLVLEP*

>FV7G02130

MAQRTEKEETEFKVPDTLTHCVNNCGVTGNPATNNMCQKCFNATTSTPTTVATATTSSTTAAVFKFSGEKVPRSSSFRFDAPPSETIRKAAASAESTRSEESSMRRVVNRCSGCRRKVGLTGFRCRCGELFCSEHRYSDRHVCSYDYKAAGREAIARENPVVKAAKIVRV*

>FV7G02150

MNNNVVSRFIRVWFAWFLRYMVPDSHLCLVLGTLVSDRFLPNWDPLQQIPVDSNGFSCLIAATSGSDFSISLTPFPFPVRFALGERISELGLLCEQEYFKRMEHNETGCQAPPEAPKLCANNCGFFGSAATMNLCSKCHKDLVLKQEQAKVAAVSIESAVNATPNNWGKKPAVTLDVQAGSPDLPLISTEASSTPPPNNEEKVKETPTRCNTCRKRVGLTGFNCRCGHLFCAVHRYSDKHACQYDYRAAAQDAIAKANPVVKAEKLDKI*

>FV7G11160

MASETNMDPPLCAKGCGFYGLVAKKNMCSMCYVDDLKQELIAQSLKVYEELNNSVTNSLNSTGSTATARTRCSCCNKKLGLLGFDCKCGLVFCGQHRYPEKHSCCVDFKAAGREVLAMQNPLCKADKMESRI*

>FV7G39980

MESPSNNNCPRLCARGCGFYGSFATNSMCSKCYRDYQKEEELLAKFIAAPSAVAPVDKTLDVDSVSANKSSSTSTTSDDQPSGVTNKKNCCQNESCGKSDKTCKTSLK*

>FV7G40000

MESPSNNNCPPRCARGCGFYGNFATNSMCSKCYRDYQKEEELLAKFIAAPSAVAPVEKTLDVESVSANESPSSTSDDQPSGVTKKRNCCQNESCKKKLTLVQVMSGKCRCGGVFCDKHRLPEEHSCSVNFKETGKEVLAQQNPVCVADKLENRV*

>GM03G29662

MESHDETGCQAPERPILCINNCGFFGRAATMNMCSKCYKDMLLKQEQDNFAASSVENIVNGSSNGNGAVTTGAVDVQVEAVEVKTVSAQSSVDSSSGESLEMKAKNSPSRCATCRKRVGLTGFSCKCGNLFCAMHRYSDKHECPFDYRTVGQDAIAKANPIVKADKLDKI*

>GM03G35180

MAEEHRCQAPEGHRLCSNNCGFFGSPATMNLCSKCYRDIRLKEEEQAKTKSTIETALSGSSSATVAVASAVDSLPAPVESLPQPSVVSSPDVAAPVQANRCGACRKRVGLTGFKCRCGTTFCGTHRYPEKHACGFDFKAVGREEIARANPVIKGEKLRRI*

>GM05G04100

MGSQQKPCANNCGFFGTSEKRNLCSKCYKDLCLEEELAAMKSVLCSPAPPSPGTAGQSKPANRCGTCNKKVGLTGFACKCGSTFCGVHRYPEKHECTYDFKGEAREAISKANPVVKGDKVDRF*

>GM08G16270

MAQKTEKEETDFKVPETITLCVNNCGVTGNPATNNMCQKCFTAFTTSTATTSGAGGAGIASPATRSGISARPLKRSFPEEPSPPADPPSSDQTTPSEAKRVVNRCSGCRRKVGLTGFRCRCGELFCAEHRYSDRHDCSYDYKAAGREAIARENPVIRAAKIVKV*

>GM09G28380

MVPSLCANGCGFYGSSATKNLCSKCYIDYLKENVTKSKECETKNLSDQVFVLDQSSATSCKPCASEVSNTDDATMVDVSLTDPEGIINNKRKKRCKCCNKKVGLLGFECRCGDVFCGTHRYPEKHACHVDLKEIGRQGLVKQNPVCIGDKLKHRI*

>GM10G08280

MAEEHRCEPPEGHRLCVNNCGFFGSTATMNLCSKCYSAIRLKEQEEASTKSTIETALSSASSAKPSSSTSPPPSAVDVLMESPPPSAAEVEVAVTVTVAVASSSISINSGSVAQPNRCATCRKRVGLTGFKCRCGVTFCGAHRYPEEHACGFDFKTVGREEIARANPVIKAEKLRRI*

>GM11G14050

MFYVSFAQVSWKKMDHDKTGCQAPPEGPILCINNCGFFGSAATMNMCSKCHKDMLLKQEQAKLAASSIGNIMNGSSSSTEKEPVVAAAAANVDIPVIPVEPKTVSVQPLFGSGPEGSGEAKLKDGPKRCSSCNKRVGLTGFNCRCGDLFCAVHRYSDKHNCPFDYRTAARDAIAKANPVVKAEKLDKI*

>GM11G21472

MAEEHRCETPEGHELCVNNCGFLGSTATMHLCSKCYSSIRLKEQEEVSTKFTIEIALSSSSSTKPVDVLIESESAPPFAAEVEVAITIAVVSSSISISSGSVAQPNWCATCRKRVGLTGFKCRCGVTFCRAHRYPGKHACGFD*

>GM12G06016

MDHDKTGCQAPPEGPILCINNCGFFGSAATMNMCSKCHKDILLKQEQAKLAASSIGNIMNGSSSSTEKEPVVAAAANIDIPVIPVEPKTVSVQPLFGSGPEGSVEAKPKDGPKRCSSCNKRVGLTGFNCRCGDLFCAVHRYSDKHNCPFDYRTAAQDAIAKANPVVKAEKLDKI*

>GM12G30920

MAEEHRCQAPRFCANNCGFFGSPATQNMCSKCYRDFQLKEQQSSNAKMVLNQSLVPSPPPAVISQPSSSSSAAVDPSSAVVDDAPRESEEVKAPQQNRCMTCRRRVGLTGFKCRCGMMLCGTHRYPEQHACEFDFKGMGREQIAKANPVVKGEKLDKI*

>GM12G36716

MAQKAEKNDTDFKVPETIITPCTTTTATSITEPSRFFDATTPATSSRSPKRSLPLDDESQTDQTASSEPKRVVSRCSCCRRRVGLTGFRCRCGDLFCAEHRYSDRHDCSYDYKAAGREAIARENPVVKAAKIVKV*

>GM13G21960

MAEEHRCETPEGHLLCANNCGFSGSTATMNLCSKCYGAIRLKEQEEASTKSTIETALSSSSLKPSFSTSPPTLVDVLIESPPPSLAEVAVTVAVEASSSISTSSGSVAQPNRCATCRKRVGLTGFKCRCGLTFCGAHRYSEKHACGFDFKTVGREEIARANPVIKAEKLRRI*

>GM13G27410

MAQKAQKNDTEFKVPEPIINPCTTTTTTTTSISEPSRFFDATTPATSSRSPKRSLPLDEESQTDQTTSSEPKRAVNRCSGCRRRVGLTGFRCRCGDLFCAEHRYSDRHDCSYDYKAAGREAIARENPVVKAAKIVKV*

>GM13G41590

MDHDKTGCQAPPEGPILCINNCGFFGSAATLSMCSKCHKDMMLKQEQAKLVASSIGNIMNGSSSSSGNEPVVATSVDVSVNSVESKIISAQPLVASGSDESDEAKPKDGPKRCSNCNKRVGLTGFNCRCGNLFCSEHRYSDKHNCPFDYRTAARDAIAKANPTVKAEKLVKI*

>GM15G03810

MDHDKTGCEAPPEGPILCITNCGFFGSAATMNMCSKCHKDMMLKQEQAGLVASSIGNIMNGSSSSSGNEPAIATSVDVSVNSIEPKIIPAQPLVASGSEESDEAKPKDGPNRCSNCNKRVGLTGFNCRCGNLFCSVHRYSDKHNCPFDYHTAARDAIAKANPAVKVEKLDKI*

>GM15G42740

MAQKTEKEETDFKVPETITLCVNNCGVTGNPATNNMCQKCFTASTATTSGAGGAGIASPATRSGVSARPQKRSFPEEPSPVADPPSSDQTTPSEAKRVVNRCSGCRRKVGLTGFRCRCGELFCAEHRYSDRHDCSYDYKAAGREAIARENPVIRAAKIVKV*

>GM16G33180

MVPSLCASGCGFYGSSANKNLCSKCYKDYLKVTKSNECETKNLNDQVFVLDQSSTSEDSNTDAMVDVTLTDAEGMNNKRKRCKCCNKKVGLLGFGCRCGDVFCGTHRYPEKHACKVDLKEIGRQGLVKQNPVCIGDKLEHKI*

>GM17G14580

MGSSERKPCANNCGFFGTSEKLNLCSKCYKDLCLEEELASMKSVLCSAKPPSPAEPSQGTEGPSKPANRCGTCNKKVGLTGFVCKCGSTFCGVHRYPEKHKCTYDFKGEAREAIAKANPVVKGDKIDRF*

>GM18G49010

MAEEHRCQAPCFYANNCSFFGSPTTQNMCSKCYCNFQLKEQQSSNAKMVLNQFLVPLPPLAVISQSLSFSTPAADLASKVVLDAPHVAEKTHRYPKKHACEFDFKKMGREQIAKANPVVKGKKLEKI*

>GM19G32500

MEPHDETGCQAPERPILCINNCGFFGRAATMNMCSKCYKDMLLKQEQDKFAASSVENIVNGSSNGNGKQAVATGAVAVQVEAVEVKIVCAQSSVDSSSGDSLEMKAKTGPSRCATCRKRVGLTGFSCKCGNLFCAMHRYSDKHDCPFDYRTVGQDAIAKANPIIKADKLDKI*

>GM19G37840

MAEEHRCQAPEGHRLCSNNCGFFGSPATMNLCSKCYRDIRLKEEEQAKTKSTIETALSGSSSATVTATAVVASSVESPSAPVESLPQPPVLISPDIAAPVQANRCGACRKRVGLTGFKCRCGTTFCGSHRYPEKHACGFDFKAVGREEIARANPVIKGEKLRRI*

>GR01G12150

MNETKPSNSALELKKKKISDMAEEHRCKAPPQCANNCGFFGNPATQNLCSQCYRHLQHLKEQGSSSAAKQAFNQALLPSFSSSSSSFSVSLAVKHEPLAETKEEVVQAEVQVHVQVRPNRCMTCKKRVGLTGFKCRCGMVFCGTHRYPENHGCSFDFKGMGKQQIAKSNPVVKGEKLQKI*

>GR02G22920

MDPLDETGRQASEGPILCVNNCGFFGSAATMNMCSKCHKAMILKQEQAQLVASSIDSIVNGSTSGNGKEPSVAAALAVQCGNFGSKIESSIDPSHMTFSGMKTKEGPNRCNACHKRVGLTGFSCKCGNIFCAAHRYSDKHNCPFDYRTAARDAIAKANPVVRAEKLDKI*

>GR02G24090

MAEEHRCQAPEGHRLCVNNCGFLGSPATMNLCSKCYRDFRLKEQQQASSAKSSISTSPSSSSTAVESVSQVPLLTLPQVKGVPPVVSAVAISPVTEQKPQQQQQQQQQQQPTRCTVCRKRVGLTGFKCKCGITFCGSHRYPENHGCTFDFKKIGREEIARANPVVKAEKIVRI*

>GR03G16780

MGSEQNEGTSFPPSEPKLCANGCGFFGTAANMNLCSKCYRDLRVGEEQAAKAKAVMEKSLSIKTKHEPVVVETFKPHVGSSSTSIEQQQPAVVAVNQQPEPKAANRCFICRKKVGLTGFKCRCENTFCGEHRYPEKHECSFDFKGIGRDAIAKANPVVKADKVERF*

>GR04G03620

MAQRTEKEETEFKVPETLTLCVNDCGVIGNPATNNMCQNCFNATTPKSTAAVVSSSTGGASSGGVSILNQRSTTSRSINKRSDLSPPKTTTFVRSSGSRYDPEPGTEKKVVNRCSGCRKRVGLTGFRCRCGELFCADHRYSDRHDCSYDYKTVGREAIARENPVVKAAKIIKV*

>GR04G17390

MAQRTEKEETEFKVPETLTLCVNNCGVTGNPATNNMCQKCFSATTAATSSSSSSTNNTATSATDDKSSRSTPTRSQDNRSDSAPPTTAATTATATATTNSPMTASNRSGYDTAEKKSVNRCSGCRKRVGLTGFRCRCGELFCSDHRYSDRHDCSYDYKAAGREAIARENPVVKAAKIIRV*

>GR06G03130

MSSSCHSLFRTILKLPIVVEAFKPHVGSSSTSIEQQLPVDVVVACSQQLEPKAANKCFICRMKVGLTGFKCRCENTFCGEYWYPKKHECSFDFKGVGCDAIAKANPIAKADKVERF*

>GR06G13910

MESHDETGCQAPEGPILCVNNCGFFGSAATMNMCSKCHKAMILKQEQVQLAAVSIGSIVSGCSSGSSKEPAVAAALDVQPVNIESKIVSAEPSIDPSRTTVSELETKKGPNRCSTCHKRVGLTGFNCRCENLFCAAHRYSDKHECPFDYQAAARNAISKANPVVRAEKLDKI*

>GR06G15620

MIASLRFISFVHILPSTFSFQDLADLTSSKFNFTRNLADRKILSFLKMAEEHRCQTPEGHRLCVNNCGFFGSPATMNLCSKCYRDFRLKEQQGATSIKSSLSSSSSSSSVVVESVSQVPLFTLPEFIGESPVPAVEVALVAEQRPQQQQPIRCMVCRKRVGLTGFRCKCEITFCGSHRYPENHGCTFDFKKVGREEIARANPVVKAEKLEKV*

>GR06G21130

MGSEQNQGTSFPPSEPKLCANGCGFFGTAANMNLCSKCYRDLRAGEEQAAKAKAAMEKSLSVKPKEDVVVETFKPVEKLPHAGSSSAAVEQPAVALSGDEQPEPKLSSRCFICRKKVGLTGFKCRCGSTFCGEHRYPEKHECSFDFKGTGRDAIASANPVIKADKLERF*

>GR07G24740

MNDPTSSAAAPGPSDSVVSATLISSPSKINNRCESCNRKLGLMGFTCRCGRVFCQFDRYPLEHSCNYDFKKAGRQSLAKENPVIRGDKLKSRM*

>GR07G35810

MESHDETGCQAPEGPILCINNCGFFGSAATMNMCSKCHKAMILKQEQAQLAASSIGIIVNGSSTGNSKEPAVATALDVQSGNADTKLVSTELPIDPSGTTSCGMKTKEGPNRCTKCCKRVGLTGFNCRCGNLFCAAHRYSDKHDCPFDYRAAARDAIAKANPVVRAEKLDKI*

>GR08G24090

MGSEQNEGTSFPPSEPKLCANGCGFFGTAANMNLCSKCYRDIRAGEEQAAKAKAAMEKSLSVNTKQEDVVDETVKPVLELPHVGSSSTVVEKQPAAIVSDDKPAEPKAANRCFICRKKVGLTGFKCKCGSTFCGEHRYAEKHDCSFDFKGTGRDAIAKANPVVKADKVERI*

>GR09G08650

MAEEHRCQAPQLCANNCGFFGSPTTQNLCSKCYRDLQLKEQQSSSAKQAFNHTLVPSSSSLPSSSSARSSFSASLPAKEEPSAGTKETKVVEEEEVQVTPNRCLSCKKRVGLTGFKCRCGMVFCGIHRYPEQHACAFDFKGMGKQQIAKANPLVKGEKLQKI*

>GR09G40470

MAEEHRCQAPEGHRLCVNNCGFFGSSATMNLCSKCYRDLCLKEQEASSIKSALSSSPSSSSTVVESISQVPLLALAEVNRESAVPEIAPAAEQLSQQQPNRCMVCRKRVGLTGFRCKCGVTFCGSHRYPENHGCTFDFKKVGREEIARANPLVKAEKLEKI*

>GR13G25840

MAEEHRCQAPKLCANNCGLIGSPATQNLCSKCHRDLQLKQHRSSSAKHAVNQTSIPSLSSFPSVSSSSSADKDAGSVAETKAAEVVEVEVRPKRCLSCNKRVGLTGFKCRCGMVFCGIHRYPEEHGCKFDFKAMGKQQIAQANPVVKAIKLHKI*

>LJ1G049970

MSPPSLCAPALCVNGCGFYASINNFCSKCFKDHVKEHSDHKSENEGAGNENKNLKAYIAELCDGVAAISVTDSTSMKKKRNRCEICNKKVGLTGFECRCGNLFCGTHRYPEEHSCNVDFKKIGRQVLAKQNPKCVGDKLEYRV*

>LJ1G059300

MESHDETGCQAPERPILCVNNCGFFGRAATMNMCSKCYKDMLLKQEQDKLAATSVENIVNSCSNGNGKQAITADAVNVRVEPVEVKAVTAQISADSSSGESLEVKAKTGPSRCGTCRKRVGLTGFSCKCGNVFCAMHRYSDKHDCPFDYRAVGQEAIAKANPVIKADKLDKI*

>LJ3G006490

MAHKIKKEETEFKVPETKTTPCTATTAATSISEPSRFFDDKATANATVTATPARSPKRSHPPDDEEAQGTTQTTPSEAKRAVNRCSGCRRRVGLTGFRCRCGELFCANHRYSDRHDCSYDYKAAGREAIARENPVVRAAKIIKL*

>LJ3G042670

MAEEHRCQAPRLCANNCGFFGSPAMQDLCSKCYRDLQLNEQQSSSAKLVLNQTLAPPLSLSPAIPQPCSSSATTTEPVVVVVAPSAPSEKEGPSRCATCRRRVGLTGFKCRCGLMLCGSHRYPEKHECGFDFKELGREQIAKANPVVKGEKLNKI*

>LJ3G045590

MDHEKTECQAPPESPMLCINNCGFFGSAATMNMCSKCHKDMVLKQEQAKLAATSIGNIMNGSSSSIETEPTEPVVAASVDIPVISVEPKTISSQPVFGFGSGSGESGEEKPKDGPKRCSSCNKRVGLTGFNCRCGHLFCAVHRYSDKHDCPFDYRTAARDAIAKANPVVKAEKLDKI*

>ME01664G00170

MAEEHRCQAPEGHRLCVNNCGVFGSPATMNLCSKCYSDYCLKERQQHQQQQEAPSKASLSVTSSLHPATAVDTQPPPAITLSEVRSRATEVTTAVEQPNRCSTCRKRVGLTGFKCRCGTTFCGTHRYPEKHGCSFDFKKVGREEIARANPLVVAEKLEKI*

>ME03203G00020

MEPQHMEPPLCANGCGFYGSVQNANLCSKIPKGTRAAIEAPTSSIARPSLDGETVAGSCDQTASKSRSNPCNSCNKRLGLMGFNCRCGNAFCRWHRQPEDHACTVDFKELGRELLIKQNPLCKADKLQNRI*

>ME03203G00060

MEPPPLCANGCGLYGSVQNANLCSKCYKEFQKQQEQAIEASTSSIARPSLNDETIAGSTDQTASNRRTNRCNSCNKRLGLMGFNCRCGNAFCRSHRHPEDHACTVDFKGLGHELLIKQNPLCKADKLEDRI*

>ME04681G00280

MDHDETGCQAPPERPILCVNNCGFFGSAATMNLCSKCHKDMLLKKEQAKLAATPTGNIVNGSASNNVEQPVVVVEAVDVHVNTVQPNTISVQPSCASGLGESVEAKPKEGPSRCGTCKKRVGLTGFKCRCGNFFCASHRYSDKHDCPFDYHSAARQAIAKANPIVKAEKLDKI*

>ME05859G00620

MAEEHRCQAQRLCVNNCGFFGSPATQNLCSKCYRDLQLKEQQSSNAKLVLNQTLVSASSSSSSSSSSSSSSSSSSSSTASVSISNPSAVEISSNQPVSATVAVNDKEETPVVRPNRCLTCRKRVGLTGFKCRCGLMFCGSHRYPEQHGCTFDFKAMGKEQIAKANPMVKAEKLQKI*

>ME06407G00310

MAQKTEKEETEFKVPETLTLCVNNCGFTGNPATNNMCQKCFNATTSTSNSTTTAAAAPSATTTTTTTAASTALGVSSGSEISSKSPRSSISRSPVRDLLPETSQKVETSIDREKIDVALAKREVNRCSCSGCRRKVGLTGFRCRCGDMFCWEHRYSDRHDCNYDYKAAGREAIARENPVVKAAKIVRV*

>ME06550G00650

MAEEHRWQAPEGHRLCANNCGFFGSPATMNLCSKCYSDYCLKERQQRQASIKASLYVSSSLSSVPAGDSQPPPAIALPEVKSHTTEVPAAVEHPSRCFMCRKRVGLTGFKCRCGTMFCGTHRYPEKHGCTFDFKKVGREEIARANPLVVAEKLEKI*

>ME06701G00810

MASRKSIDPQHCANGCGFYGSTEYRNLCSSCYREEIKKASMEIEEPLNQKSSPPPSNATSRFFNSAPRSAFSFHSNNISGSNSFFGASSSNTKQFSFSAASSLTDKTCISHRANPNNFLFGSSTVRKDVCNTCNKRVGLTGFRCRCGNKFCGKHRYPEEHSCSFDYKAFARENMLKQNQVCKGDTDKLRNRI*

>ME07035G00700

MGSEQNEGTSFPPSEPILCANGCGFFGTAANMNLCSKCYRDVRVKEDQAASAKAAMEKTLSMKSKKVDLIADTRDVAAAPNLSDHVAVNVSSSSLSEPPVAGGDQAHPKAANRCFSCNKKVGLTAFKCKCGSTFCGSHRYPENHDCSFDFKGAGRNAIAKANPVVKADKVERI*

>ME07088G00270

MGSEQNEGTSFPPSEPILCANGCGFFGTAANMNLCSKCYRDLRVKEEQAASAKAAMEKTLSIKPKQPAIIADTHDVVVVDVPPAAPNPSELVASSESSSSSSEHPVSGSDQAQLKATNRCFCCNKKVGLTGFKCKCGGTFCGSHRYSENHDCSFDFKGAGRNAIAKANPVVKADKVERI*

>ME08359G01270

MDSEQNEGTSFPPSEPILCANRCGFFGTAANMNLCSKCYRDLRIKEEQAVSAKAVMEKTLSIKSKKVDLIVDTHDVAIATAPNLSDPVALLSLGRV

>ME10963G00550

MDHDETGCQAPPDRPILCINNCGFFGSAATMNMCSKCHKDMQLKQEQAKLAASSAGNIVNGSSSNMEQTSVLDTVDIQVKTVESKTISVQPSCASALGESVETKPKEGPSRCSSCKKRVGLTGFKCRCGNLFCASHRYSDKHDCPFDYRNAAREAIAKANPIVKAEKLDKI*

>ME11106G00390

MAQKTEKEETEFKVPETLTLCVNNCGVTGNPATNNMCQKCFNATTAASTSNSTAAAPNTSTIAPASGVSSCSEILTKSPRSSKSPSPVRNPLPESSRKVETSSDRGKSDESLAGSGVNRCSGCRRKVGLTGFRCRCGELFCWEHRYSDRHDCSYDYKAAGREAIARENPVVKAAKIVRV*

>ME11106G00400

MEHNETGCQVPLDAPKLCANGCGFFGTAATMNLCSKCHKDFVLKQEQAKFASSSLKFDANGNSSSSGNEPFVGGDLPDILTESLVSSSLASSASTLNMGGDTSVKAGPTRCSTCKKRVGLTGFSCRCGSIFCSVHRYSDKHGCTYNYQTAGRDAIAKANPVVKAEKLDKI*

>ME12711G00020

MESQHMEPSLCANGCGFYGSVQNANLCSKCHKQYQKQQHQAIYKSPSLMARLPLDAETVAGSTDQTASNRRTNLCSSCNKGLGLMGFSCRCGNVFCRSHRHPEDHACSVDFKGLGRQLLIKQNPLCKADKLQDRI*

>MT00G14310

MLAGDHKIMDSHDETGCQTPELPILCVNNCGFFGRAATMNMCSKCYKDTQLMQEQEKLAAASVENLVSGGSMKQVVTDGAVNVQIENVEVKTVSAEISGDSSSSENLETKVKTGPSRCATCRKRVGLTGFTCKCGNLFCAMHRYSDKHDCPFDYQSVGRDAIAKSNPVIKADKLDKF*

>MT1G060380

MAEEQPCQAPEGHRLCTNNCGFLGNPATMNLCSKCYGDASTKSTIENTLSSSSSVTASPASPSHSTSEPIVQFINPMVTSSVVITNSVSFLVQSNRCFTCRKRVGLTGFKCRCGSTFCGSHRYPERHGCGFDFKMVGRKEIAQANPLIKAEKLRRI*

>MT1G100773

MVPTLCLNGCGFYGSPSKNNLCSKCYNDYLKENIKKSNEESFVHESSSTCPSKNPINDFCDVVEAISLIDNENMKKKKNRCKSCNKKVGPLGFECRCGDVFCGMHRYPEEHDCTVNLKEIGRQILDKQNPLCMGDKLEHRI*

>MT2G054650

MDPPPLCLNNCSSAIDKFLCPKCSDDCLTLSEIEGLILGSSPSQNSSILEIDSITVTDTTGKKNNNRCKTCNKRIGLTGFECRCGDVFCGRHRYPETHSCNVDLKSIGRQILAKQNPKCVLNKLEFRV*

>MT2G086190

MAEEHRLLPPQLCVNNCGFFGSNTTENLCSRCYRDLQLKEQQASSTKFVLNQSIAASSPAVVVFEPSSSLPDPVGPANVVVVEKPLQHNRCMTCKKRVGLTGFKCKCGRMLCGIHRYPEQHACEFDFKGLGKEQIEKANPVVKGEKLEKI*

>MT2G098160

MDHDQTGCEAAPEGPMLCINNCGFFGSAATMNMCSKCHKDMMLKQEQATLAASSIGNIMNGSSSSSGIEPAITANVEISVDPVEPKIISAEPLVASGSEESLEKKPKDGPKRCSNCNKRVGLTGFNCRCGNLYCAVHRYSDKHDCPFDYRTAGRDAIAKANPVVKAEKLDKI*

>MT3G028010

MAQRTENEETEFKVVSETLQQTTTIINLCIKNCGVVGNPSTNNMCQNCFTASTTILPPSSSRSVRSPKRSRQESSSSSEEEGSTDHDLVDEKTVSEVKRVVSRCSGCRRKVGLAGFRCRCGELFCADHRYSDRHDCGYDYKKVGREEIARENPVIRAAKIVKV*

>MT4G053440

MAEEHRCQAAQRLCANNCGFFGSPAMQDLCSKCYRDLQMKEQRSSSAKLVLNQTLIPQQSNSSSLDTGIIHPSSTSPSVMIVSSSTPTVELVAAAAGPSEAEPPKVQPNRCGTCRRRVGLTGFKCRCGLTLCGTHRYPEQHGCGFDFKGMGREEIKKANPVVKGEKLNKI*

>MT4G065570

MNMCSKCHKDMMLKQEQAQLAASSLGNIMNGSTSNTEKEPVVTATSVDIPAISVEPKTASVDIPESDDPKPKDGPKRCSNCNKRVGLTGFNCRCGNLFCAVHRYSDKHDCPFDYRTSARDAIAKANPVVKAEKLDKI*

>MT7G092400

MKFKYKQNKRWKQSVQREKEEPFQYNRIKTLLSLCTFVLFIILLLFDFLDFIVIIIIYTVLSLRFERTVLQCQDRLTLPPRWSYCRRVMESHDEMGCQAPERPILCVNNCGFFGREATMNMCSKCYKDTLLKQEQEKLVATSVENIVNGNSSSNGKLAVTASAVDVRVESVELNTVSPEVPENPISNESVEMKAKTGPSRCATCRKRVGLTGFSCKCGNLFCSMHRYSDKHDCPFDYRTAGQKAIAESNPVIKADKLDKI*

>MT7G104320

MAEEHRCETPEGHRLCANNCGFFGSSATMNLCSKCYRDIHLKEQEQAKTKSTIETALSSASASTAVVVAASPVAEIESLPQPQPPALTVPSIVPEASDNSSGPVQSNRCGTCRKRTGLTGFKCRCGITFCGSHRYPEKHECGFDFKAVGREEIAKANPVIKADKLRRI*

>MT7G114920

MVPSLCANGCGYYGLPSNKNLCSKCYNVYLKENIVLESSSSCPSKNPSINDICDAVAAISLIDSDNMKEKKTRCKSCNKKVGLTGFKCRCGDVFCGMHRYPEEHTCKVDLKKIGRQILEKQNPLCMGDKLKYRI*

>MT8G036980

MAHKITKKEETEFQVPETITPPCINPTQTTTTPTTTTTTTLTEPSRFFEDKSSKARSATSSFSQKRSHPSNDDSNVQPQTTSSEAKRAVNRCSGCRKRVGLTGFRCRCGDLFCSEHRYSDRHDCSYDYKAAGRESIARENPVVKAAKIVKL*

>PP00023G00300

MAQYGRKQDTEETNCQPPEGPVRCTNNCGFFGSAVTMGMCSKCYRDFVLTQAKTSSAKIAETTPIVSVPKEPVADRALFQSSHFLSAQPEAAGASSGASASGQDPSRPKANRCFSCKKRVGLTGFECRCGNLFCSAHRYSDKHSCTFDYKTAGRDAISKANPVVKADKMNKI*

>PP00023G00310

MSQGKDDTECQPPEGPLMCTNSCGFFGSATTLGMCSKCYRDYDSTEAKESSATGAEVVATSSAPRLLVEHSLERTKSDGSYLAAHLPGDQGTSTEVGPSASGQHPCRPQAYRCFLCKKRVGLTGFKCRCGNIFCSLHRCSDKHSCSFDYKTAGRDAIAKANPVVKADKFDKI*

>PP00050G00520

MAQDNWKQEKEETNCQAPDAPIMCTNNCGFFGSAVTLGMCSKCYRDFVLTQAKSSSGKGGEKGDTSCAAQKVAVGQRGVERTQGESVYLGGHVGGGQTEGGGTSSGGGASASGGDACRPQAHRCFSCKKRVGLTGFKCRCGNTFCSLHRYSDKHSCTFDYKTAGRDAIAKANPVVKADKVDKI*

>PP00130G00730

MATERVSQETTSQAPEGPVMCKNLCGFFGSQATMGLCSKCYRETVMQAKMTALAEQATQAAQATSATAAAVQPPAPVHETKLTCEVERTMIVPHQSSSYQQDLVTPAAAAPQAVKSSIAAPSRPEPNRCGSCRKRVGLTGFKCRCGNLYCALHRYSDKHTCTYDYKAAGQEAIAKANPLVVAEKVVKF*

>PP00138G00320

MATERVTQETTSQTPEGPVMCKNVCGFFGSQATMGLCSKCYRETVMQAKMTAVAEQATQAAQVLPSAASSAQPPVLMEEDKSSFEADSMLIQPPQSSSHHPVEVAPVTVAPQVVVAPVATPSRPAPNRCGSCRKRVGLTGFQCRCGHLFCALHRYSDKHSCTYDYKAAGQEAIAKANPLVVAEKVVKF*

>PP00194G00400

MRADHMLTGAVQARRGWGLTRFKCRGNNEFCSSHRYSDRCSCSFDFTTAGPVALAKLNPVVKLDKVEKI*

>PP00252G00080

MAQYGRKPDTEETNCQVPEGPVRCTNNCGFFGSAVTMGMCSKCYRDFVLSQSRTSSAKIAGASPPVYETQEPVADRAQIQNSCLSSTQPEAAGASSGASASGQDPSRSRPNRCFSCKKRLGLTGFECRCGNLFCSAHRYSDKHSCTFDYKLAGRDSISKANPVVKADKINKI*

>PPE_002G07330

MEHNETGCQAPPEAPKLCANNCGFFGSAATMNLCSKCHKDLILKQEQAKVAAASIDCAVNGNPNDRGKEPIATVDVDVQAGSADLMLISTQASSTPSLDIKSEEKVKETPNRCGTCKKRVGLTGFNCRCGNLFCSLHRYSDKHNCPYDYRSAAQDAIAKANPVVKAEKLDKI*

>PPE_002G07340

MKKMAQRAEKEETEFKVPETLTHCVNNCGVTGNPSTNNMCQKCFNASSATTTATSSSAVTLKFSGEKSPRSSSFSFEAPAEITRRTTASEIARSDESANRRVVNRCSGCRRKVGLTGFRCRCGELFCSEHRYSDRHVCSYDYKAAGREAIARENPVVKAAKIVRV*

>PPE_002G14300

MEPPMCAKGCGFYGCVENKNMCSKCYKDHVKQENLNAQSTTVASSVEKTDLGSIARGISLLSFQDSDDSVSNTNTSDIDNNSGSKKNRCESCNRKVGVLGFECRCGGVFCGKHRYPETHSCNLDLKKAGRDVLAKQNPLCKGDKLQCRI*

>PPE_006G04980

MEHEETGCQPHPEGPILCVNNCGFFGSVATRNMCSKCHKDMMLKEEQAKLAASSFGNIVNGTSNSNGNEPVVAAGVDVQAHLVEPKTLSLQPSFSFGSGSGGSGEAKPEGPKRCGTCNKRVGLTGFNCRCGHLFCAVHRYSDKHDCPYDYHTAARDVIAKANPVVKADKLEKI*

>PPE_006G12580

MAEEHRCQAPQLCVNNCGFFGSPTTQNLCSKCYRDLQLKEQQTLALNQTLISSASASSSSHSFLSPLISASPSHDARVERVVETKEEEEKEQHAPPARQANKCMTCRRRVGLTGFKCRCGMTFCGTHRYPEQHACGFDFRAMGKEQIAKANPVVKGEKLQRI*

>PPE_006G17370

MEQESQKRKLDETGNEASKAPIMCVNNCGFFGSEKTNNLCSKCYKDFLLKQSKTAADDTAVVGKKVSAGDQDLENKRHVQQVLEEVKQSQVEEGTTSENPEKRPANRCNFCRKRVGLTGFKCRCGQTFCSLHRYSNKHNCVFDYKSAGQEAIAKANPVVKAEKVDKIR*

>PPE_007G08220

MESHDETGCQTPDRPILCINNCGFFGRAATMNMCSKCYKDTLLKQEQANFAASSIDSIVNGSSSSIGIGPVVAGVVDVQAGQVETRVVSTEPSIDSSSSMVFEVKEKEGPSRCTTCRKRVGLTGFNCKCGNTFCASHRYSDKHDCPFDYRTAGQDAIAKANPVVKAEKLDKI*

>PPE_007G13570

MAEEHRCQAPEGLRLCANNCGFFGSPATMNLCSKCYRDFCLKEQQEASIKSTVEASLSASSSSSVSAPSSSSTSSSFSPVIETLCQPPPPALTLPEVAGDVVGHSAGDVRPPEAVAVVSQPNRCTVCRKRVGLTGFKCRCGTTFCGVHRYPEKHACSFDFKTLGREEIARSNPLVIAEKLEKI*

>PT01G01860

MDHDETGCQAPPEGPILCTNNCGFFGSAATMNMCSKCHKGMLLKQEQANLAASSIGSIVNGSSSSNVFEPVIADIIDVQNNAVEPKTITVQPSCASGSGERVEAKPKEGPNRCTSCKKRVGLTGFKCRCGSLFCASHRYSDKHDCPFDYRSAAREAIAKANPVVKAEKLDKI*

>PT01G11500

MGSEQNEGTSFPSSQPQLCANGCGFFGTAANMNLCSKCYRDLRAEEEQAAFAKAAMEKTLNMKSQQHIDSRVAVDAPQVAVANSMQSAEASSSAETAVAAGDQVSSKPANRCFSCNKKVGLTGFMCKCGGTYCGTHRYSENHECSFDFKGAGRNAIAKANPVIKADKVGRF*

>PT01G26930

KGDSDAGKWSRTRPEPQGPSKAPKLCANSCGFFGTATTMNLCSKCHDDFILKQEHAKMALSICCVCYERFLSVDTTSNAVIAVAVDPPATSAVPLISSIPALSAAASSTDHTSIQYLSLRESFLRAIHHYPDKHNCSSDYRSAGQDAIAKANPIVKAEKLDKI*

>PT01G26940

MAQRTEKEETECKVPENLTLCINNCGVTGNPATNNMCQKCFNASTSTSNPSSSTTTTTTTITFAATTNGVSTNEILKFTSEKSLRSSISRSPAKDHQRQPKTASDKERSDSSSVAKKEVNRCSGCRRRVGLTGFRCRCGELFCWEHRYSDRHDCSYDYKTVGREAIARENPVVKAAKIVRV*

>PT03G11710

MGSEQNDGTSFPPAEPKLCVNGCGFFGTAANMNLCSKCYRDLRAEEEQAASAKAAMEKTLNINPKQNIDSKVVVDAPQVVVANSVQSVVSAEASSSAETVVAGGDQVPSKPANRCFSCSKKVGLTGFQCKCGGTYCGTHRYAENHECLFDFKGAGRDAIAKANPVIKANKVERF*

>PT03G20550

MDRDETGCQAPPERPILCINNCGFFGSAATMNMCSKCHKDMLLKQEQTKLAASSIGSIVNGSASSNVNEPVIADTINVQINAVEPKTITVQPSCASVSGERVEAKPKEGPSRCTSCKKRVGLTGFKCRCGDLFCASHRYSDKHDCPFDYRTAAREAIAKANPVVKAEKLDKI*

>PT04G18430

MAEEQHRCQEQRLCVNNCGFYGSQATENLCSKCYRDLHQSQPLNHQLLNPSSSSAASVSSFASPAVDVLKVNTNQKAPVVVVGDDKKDEVKAGEPAAVKQQQQPSRCLTCRRRVGLTGFKCRCGMVFCGTHRYPEQHDCEFDFKSLGKQQIAKANPVVKGEKLQKI*

>PT06G05650

MESHDETGCQAPEGPILCINNCGFFGSAATMNMCSKCHKDIILKQQQAQLAASSIESIVNGNSSGNGKEPVVAVAVDVQSAPVEVKIISTEPSSATSSKPSEMKAKEGPSRCTSCRKRVGLTGFSCRCGNLFCAVHRYSDKHNCRFDYRNAARDAIAKANPVVRAEKLDKI*

>PT07G07850

MEQESQKRKLEDSSSNEAQNTPILCVNNCGFFGSPNTNNLCSKCYKEFLLTQQDTTTTTIDVPVHVAENSAAAAAEVEGQQEGGEEEKRPVVVANRCNFCRKKVGLTGFKCRCGYTFCSQHRYSDKHNCVFDYKSAGQDAIAKANPVVKADKIDKI*

>PT09G06390

MAQRAEKEETEFKVPETLTSCINNCGVTGNPATNNMCQKCFNASTSTSNSSSSSTATSMTFAATATSVSNNEILKFTGEKSARSSISRSLVKDPQKSPETASDKERSCAYHVAKKEVNRCSGCRRRVGLTGFRCRCGELFCWEHRYSDRHDCSYDYKTAGREAIARENPVVKAAKIVRV*

>PT09G14410

MAEEQHRCQEPRLCVNNCGFFGSPATQNLCSKCYGDLRQSQPLNQLLAPSSSASVSSFSSPTVDVIKNQIAPVLVVEGDEKGEFKAEPTVVVPQQKPNRCLTCRRRVGLTGFNCRCGMVFCGTHRYPEQHDCEFDFKSLGKEQIAKANPVVKGEKLQRI*

>PT11G14360

MLVVYRFQSPRPVVVANRCNFCRREVGLTGFKCRCVYTFCSQHRYSDKHNCVFYYKSILDRMLFLKGNSVVKQIRLIKI*

>PT12G13000

MDSQSNLTPPLCAKDCGFFGSPEKKNLCSECYRDYVKEEESVAAETAKKLSQLVINTPSAANDKSPAVLTDETTSSSAAAAAAASSSTVKINRCECCNKKVGLLGFKCRCEKTFCGVHRHATEHSCTFDFKTLGRHILAEQNPLVVSDKLHTRI*

>PT12G13010

MDSINSSTLPLCAKGCGFFGSPENENFCSKCYKDYLKEGLIAEPSKKLSEPIVVTPSFDDNSPDVVTDETTSTTTAVASTSKVKNRCECCNKKVGLMGFECRCGNTFCGVHRYPKEHSCTFDFKTLDQQNLAKQNPLVAGDKLGSRI*

>PT15G13150

NQAVVSNETASTASTTASTSTVMKNRCECCNKKVGLMGFKCRCGKTFCGVHRYVKEHSCTFDFKTYDRQNLAKQNPLVAGDKLHTRI*

>PT15G13190

MDSQDKLTPALCAKGCGFFGSPENKNLCSKCYKDYLKEEVIAKTADKLSELVITPSSDDKNPAVVSNETASTTTATASATTVLKNRCECCGKKVGLMGFKCRCGKTFCGVHRYAKEHSCTFDFKTFDRQILAKQNPLVAGDKLDARI*

>PT16G05170

MESHDETGCQAPEGPILCINNCGFFGSAATMNMCSKCHKDIILNQQQAQLAASSIESIVNGNSSGNGKEPVVAGAVDVQAAPVEVKIISTEPSIASSKPSEMKAKEGPSRCTACRKRVGLTGFGCRCGNLFCAIHRYSDKHDCPFDYRTAARDAIAKANPVVKAEKLDKI*

>RC29625G00060

MAEEHRCEAPEGHRPCVNNCGFFGSAATMNLCSKCYRDYCLKEQHQHQQEASIKASLSVPSPSSAAAIVDNSQPAPSPDDVLRSPEAVEEEVKVVVTAIEQKQPNRCFVCRKRVGLTGFRCRCESMFCGAHRYPEKHNCTFDFKKIGREEIARANPLVVAQKLHKI*

>RC29820G00030

MEPQNSSSSASICASVSSDKKIDTTNQSVPTCIDGERGSSATKIAPCSSSSSSSSSSPTVKNRCRSCNKKVGLTGFACRCGKVFCGMHRYSDEHRCTFDYKEFDRQILVKHNPVIRGDKLDDRV*

>RC30055G00090

MAEEHRCQAQRLCVNNCGFFGSPATQNLCSKCHRDLQLKEQQSSNAKLAFSQTLSASSSSSSSSSSSPSAASVSISISSPPIVDLPEVSSKAEVVVEDKEETAAETVVRPNRCLTCRKRVGLTGFKCRCEMVFCGTHRYPEQHGCTFDFKAMGKKQIAKANPVVKAEKLEKI*

>RC30147G01240

MESRKRIDPPHCVNGCDFNRSIKNCNLCSKSFREKEDIEIANEAIASSSMESLKQELKPRFFSSGDSTNANAIFGANSLFGACSRTSKKSCFSTDYNNAASGSIFKFTTLPEISVRNFTFGTSTVTNKRCNRCNKRVGLLGFGCKCGHLFCGKHRYPEEHKCRFFYKLMRYKYFSVPSPKPRFSATAITDGVRSNCFCRA*

>RC30147G01250

MDSPNSSVTPPSCARGCGFFGSAENRNMCSKCYTDYLKQEIIAKTNSAPEPSPASNSLSNKIATCINIKEAAAEEEETAKSVPVAKNRCESCNKKVGVTGFACRCGKVLCGTHRYPKEHCCTFDFKRADRDLLVKQNPLVKAKISHVLAKIANAGSQLLYPGQTRL*

>RC30152G00160

MCQKCFNATTSTSTSNSTTSTTTTAIISASAGSSGSGILTISPRSSASLMVIRDPQPETSESTADREKNDVLVAKRDVNRCSGCRRKVGLTGFRCRCGDLFCWEHRYSDRHDCSYDYKTVGREAIARENPVVKAAKIVRV*

>RC30169G01820

MDHDETGCQAPPERPILCINNCGFFGSAATMNMCSKCHKDMLMKQEQSKLTASSAGSILGGSSSSSLEQLIAAGTVNIQPNVEESKPIAVQPSYISELGESVEAKPKEGPNRCNACKKRVGLTGFKCRCGNLFCASHRYSDKHDCQFDYRNAAREAIAKANPIVRAEKLDKI*

>ST01G020240

MEHDETGCQPPPEGPILCINNCGFFGSAANMNMCSKCYKDMVLKQEQAKLAVSSIENLVNGSSAGEKGMVIVDPVDVQPDTIEAQSIALPSSQTSSSSDMPDVKAKVGPNRCGTCKKKVGITGFKCRCGNIYCGAHRYSDKHDCLFDYRSAGQDAIAKANPVVKAEKLDKI*

>ST01G026560

MNMCSKCYKDMIFKQEQANFAASSIESFVNGSSSASVKAVDVAVTVQEGPAESLVIPTQVAAPVESVQVEKAKEGPNRCSTCRRRVGLTGFNCRCGNLFCSAHRYSDKHECPYDYHKAGQDAIAKANPVVKAEKLDKI*

>ST01G026570

MAQRTEKEETEFKAVPETITLCINCGVTGNPATNNMCQKCFNATTAATSTSSSSPTGSAVTIPHKFAEKLVRSEKSARFSSLRSLPDRKSDLDRMSQDLKKVGDWMMVKEEDQLKESLPPAKREVNRCSGCRRKVGLTGFRCRCGELFCGEHRYSDRHDCNYDYKTAGREAIARENPVVKAAKIIKV*

>ST02G011060

MTSCDKTENPTLCSTNCCSYANSRNHDSYDEIYLFHKMFSLTIRTGKDDLMMKTKPQRCMICRKKVGLIGFNCKCDEVFCKTHRYPEEHACTYNFKSIGRAILAKENPLCKADKLKNRI*

>ST03G009990

MIVEPIVLISDLVEAAAPQVVTPQSNRCLICRKKIGLVGFKCRCGTIFLGPIGIQRFMHAHLSFKSMGREAIAKANPLIKAEKFKKI*

>ST07G022130

MCRGLWFYGTSSNHNLCSQCYKAFLKEEEAKNIAVLSEKISSLTFHEGTTTENVDLTMKIKQRCMTCKKKVGLIGFSCRCKGMFCSVHKYPEEHACTFDYKSFGRVTLAKENPPCRHDKLENRI*

>ST07G022140

MTSCGKVENQILCARGCGFYGTSSNNNLCSQCYKAFLKEGEAKNVSYKKSSLIFHDDSEGTTKNVESTMKIKQRCMTCKKKVGLTGFSCQCEGMFCKVHRYPEEHACTYTISSQLVM*

>ST07G022150

MTSCGKVENPILCAGGCGFYGTSSNHNLCSQCYKTFLKEEAKNIVVLSEKISSLTFHEGTTTENVDSTMKIKQRCMTCKKKVGLIGFSCRCEGMFCSVHKYPEEHSCTFDYKSFGRVTLAKENPPCRHDKLENRI*

>ST07G022160

MTSCGKVENQILCARGCGFYGTSSNNNLCSQCYKAFLKEGEAKNVSYKKSSLIFHDDSEGTTKNVESTMKIKLRCMTCKKKVGLTGFSCRCEGMFCKVHRYPEEHACTYDFKSVGHVTLAKENPPCRSAKLENRI*

>ST08G025520

MGSEGNKFNDGTSFSPADSILCSNGCGFFGAAATNGLCSKCHRDFKMKEDHAAMAKVAMDKLVISRPQVESIGKVDFCSSATVTAAEKPVVETAVAVEIAGSQPNRCLSCRKKVGVVGFKCRCGSTFCGTHRYPEKHDCTFDFKAKGKEEICKANPVVKADKIQRF*

>ST09G010540

MAEEHEFQSPEGGRHQLCANNCGFFGNSTTENYCSKCYRDIEERKSDAKSIDCLFSPTKRVLEKMIVKPIVLTPDLVEAAVPLVTPQPNRCLVCKKKMGLMGFKCRCGTIFCGTHRYPEVHACTFDFKSMGREAIAKANPLIKAEKLKKI*

>ST10G020750

MESSKETGCQAPEGPILCINNCGFFGSAATMNMCSKCHKDMILKQEQAKFAATSIENIVNGNSSSNGKEPIATGAINVQPGSADLKVISTEASSDLSSGPSSEVKPKEGPTRCTTCRKRVGLTGFNCKCGNLFCAAHRYSDKHECPFDYKNAGRDAIAKANPVVVAEKLNKI*

>ST10G025720

MAEEHGFEAPEGHILCANNCGFFGSPTTQNFCSKCYNEVYIKGGQQKPIDSLFPPQLPIPSQSSSVLVLPEPAAVEEKPEVVIAAVTAAVQPMPAQSNRCSACRKKVGLIGFKCRCGTTFCGTHRYPEIHGCSFDFKSMGREAIAKANPVVKAEKLGKI*

>ST11G011630

MAEEQRMQEGGGHRLCANNCGFFGSPTTLNLCSKCYKDHCMKEQQSRTAQLAMEKTRPQQQQSESTSTYIPCTKSLPILEVSQPRETEIATRAPQVQSDTAAEVPQVQLNTVADQTPQVQSNRCATCRKRIGLTGFKCRCGVTFCGSHRYPEHHGCTFDYKSMGKVAIAMANPLVKAEKLHKI*

>ST11G016470

MEHDETGCQPHPEGPILCINNCGFFGSAANMNMCSKCYKDVILKQEQAKLAASSIENFVNGSTSQKGPVIVGSVDVQPALLESKSVIFSSPPSSSSGEAAELMAKEGPSRCSTCKKKVGLTGFKCRCGNFYCGSHRYSDKHDCQFDYRSAARNAIAKANPVVKAEKLDKI*

>ST12G032310

MAAQKREKEETELKVPESIPLCSPTLPVPSPSPPPTTHLSVALISDLKRSDRSSTERVDLKVSSMDDQSRSTSAASPESTDLVGRKTGVKRQREANRCSGIGCRRKVGLMPFRCRCGEVFCSEHRYSDRHDCSYDYKAAGREAIAKENPVVKAAKILKV*

>TC0001G08430

MANIDLPPLCAKGCGFYGSSETKNLCSKCYNDFLKELVSKSKSEPKVDTALTASCPSVPVDSSLASAPSKLKNRCESCNKKVGLMGFSCRCGKVLCDVHRYPQEHLCNFDFKKADRLILVKENPIIKADKLDSRI*

>TC0001G40740

MEQNQRGCQAPKLCANNCGFFGTAATMNLCSKCHKDLVMKQQGDKLASSPNGSILDGSPGNNHEPVSVAVDPQSTSVKSTAITAQANSASISMNTGREEEVKRGPNKCSICGKRVGLTGFTCRCGDLFCAVHRYSDKHDCPYDYHCAARDAIAKANPVLKANKLDKI*

>TC0001G40760

MAQRTEKEETEFKVPETLTLCINNCGVTGNPATNNMCQKCFNATTATPSSSSSSSSSATSPSATGGAIAGGASIPKFSDDQSSRSTPSRSQQNRSDSSPPTTAATVTNSRTTAWNRSGNDPAAAAEKKVVNRCSGCRKRVGLTGFRCRCGELFCAEHRYSDRHDCSYDYKTAGREAIARENPVVKAAKIIRV*

>TC0003G16430

MESVESMDRKLCAKACSFNGSAQRNNLCSQCYKDFLVGEFQNHNPIGEPLIPTTDQPLNSCFTVPPYSVSHVNNSNGSVGFTFGWTNNSGGASLASTKNRCNSRNKRVGLTGFTCRCGNLFCGKHRYPEEHECCVDLKAIGREALVKENPDCKGVGPDNLIEVYGALTIAKNQMENSVFSNATFLHDQNLLGDYGFSSGIMFCQIRSDMAGESHSLKGAPELPSKGVVALPSKGAVVISFGGDLVLLDVEECQI*

>TC0003G26090

MGSEQNEGTSFPPSEPKLCANGCGFFGTAANMNLCSKCYRDLRAGEEQAAKAKAAMEKSLSVKTKQEDVVVETILDVKPVEELPHVGSSSTAVEQPAVVAAGNEQAEPKVSNRCFICRKKVRLTGFKCRCGSTFCGEHRYPEKHECLFDFKGAGRDAIAKANPVVKADKVERF*

>TC0005G08290

MAEEHRCQAPEGHRLCVNNCGFFGSPATMNLCSKCYRDFRLKEQQEASSIKSSLSSSPTSSSTVVESVSQVPLLTLPEVNGESPVPAVEIAPATAEQRPQQQPNRCMVCRKRVGLTGFRCKCGITFCGSHRYPENHGCSFDFKTIGREEIARANPVVKAEKLEKI*

>TC0005G16620

MILMSWRLEKMESHDETGCQAPEGPILCVNNCGFFGSAATMNMCSKCHKAMILKQEQVQLAASSIGSIVNGSSSGNGKEPTLAAALDVQSGIFESKNGSAEPSIDPSRMTFGGMKIKEGPNRCTTCRKRVGLTGFNCRCGNLFCAAHRYSDKHDCPFDYRTAARDAIAKANPVVRAEKLDKI*

>TC0005G22760

MANANLPPLCAKGCGFYSSSQTKNLCSKCYNDFLKELIAKSTAEVKVDPSSAAPNPSVSVDSSSVPTPSKLKNRCESCNKKVGLMGFSCRCGKVLCGVHRYPKEHSCNFDFKTADRLILAEENSLVMADKLESRI*

>TC0009G22510

MDEIIFACMSRRRGEEMDHEKTGCQAPPERPILCINNCGFFGSAATMNMCSKCHKDMILKQEQAKLAASSIDNIVNGSSTGNGNETVIATGVDVPNSVEPKTILVQTSCPSGSGESIEAKLKEGPIRCSTCKKRVGLTGFKCRCGNLFCTSHRYSDKHDCPFDYRTAARDAIAKANPVVKAEKLDKI*

>TC0009G28100

MAEEYRSQAPQLCANNCGFFGIPATQNLCSKCYRDLQLKKQQSSSAKQAINQTLVPLSSPSSSLPSSSSISSSPFSGSLPMKEVETDEEVKVEEIQIQVRPNRCLACKKRVGLTGFTCRCGMVFCGTHRHPEQHDCTFDFKEMGKEQIAKANPVVKGEKLQKI*

>TP1G11040

MGSEQNDSTSLSPSEPKLCVNGCGFFGTPSNMNLCSKCYRDIRATEEQAASAKAAVDKSLNPNKPHIKPQQSREIAPGVESGSSSSTSGGDSSAASSDPPKPTRTTRCLSCNKKVGVTGFKCRCGSTFCGAHRYPESHDCEFDFKGAAREAIAKANPLVKADKVERI*

>TP1G37930

MDHDKTGCQSPPEGPKLCINNCGFFGSAATMNMCSKCHKAILFQQEQGAKFASAVSGTSSSSTIMKETFTSALVDVETKSVEPMVVAVQPSSVQVVAEVEAQEAAAKPKEGPSRCTTCNKRVGLTGFKCRCGDLFCGTHRYADIHNCSFNYHAAAQEAIAKANPVVKAEKLDKI*

>TP2G01660

MGSEQNHSTSFSPSEQKLCVNGCGFFGTTANMNLCSKCYHDLRITEEQAASAKAALEKSLNPKPKTSLESAGASSSSTGSETAAFSESSPPPQTRAKKRCLSCNKKVGLMGFKCKCGSTFCGDHRYPENHECEFDFRGEGRDAIGKANPLVKAEKVDRV*

>TP3G10770

MAQRTEKEETEFKVLETLTTPATTPTLCSNNCGVTANPATNNMCQKCFNASVAAAGVDSGSILKRSARSVNLRSTPAKVVIRTREIDPVKRDQQTVNRCSGCRKKVGLTGFRCRCGDLFCAEHRYSDRHDCSYDYKTAGREAIARENPVVKAAKMVKV*

>TP4G10400

MAEEHRLQEPRLCANNCGFFGSTATQNFCSKCFRDLQHQQQNSSTAKHALNQTLASVSTGGVASSSVSSPTPPPPSPQADLTSSDPKEVNVTNAEKRVAAEEEEEKAPSQDPKRCLTCRRRVGITGFRCRCGFVFCGTHRYAEQHECSFDFKRVGKEKIAKANPIVKAEKLEKI*

>TP4G18560

MAEEHRCKTPEGHRLCLNNCGFLGSSATMNLCSNCYGDLCLKQQQQASMKSSLSVASPLSSSEIDSIPSSSSSSSSTIAPVLENYAREKQIPTTATEQKEPQPPQRPNRCSLCRKRVGLTGFMCRCGTSFCGRHRYPEVHGCSYDFKSAGREEIAKANPLVIAAKLQKI*

>TP5G09530

MAEEHRCQTPEGHRLCANNCGFLGSSATMNLCSNCYGDLCLKQQQQPSMKSTVESSLSTVSPPSPEIASISSPIIQPLVQNPSAELEVTVKNASKTVTTTEEQQQQKRPNRCTTCRKRVGLTGFKCRCGTTFCGAHRYPEVHGCTFDFKSAGREEIAKANPLVKAAKLQKI*

>TP6G05470

MSSEQNNSTSFPPTEPKLCDNGCGFFGSPSNMNLCSKCYRSLRAEEDQTAVAKAAVEKSLKLPSCSLITTQEPKQPLETKPASVESVVVVSAETSSVPVATEQDEAEPSKPARPNRCFSCNKKVGVMGFKCKCGSTFCGSHRYPEKHECSFDFKEVGRDEIAKANPVIKADKVQRI*

>TP7G12090

MTGEPSLCIKGCGFFSTSQTKNLCSKCYSGFKDESDRHLAALKDHTETVAEAAEEVTVAAEAEEAVVVRKESSRCNACKKKVGILGFRCRCGNVFCGSHRYPEEHSCPSDYKSAAINDLIIQNPIVNGDKLNRI*

>TP7G20870

MGSEQNDSTSFTQSSDPKLCANGCGFFGSPSNMDLCSKCYRNICAEEAQTAVAKAAVEKSFKPSPPRTLFISEPAVKPSEPEKEKAVVAAVVVAEPSSIVGEGEEAAVPDQNEPSKPARPNRCLCCNKKVGIMGFKCKCGSTFCGEHRYPERHDCSFDFKEAGRGEIAKANPVIKADKLQSMRKSEIRIYLQTQLTQVDS*

>TP7G23400

MVNASETEALPCAGGCGLFGTRKNNNLCSLCYKQSLLERVATLRLEPETERSTVCPPTSSPVAAEEPVKKRRCGTCQRKVGMLGFNCKCGHVFCGSHRYPEEHYCPFDYKQSGRLALARQLPLIRTEKLHKF*

>VV02G07850

MSFNPKLLQENRTSSFSFLDTAGGVGGGDKSEPKVPNRCMSCNKKVGLTGFRCKCGSTFCGAHRYPEKHECTFDFKASGRDAIAKANPVVKADKLDRL*

>VV02G07880

MQSPYGHEIQLSLETKSAFCGLSWSCVGGGDKSEPKVPNRCMSCNKKVGLTGFRCKCGSTFCGAHRYPEKHECTFDFKASGRDAIAKANPVVKADKLDRL*

>VV06G06330

MDHDETGCQAHPEGPILCINNCGFFGSPATMNMCSKCHKDMMLKQEQAKLASSFSFGSEGSGEAKPKEGPNRCSTCKKRVGLTGFNCRCGHLFCATHRYSDKHDCPFDYRTAARDAIAKANPVVKAEKLDKI*

>VV06G06360

MCRDLKVPQISTLKSRMAQKRENEQATETELKVPETLTLCVQTCGFSASDKPRSRSPSPPDDPDSTLENSDQGAVRRREVNRCSGCKRKLGLIGFRCRCGEMFCSKHRYSDRHECRFDYKAAGREMIAKENPVVRPAKILKV*

>VV08G07910

MESHDETGCQAPEGPILCINNCGFFGSAATMNMCSKCHKDLALKQEQAKLAASSIGSIVNGSSSGNGKEPIEGPNRCTACRKRVGLTGFNCKCGNLFCAVHRYSDKHDCPFDYRTAARDAIAKANPVVKAEKLDKI*

>VV13G07540

MVKGNSHTRLYEAHIPSPLPTFESLKIPFSEPQIFILQTLDMAQKTEKEETEFKVPETLALCVNNCGFTGNPATNNMCQKCFSASASAAAAAAAGALKTNGLAGGDQPGSDEGRCLSCGASVHGSARVQLRLQDRRSRGYRAGKSGSESCEDRESLNIIGQKKKNQTKIVKFRGRGSRSRSLHLSEIVLSRSTLRESALERFVESFADIKMKKVSLVCSCWINLLFSFFLLFGFFVIHDPV*

>VV13G07550

MDHNETGCQAPPEAPILCINNCGFFGSAATMNMCSKCHKDLVLKQEQAKLAASSFESIVEGSSNCNAKESMGPNRCSSCRKRVGLTGFNCRCGNIFCAVHRYSDKHACPFDYRTAARDAIAKSNPVIKPEKLDKI*

>VV16G03240

MDPPLCVNGCGFFGTPATQNLCSKCYKDFLKEEEEAAKAKTKSMEKAMGSTVASTSSVDDVVTSMTQLSLSSENTKKTISDDSSTKKKVERCETCKKKVGIIGFKCRCGSMFCAEHRLPEKHECSFDYKTMGREILKKQIPLIKPDKLEGRV*

>VV16G03440

MSQRVNKKKHFKRILAMTFKSNILGINLKRVGNMEDPILCANGCGFFGTTATRNLCSKCYRDFLKEEEESTKTKVMSMKKAMGPRVESTSSLDDEKEKNSESSANKRKPATRNLCSKCYGDYLKEEGESAKAKAMSMEKAMGPRVESTSSLDYVVTSMAQLSLSSENTNKVINGDLL*

>ZM01G32310

MAQRDKKVEEPTELRAPELTLCANSCGFPGNPATNNLCQACFLAATASSSASASVSPPPPSSSSSSPAVLQFDEQQQQQNPRPRAPAASGPTEEPPRPARASAPAPAPASSSSVRRCQTCRKRVGLTGFRCRCGDLFCGAHRYSDRHDCCFDYRAAGRDAIARDNPVVRAAKIVRF*

>ZM01G55640

MTQKRKSIGRGGVEDCGSPARAAACTSTTAASTSTTVEEKNTPAVFETTPPLWMTGPAETKKPKIASSSSSSSSSSSSSPDGGSNNAAVAQAQPQPPPANRCSACRKKVGLLGFRCCCGKTFCGAHRYAEKHACGFDYKHAGRGRIAKENPIIVADKIAKI*

>ZM02G32660

MAQRDKKEEPTELRAPEITLCANNCGFPGNPATQNLCQSCFSASRSPSSPTSSSSSLASAASQPRPAALVVDAAAVEALAAPAAAAVGQATEAAARASASRCSSCRKRVGLTGFRCRCGELFCGAHRYSDRHGCSYDYRGAARDAIARENPVVRAAKVVRF*

>ZM04G15730

MAEEQQHQRWQEGHRLCANNCGFFGSPATLDLCSKCYRDLYQQQPAGAAAGPSAPTASAFQHSSSAVSGAAAVSPDLEPPATAPAGAKAGRCSSCRKRVGLTGFACRCGATFCGVHRYPERHACAFDFRAAGRDAIARANPVVKGDKLKDKI*

>ZM04G38190

MEHKEAGCQQPEGPILCINNCGFFGSAATMNMCSKCHKEMIMKQEQAQLAASSIDSIVNGGDNGKGPAIAATVGVAVPQVEEKTIAVQPMHVAETSEAAAVIAKAKEGPNRCATCRKRVGLTGFNCRCGNTYCSMHRYSDKHDCQFDYRTAARDAIAKANPVVKAEKLDKI*

>ZM05G23560

MEHKEAGCQQPEGPILCINNCGFFGSAATMNMCSKCHKEMITKQEQAQLAASSIDSIVNGGDGGKGPVIAASVNVAVPQVEQKTIVVQPMLVAETSEAAAVIPKAKEGPDRCAACRKRVGLTGFSCRCGNMYCSVHRYSDKHDCQFDYRTAARDAIAKANPVVRAEKLDKI*

>ZM07G17780

MAQRDKKEEPTELRAPEIALCANNCGFPGNPATQNLCQSCFSASRSSSSSSQPSPTSSPSASAPAAAVPQPRPALLDAALQLAPPAAAAGQPVEASARTSANRCSSCRKRVGLTGFRCRCGDLFCGAHRYSDRHGCRYDYRGAARDAIARENPVVRAAKIVRF*

>ZM09G00120

MEHKEAGCQQPEGPILCINNCGFFGSAATMNMCSKCHKEMIMKQEQAQLAASSIDSIVNGGDNGKGPAIAATVGVAVPQVEEKTIAVQPMHVAETSEAAAVIAKAKEGPNRCATCRKRVGLTGFNCRCGNTYCSMHRYSDKHDCQFRLSNCS*

>ZM09G15600

MEHKEAGCQAPEGPILCINNCGFFGSAATMNMCSKCHKEMITKQDQAKLAASSIDSIVNGSDAVMEPVVAGSNTVVAVAQVELQTMNVQQPADVAGPSEGVAAISKGGKVGPNRCSACRKRVGLTGFNCRCGNLYCALHRYSDKHDCKFDYRTAARDAIAKANPVVKADKLDKI*

**A3. Sequence logo for the A20 domain in 26 MdSAP genes**

>MdSAP1

NMEPPRCAKGCGFFGSVTNMNMCSKCYRECLKE

>MdSAP2

MTERPCCANGCDFYGSVETKNLCSRCYXDYLKQ

>MdSAP3

.MEPPMCASGCGFYGTVENKNMCSKCYKDHLKH

>MdSAP4

.MEPPMCASGCGFYGTVENKNMCSKCYKDHLKH

>MdSAP5

PETLTHYVNNCGVTDNPSTNNLCQKCFNTATTS

>MdSAP6

PETLTHCVNNCGVTGNPSTNNMCQKCFNAASAA

>MdSAP7

PETLTHCVNNCGVTGNPSTNNMCQKCFNAASAA

>MdSAP8

PEAPKLCANNCGFFGSPATMNLCSKCHKDLVLK

>MdSAP9

PETLTHCVNNCGVTGNPSTNNMCQKCFNAASAA

>MdSAP10

PEGPILCVNNCGFFGSAATMNMCSKCHKDMMLK

>MdSAP11

PEGHHLCANNCGFFGSPATMNLCSKCYRDFCLK

>MdSAP12

PEGHHLCANNCGFFGSPATMNLCSKCYRDFCLK

>MdSAP13

PDRPILCVNNCGFFGRAATMNMCSKCYKDTLLK

>MdSAP14

PETLTHCVNNCGVTGNPSTNNMCQKCFNAASAA

>MdSAP15

PEAPKLCANNCGFFGSPATMNLCSKCHKDLVLK

>MdSAP16

PDRPILCVNNCGFFGRAATMNMCSKCYKDTLLK

>MdSAP17

NMEPPLCAKGCGFFGSVTNMNMCSNCYRQYLKE

>MdSAP18

CQAQQLCVNNCGFFGSPTTQNLCSKCYRDLQLK

>MdSAP19

CQAQQLCVNNCGFFGSPTTQNLCSKCYRDLQLK

>MdSAP20

CQAQQLCVNNCGFFGSPTTQNLCSKCYRDLQLK

>MdSAP22

PEGHHLCANNCGFFGSPATMNLCSKCYRDFCLK

>MdSAP23

PEGPILCVNNCGFFGSAATMNMCSKCHKDMMLK

>MdSAP24

PEGPILCVNNCGFFGSAATMNMCSKCHKDMMLK

>MdSAP26

PDRPILCVNNCGFFGRVATMNMCSXCYKDTLLK

>MdSAP29

PEGHHLCANNCGFXGSPATMNLCSKCYRDFCLK

>MdSAP30

PEGHHLCANNCGFXGSPATMNLCSKCYRDFCLK

**A4. Sequence logo for the AN1 domain in 28 MdSAP genes**

>MdSAP1

QSSQGSSDSSEKKRCLSCKKRVGPTG.FECR.CGGVFCGKHRYPEEHSCCVDYKK

>MdSAP2

CSKSSSGSTSVKNRCESCNRKVGVLG.FSCR.CGGVFCGTHRYPEKHCCHVDFKM

>MdSAP3

NMGTSSVGVIKKNRCQSCSRKVGVLG.FQCR.CGGVFCGTHRYPEEHSCDVDLKQ

>MdSAP4

NMGTSSVGVIKKNRCQSCSRKVGVLG.FQCR.CGGVFCGTHRYPEEHSCDVDLKQ

>MdSAP6

ARSDESPNRRVVNRCSGCRRKVGLTG.FRCR.CGELFCSEHRYSDRHVCSYDYKA

>MdSAP7

ARSDETPNRRVVNRCSGCRRKVGLTG.FRCR.CGELFCSEHRYSDRHVCSYDYKA

>MdSAP8

NIKREEKVKETPTRCGTCRKRVGLTG.FSCR.CGDLFCAVHRYSDKHNCPHDYRT

>MdSAP9

ARSDETPNRRVVNRCSGCRRKVGLTG.FRCR.CGELFCSEHRYSDRHVCSYDYKA

>MdSAP10

SGSSGEPRPEGPKRCNTCNKRVGLTG.FNCR.CGHQFCAVHRYSDKHDCPYDYRT

>MdSAP11

RAPEVATVVSQPNRCTVCRKRVGLTG.FKCR.CGTTFCGVHRYPEKHACSFDFKT

>MdSAP12

RAPEVATVVSQPNRCTVCRKRVGLTG.FKCR.CGTTFCGVHRYPEKHACSFDFKT

>MdSAP13

SMKIEMKENKGPSRCTTCRKRVGLTG.FNCK.CGNTFCASHRYSDKHDCPFDYRT

>MdSAP14

ARSDESPNRRVVNRCSGCRRKVGLTG.FRCR.CGELFCSEHRYSDRHVCSYDYKA

>MdSAP15

NIKSEEKVKETPTRCGTCRKRVGLTG.FSCR.CGDIFCAVHRYSDKHKCPYDYRT

>MdSAP16

SMKIEMKENKGPSRCTTCRKRVGLTG.FNCK.CGNTFCASHRYSDKHDCPFDYRT

>MdSAP17

QSSQGSSDLSQKKRCLSCKKRVGPTG.FECR.CGGVFCGKHRYPEEHSCSVDYKK

>MdSAP18

EKEAAPSAGAQANRCTTCRRRVGLTG.FKCR.CGMTFCGTHRYPEQHACGFDFRG

>MdSAP19

EKEAAPSAGAQANRCTTCRRRVGLTG.FKCR.CGMTFCGTHRYPEQHACGFDFRG

>MdSAP20

EKEAAPSAGAQANRCTTCRRRVGLTG.FKCR.CGMTFCGTHRYPEQHACGFDFRG

>MdSAP21

GNCDPKNKKKPTCPVRRCKETLTFSNTSTCKTCQIKVCLKHRFPADHVCRKQTAA

>MdSAP23

SGSSGEPKPEGPKRCNTCNKRVGLTG.FNCR.CGHLFCAVHRYSDKHDCSYDYLT

>MdSAP24

SGSSGEPKPEGPKRCNTCNKRVGLTG.FNCR.CGHLFCAVHRYSDKHDCSYDYLT

>MdSAP25

PSNYEKATKKKKCPIPGCKEILTFSNTIKCRDCMVDHCLKHRFGPDHKCPGPKKP

>MdSAP26

SMKIEVKEKKGPSKCTTCRKRVGLTG.FNCK.CGNTXCASHRYSDKHDCPFDYKT

>MdSAP27

GNCDPRKKKKPTCPVRRCKEILTFSNTSTCKTCKIKVCLKHRFPADHVCQKQTAA

>MdSAP28

GNCDPRKKKKPTCPVRRCKEILTFSNTSTCKTCKIKVCLKHRFPADHVCQKQTAA

>MdSAP29

RAREVAEVVSQPNRCTVCRKRVGLTG.FKCR.CGTTFCGVHRYPEKHACSFDFKT

>MdSAP30

RAREVAEVVSQPNRCTVCRKRVGLTG.FKCR.CGTTFCGVHRYPEKHACSFDFKT
